# Structural basis for the contribution of latent TGFβ binding protein to TGFβ latency and activation

**DOI:** 10.1038/s41467-026-75834-8

**Published:** 2026-08-05

**Authors:** George R. Biggin, Matthew Snee, Yu-Bai Xiao, Catherine Smedley, Alan R. F. Godwin, Rana Dajani, Holly L. Birchenough, Thomas A. Jowitt, Mark A. Travis, Alan M. Roseman, Anna Tarakanova, Clair Baldock

**Affiliations:** 1 Manchester Cell Matrix Centre, University of Manchester, Manchester, UK; 2 Division of Cell Matrix Biology and Regenerative Medicine, School of Biological Sciences, Faculty of Biology, Medicine and Health, University of Manchester, Manchester, UK; 3 School of Mechanical, Aerospace, and Manufacturing Engineering, University of Connecticut, Storrs, US; 4 Division of Immunology, Immunity to Infection and Respiratory Medicine, School of Biological Sciences, Faculty of Biology, University of Manchester, Manchester, UK; 5 Lydia Becker Institute of Immunology and Inflammation, University of Manchester, Manchester, UK; 6 Division of Molecular and Cellular Function, School of Biological Sciences, Faculty of Biology, Medicine and Health, University of Manchester, Manchester, UK; 7 Department of Biomedical Engineering, University of Connecticut, Storrs, US

**Keywords:** Cryoelectron microscopy, Growth factor signalling, Integrins, Molecular conformation

## Abstract

Transforming growth factor-β (TGFβ) is a potent cytokine that controls all aspects of cellular behavior. TGFβ is secreted in complex with its prodomain and latent TGFβ-binding protein-1 (LTBP1), forming the large latent complex (LLC), which through interaction with the extracellular matrix enables integrin-mediated activation. Although TGFβ structures are known, the influence of LTBP1 on the structure and activity of TGFβ is unknown. Here, we report the LLC cryo-EM structure comprising the LTBP1 eight-cysteine domain covalently bound to TGFβ, revealing a hydrophobic interface between TGFβ and LTBP1. Structure-guided mutagenesis shows that the interface is important for complex formation and TGFβ activity. Our structure supports a contralateral domain swapped architecture in the LLC, and simulations show that this architecture requires increased force to overcome barriers for integrin-mediated activation, while the covalent attachment of TGFβ to LTBP1 redistributes force to reduce unfolding barriers. These insights will be important for therapeutic strategies targeting TGFβ.

## Abstract (web summary)

TGFβ is an important cytokine that controls cellular behavior and its activity is tightly regulated. Here, the authors determine the structure of the large latent TGFβ complex with LTBP1, to show how LTBP1 supports latent complex formation and TGFβ activity.

## Introduction

**Fig. 1:**
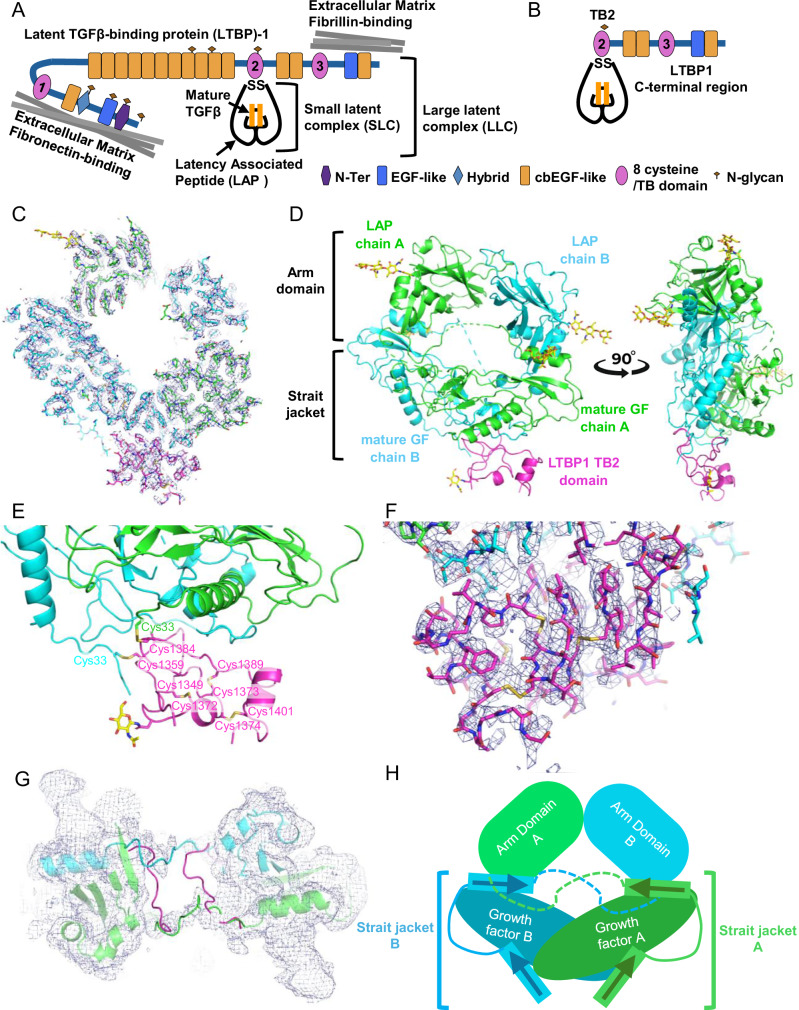
Cryo-EM structure of the LLC. **A** Schematic diagram of the SLC and LLC. The SLC is composed of the mature TGFβ homodimer encircled by LAP. The SLC is covalently cross-linked to LTBP1 through disulfide linkages to form the LLC. LTBP1 binds to fibronectin and fibrillin through N- and C-terminal regions to connect to the extracellular matrix. **B** Schematic diagram of the LLC construct used for cryo-EM. **C** Atomic model of the LLC fitted to the sharpened cryo-EM map (3.06 Å) used for modeling. **D** Ribbon representation of the atomic model of the LLC shown in two orthogonal orientations. The straitjacket and arm domains of LAP are indicated. **E** Residue Cys33 of LAP is disulfide-bonded to Cys1359 and Cys1384 in TB2. **F** Sharpened cryo-EM density map showing the TB2 domain of LTBP1. In panels C-G, the two molecules of the SLC dimer are colored green (chain A) and cyan (chain B), and the TB2 domain of LTBP1 is colored magenta. In all atom representation, the backbone is colored by chain with non-carbon atoms colored according to the CPK convention. For (**D–F**), N-linked glycans are colored yellow with non-carbon atoms colored according to the CPK convention. **G** Cryo-EM density filtered to 5 Å supports a contralateral domain-swapped architecture for the SLC with respect to the connection between the straitjacket and the arm domain (connector modeled in green and cyan), rather than an ipsilateral architecture (connector shown in pink). **H** Schematic diagram showing the contralateral arrangement, where the arm domain of LAP is contralaterally arranged to the straitjacket domain.

Transforming growth factor-β (TGFβ) is a multi-functional, potent cytokine whose temporal and spatial regulation impacts numerous cellular and tissue processes such as proliferation, differentiation and migration and thus plays key roles in development, immunity and tissue homeostasis^1^. Dysregulation of TGFβ has been reported in various autoimmune and inflammatory diseases as well as in fibrosis and cancer. Owing to the multiple roles of TGFβ, it is unsurprising that this growth factor requires intricate regulation^2^. TGFβ is synthesized with a large N-terminal prodomain referred to as latency-associated peptide (LAP), which is essential for folding and dimerization of the growth factor. In the Golgi, LAP is cleaved by furin but remains non-covalently associated with TGFβ, with this complex of TGFβ-LAP referred to as the small latent complex (SLC). Prior to secretion, the SLC is disulfide-bonded to latent TGFβ-binding protein 1 (LTBP1) in many cell types (Fig. 1A), or the membrane receptor glycoprotein A repetitions predominant (GARP) in regulatory T cells and platelets^3,4^. The TGFβ-GARP complex is responsible for immune regulation, whereas the TGFβ-LTBP1 complex, termed the large latent complex (LLC), plays a key role during development, wound healing and fibrosis^5^.

There are four members of the LTBP family, with LTBP1, −3 and −4 able to form latent complexes with TGFβ via the formation of a disulfide bond with Cys33 of LAP. LTBP2 does not associate with TGFβ, and the main role of LTBP4 is in elastic fiber formation. LTBP1 is the most common isoform, which is required for both efficient secretion of latent TGFβ and its sequestration in the extracellular matrix^6–8^. Indeed, mutation of Cys33 in LAP to serine gives rise to a phenotype similar to TGFβ1-null animals with multiorgan inflammation^9^. However, the phenotype of the LAP^C33S^ mice is not as severe as that of TGFβ-null mice, which die shortly after birth^10^, as LTBP1-independent mechanisms for TGFβ activation can occur. Once secreted from cells, TGFβ can be activated through various mechanisms, with the main physiological activator being via binding specific integrins^11^. Activation of TGFβ from the extracellular matrix-bound LLC by epithelial cells and myofibroblasts is supported by integrins αvβ1, αvβ3, αvβ5, and αvβ6^12–14^. This mechanism involves the transmission of cell traction forces via the integrin, which binds to LAP via one of two Arg-Gly-Asp (RGD) sites. This integrin-mediated force activation requires latent TGFβ to be cross-linked to LTBP1, to provide mechanical resistance against the cell traction forces and induce a conformational change in LAP that leads to liberation of active TGFβ^15^. In this mechanism, LTBP1 anchors the LLC into the extracellular matrix by binding to fibrillin or fibronectin^16,17^. This is in contrast to activation by αVβ8, which can activate TGFβ from the SLC by either protease-dependent processes^18,19^ or independently of the release and diffusion of TGFβ^20^.

Previous studies have determined the crystal structure of the SLC, revealing how LAP encircles dimeric TGFβ in a ring-shaped conformation to shield the mature growth factor from its receptors^21,22^. The crystal structures of the SLC were unable to resolve the connector region of LAP, which links the straitjacket to the arm domain, but molecular modeling supported an ipsilateral arrangement of the TGFβ prodomain, where the arm domain connects to the adjacent straitjacket^22^. Recently, however, the connector was visualized in the cryo-EM structure of the GARP-SLC^23^, which showed a contralateral domain swapped architecture with the straitjacket connecting to the arm domain on the opposite side of the molecule^24^. Understanding the correct architecture of latent TGFβ is important to understand how force is redistributed from the arm domains to the straitjacket upon integrin-mediated force activation. Indeed, previous force-probe molecular dynamics simulations, to monitor the force required and identify the major force barriers in the unfolding of LAP in integrin-mediated activation of TGFβ, were performed on the SLC in the ipsilateral arrangement. These simulations showed that the highest force barriers were associated with perturbation of secondary structure in the arm domain^25^. However, it may be that there are differences in the distribution of force with a contralateral architecture of the SLC. Moreover, whether there are structural rearrangements in the SLC upon covalent linkage to LTBP1 and precisely how LTBP1 confers latency across the LLC and regulates TGFβ activation is not fully understood. Current therapeutic strategies targeting TGFβ that focus on global inhibition of signaling often have severe toxicities^26^, but recent findings indicate that mAbs that enhance TGFβ activation from membrane-bound GARP-complexes may offer a more targeted therapeutic strategy^27^. Therefore, improved understanding of TGFβ latency and activation may facilitate the discovery of additional targeted therapeutic approaches.

Here we report the 3.06 Å cryo-EM structure of the LLC, where the eight-cysteine, TGFβ-binding (TB) domain of LTBP1 is resolved bound to the SLC. This complex reveals a hydrophobic interface supported by pi stacking interactions between the mature TGFβ and LTBP1, which are important for covalent complex formation and in regulating TGFβ activation. Our cryo-EM map provides further support that latent TGFβ has a contralateral domain-swapped architecture with respect to the connection between the straitjacket and the arm domain. Force-probe molecular dynamics simulations comparing the ipsilateral versus contralateral arrangement of the SLC show a largely similar activation profile, but with an increased force requirement for the major force barrier associated with unsnapping of the integrin-bound arm domain fastener in the contralateral configuration. Furthermore, these analyses show that covalent attachment of TGFβ to LTBP1 distributes the force required for activation to reduce the unfolding force barriers.

## Results

### Cryo-EM structure of the latent TGFβ complex with LTBP1

To determine the molecular basis of LTBP1-mediated latency of TGFβ, a complex of the SLC covalently linked to a C-terminal region of LTBP1, with an N-terminal fragment of fibrillin-1 non-covalently bound, was prepared (Fig. 1B, Supplementary Fig. 1). This complex was analysed by cryo-EM and a structure of the SLC and the covalently bound TB2 domain of LTBP1 was resolved to 3.06 Å using cryo-EM (Fig. 1C, Supplementary Fig. 2 and Supplementary Table 1), hereafter referred to as the LLC. There was only weak density for the LTBP1 domains downstream of TB2 (Supplementary Fig. 3), and fibrillin-1 was not resolved, presumably owing to the flexibility of these domains, as we have shown previously^28^. The LLC structure has the characteristic ring-like structure of the SLC but with the α1 helices of the LAP straitjacket extending down to form a covalent linkage with the TB2 domain of LTBP1, which is offset to one side of the dimer, breaking the two-fold symmetry of the SLC (Fig. 1D). Disulfide bonds form between the two Cys33 residues of the LAP dimer and cysteine residues 1359 and 1384 in LTBP1 (Fig. 1E, F). There was cryo-EM density supporting a contralateral domain-swapped architecture with respect to the connection between the straitjacket and the arm domain of LAP (Fig. 1G, H).

### The large latent complex reveals a hydrophobic interface between the mature TGFβ growth factor and LTBP1, strengthened by pi stacking interactions

**Fig. 2:**
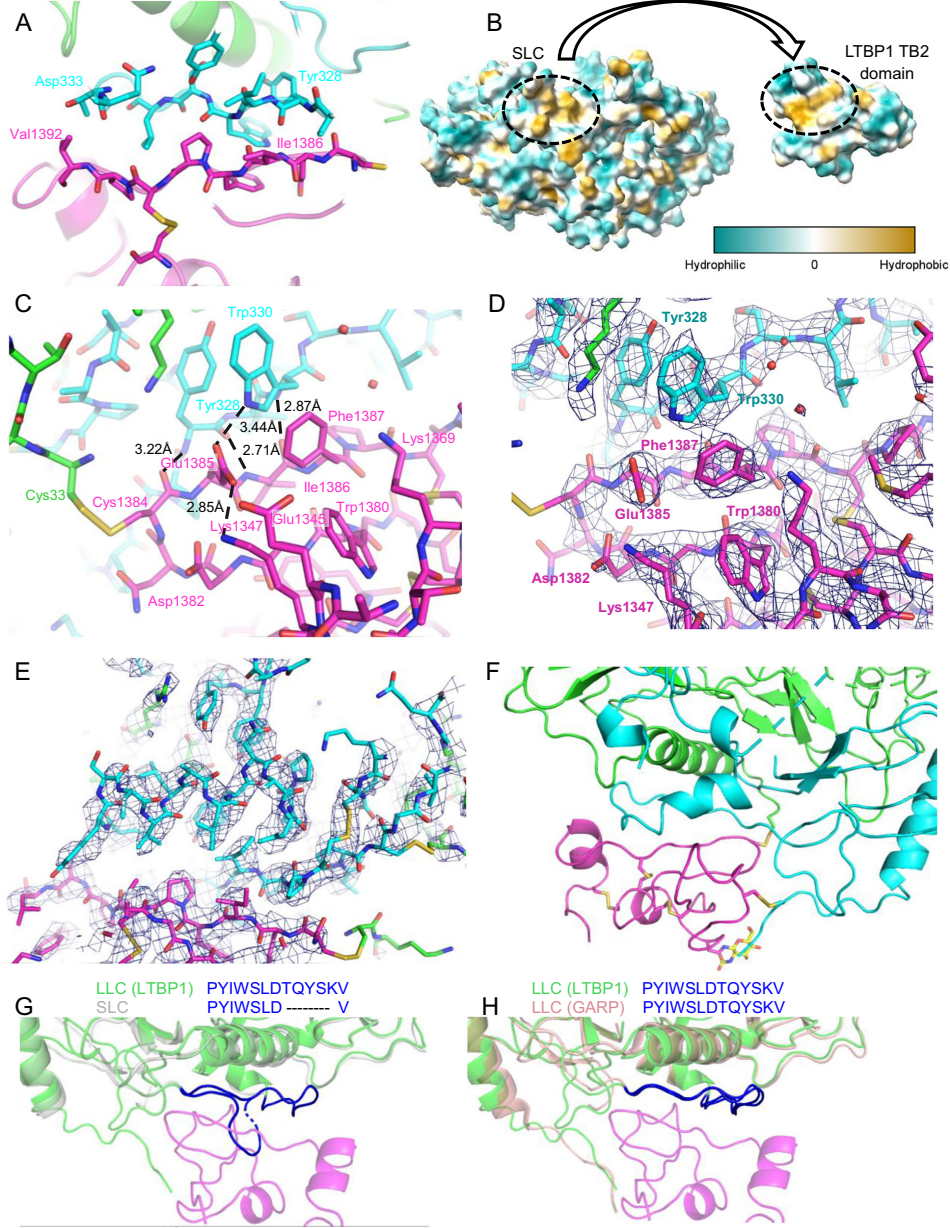
The LLC structure reveals a hydrophobic interface between TGFβ and LTBP1 strengthened by pi stacking interactions. **A** β-strand formed between the mature TGFβ growth factor and the TB2 domain of LTBP1 in the LLC. **B** Surface hydrophobicity of the interface between LTBP1 and TGFβ, where the TB2 domain of LTBP1 has been rotated outward to expose the interaction surface between TGFβ and LTBP1, highlighted by black dashed circles. Hydrophobicity was determined using the Kyte-Doolittle scale in UCSF ChimeraX^50^. **C, D** Residues interacting between the SLC and LTBP1 include a hydrophobic interface with pi stacking interactions between aromatic residues in mature TGFβ (Y328 and W330) and LTBP1 (W1380 and F1387). **E** Cryo-EM density map showing an amphipathic helical section formed in the mature TGFβ upon LTBP1 binding, which is also shown in cartoon representation (**F**). In panels A-F, the SLC is colored green (chain A) and cyan (chain B), and the TB2 domain of LTBP1 is colored magenta. In all atom representation, the backbone is colored by chain with non-carbon atoms colored according to the CPK convention. Panels (**D**, **E**) show the sharpened cryo-EM map. **G** Comparison of the binding interface between the SLC and LTBP1 from the LLC structure. The crystal structure of the SLC (PDB: 5FFO)^25^ shown in gray is superimposed on the LLC structure (green: SLC, magenta: LTBP1 TB2 domain). A region that is unresolved in the SLC crystal structure is colored blue in both models, which is ordered in the LLC. **H** The same comparison with the LTBP1-LLC structure is made with the GARP-SLC structure (PDB: 8VSC^23^) (pink), with this region (colored blue) very similar in both structures.

There are extensive interactions between the mature TGFβ growth factor and the TB2 domain, which induce a number of structural rearrangements within the growth factor. These include the formation of a continuous antiparallel β-sheet between LTBP1 and the growth factor involving TGFβ residues 328–333 and LTBP1 residues 1386–1391 (Fig. 2A). There are a number of solvent exposed hydrophobic residues within this region that form hydrophobic patches on each molecule (Fig. 2B). This hydrophobic interaction is further stabilized by pi stacking interactions between aromatic residues in TGFβ (Y328 and W330) and aromatic residues in LTBP1 (W1380 and F1387) and a network of hydrogen bonds (Fig. 2C, D). In addition, residues 334–338 of the mature TGFβ growth factor are unresolved in previously published structures of the SLC and form part of a flexible loop^22^. However, in the LLC structure, these residues fold into an amphipathic helical section (residues 336–343) positioned along the bottom of the SLC (Fig. 2E–G). This is similar in GARP-SLC structures^23,29^, where this region is more ordered upon GARP binding, so this appears to be a feature of the formation of a latent complex with either LTBP1 or GARP (Fig. 2H).

### Hydrophobic pi stacking interactions between TGFβ and LTBP1 are important for covalent complex formation and regulating TGFβ activation

**Fig. 3:**
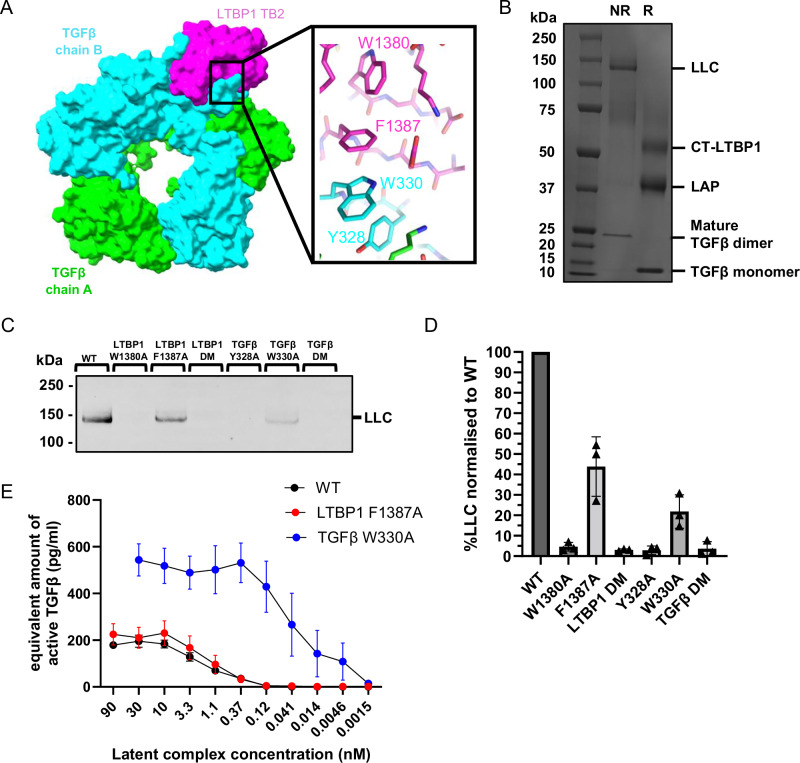
Pi stacking interactions between TGFβ and LTBP1 are important for covalent LLC formation and TGFβ activity. **A** Surface representation of the LLC with the SLC colored in green and cyan, and the LTBP1 TB2 domain in magenta. The box represents the region expanded to show the aromatic residues in the interface that were mutated to alanine. **B** Instant blue-stained SDS-PAGE showing the LLC at ~140 kDa and mature TGFβ dimer under non-reducing (NR) conditions, which runs as the separate components (C-terminal region of LTBP1, LAP, mature TGFβ monomer) under reducing (R) conditions (*n* > 10). **C** Western blot of conditioned media from wildtype (WT) and mutant LLC cell lines (DM indicates double mutant) run under non-reducing conditions. The LLC was detected using an anti-strep antibody. Equal total protein was loaded as determined by the BCA assay. A representative blot is shown (*n* = 3). **D** Band intensity analysis from western blotting of the amount of mutant LLC normalized to WT (100%). Bars represent the mean intensity, and the error bars represent the standard deviation from three biological repeats (*n* = 3). **E** TMLC TGFβ assay using recombinant WT (black), W330A (blue), and F1387A (red) LLC proteins. The relative light units were converted to TGFβ activity using a standard curve determined for active TGFβ response. The TGFβ activity of LTBP1 F1387A LLC was not significantly different from WT LLC (*P* = 0.956). TGFβ activity of TGFβ W330A LLC was significantly higher compared to WT LLC (*P* = < 0.001). Statistical significance was determined by two-way ANOVA with Tukey’s multiple comparisons test. Data represent the mean ± s.d. of three biological repeats, each calculated from the average of three technical replicates.

We hypothesized that non-covalent interactions between the mature TGFβ growth factor and LTBP1 may be important for the formation of the large latent covalent complex and potentially in regulating TGFβ activation. To test this, the aromatic residues involved in the pi stacking interaction in TGFβ (Y328 and W330) and LTBP1 (W1380 and F1387) (Fig. 3A) were mutated to alanine in the LLC construct, both as single and double mutations, and transfected into HEK293 cells. The LLC appears as a band of ~140 kDa with the mature TGFβ dimer on non-reduced SDS-PAGE, which runs as the separate components (LTBP1, LAP and the mature TGFβ monomer) on a reduced gel (Fig. 3B). The amount of wildtype (WT) and mutant proteins incorporated into the LLC was assessed by western blotting using an antibody against the strep-tag on LTBP1. LTBP1 W1380A and TGFβ Y328A mutations and both the LTBP1 and TGFβ double mutant (DM) constructs prevented formation of the covalently linked LLC (Fig. 3C). The LTBP1 F1387A and TGFβ W330A mutants were able to form the LLC, however at reduced levels compared to wild type (Fig. 3C). The LTBP1 F1387A mutant formed the LLC at 43.85% of WT levels, and the TGFβ W330A mutant forming 21.87% of WT levels of LLC (Fig. 3D). The amounts of the SLC and LTBP1 secreted for all mutant constructs was unchanged, indicating that the mutations did not affect protein folding or secretion just the ability to form the LLC (Supplementary Fig 4).

Although able to form the covalent LLC at reduced levels, it may be that the F1387A and W330A mutations weaken the interaction between LTBP1 and TGFβ, potentially destabilizing the LLC, making it more active. To investigate this, the F1387A and W330A LLC proteins were purified for cell-based TGFβ activity assays using the transformed mink lung cells (TMLC)^30^. Recombinant LLC proteins were exogenously added to TMLC cells carrying a TGFβ-responsive element alongside a luciferase reporter gene. These assays showed no significant difference in TGFβ activity between WT LLC and LLC with LTBP1 F1387A mutation (Fig. 3E). However, the LLC with mutation W330A in TGFβ was significantly more active compared to WT, suggesting the aromatic residues in TGFβ are important for the formation of the LLC and activity (Fig. 3E). Together, these results indicate that the aromatic residues in this interface between LTBP1 and TGFβ are important for the formation of the LLC and destablization of this region may expose the mature growth factor making it more active.

### LTBP1 binding to SLC does not change affinity for αvβ6

**Fig. 4:**
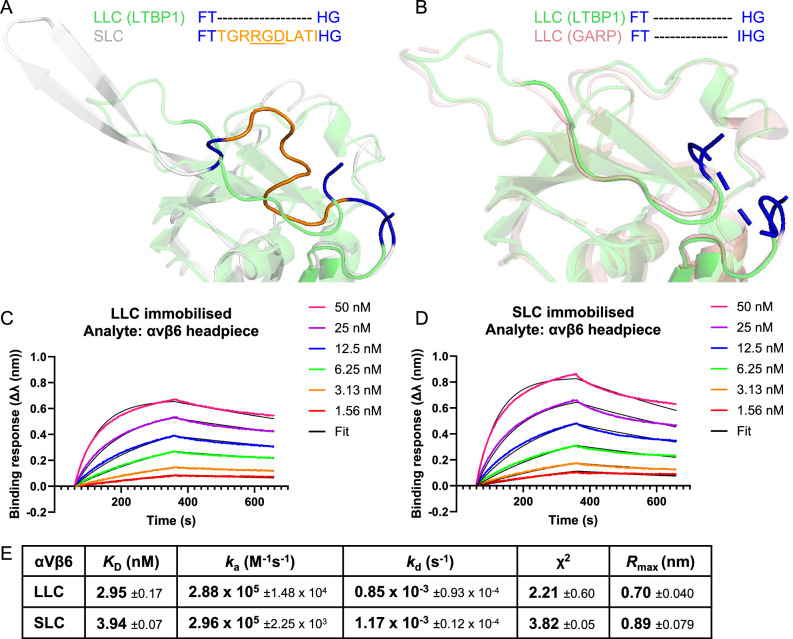
RGD loop conformation and binding to integrin αvβ6 between the SLC and LLC. **A** Comparison of the RGD region in the arm domain of LAP in the cryo-EM LLC (LTBP1) structure (green) with the SLC structure (5VQP)^22^ (gray) and **B** the LLC (GARP) structure (PDB: 8VSC)^23^ (pink). Residues on either side of the RGD region are colored blue, and the RGD region is colored orange (in the LTBP1-LLC structure, the RGD region is in a solvent-exposed flexible loop and unresolved). **C** Sensograms of the binding of the LLC or **D** SLC to the headpiece of integrin αVβ6. The LLC or SLC were immobilized onto streptavidin-coated biosensors, and the binding kinetics of the analyte αVβ6 were monitored using BLI on OctetRED96. The measured responses, using a concentration range of 0–50 nM, were fitted to a 1:1 Langmuir binding model (dark gray lines). Representative sensograms are shown (*n* = 3). **E** Calculated kinetic parameters for binding of the LLC or SLC to integrin αVβ6 headpiece shown as the mean of three experiments (*n* = 3). The error is ± standard deviation.

In the cryo-EM structure of the LLC, the RGD region of LAP is unresolved, forming a solvent-exposed flexible loop, in contrast to structures of the SLC (Fig. 4A)^22^. This arrangement of the RGD loop in the LTBP1-latent complex is similar to structures of the GARP-SLC latent complex^23,29^ (Fig. 4B). To test whether LTBP1 promotes activation of latent TGFβ by increasing the affinity of integrin binding, Octet-biolayer interferometry (BLI) binding analysis was performed to compare the affinity of the LLC and the SLC to the integrin αVβ6 headpiece (Fig. 4C, D, Supplementary Fig. 5). However, kinetic analysis showed no significant difference in the amount binding or the affinity of the LLC or SLC for integrin αVβ6 (Fig. 4E) indicating that binding of the SLC to LTBP1 does not change the interaction with αVβ6.

### Molecular dynamics simulations comparing force-mediated TGFβ activation in ipsilaterally and contralaterally arranged TGFβ

The LLC cryo-EM map provides further support for a contralateral domain-swapped architecture for latent TGFβ, with the straitjacket being on the opposite side of the molecule to the RGD-containing arm region. This is in agreement with the recently reported cryo-EM structure of the GARP-SLC complex^23,24^ but contrasts with the ipsilateral architecture previously modeled in X-ray crystallographic structures of the SLC^21^ and GARP-SLC^29^. The arrangement of the straitjacket and arm regions could affect the unfolding barriers and force required for integrin-mediated TGFβ activation, as previous simulations used the ipsilaterally arranged TGFβ to predict these^25^.

**Fig. 5:**
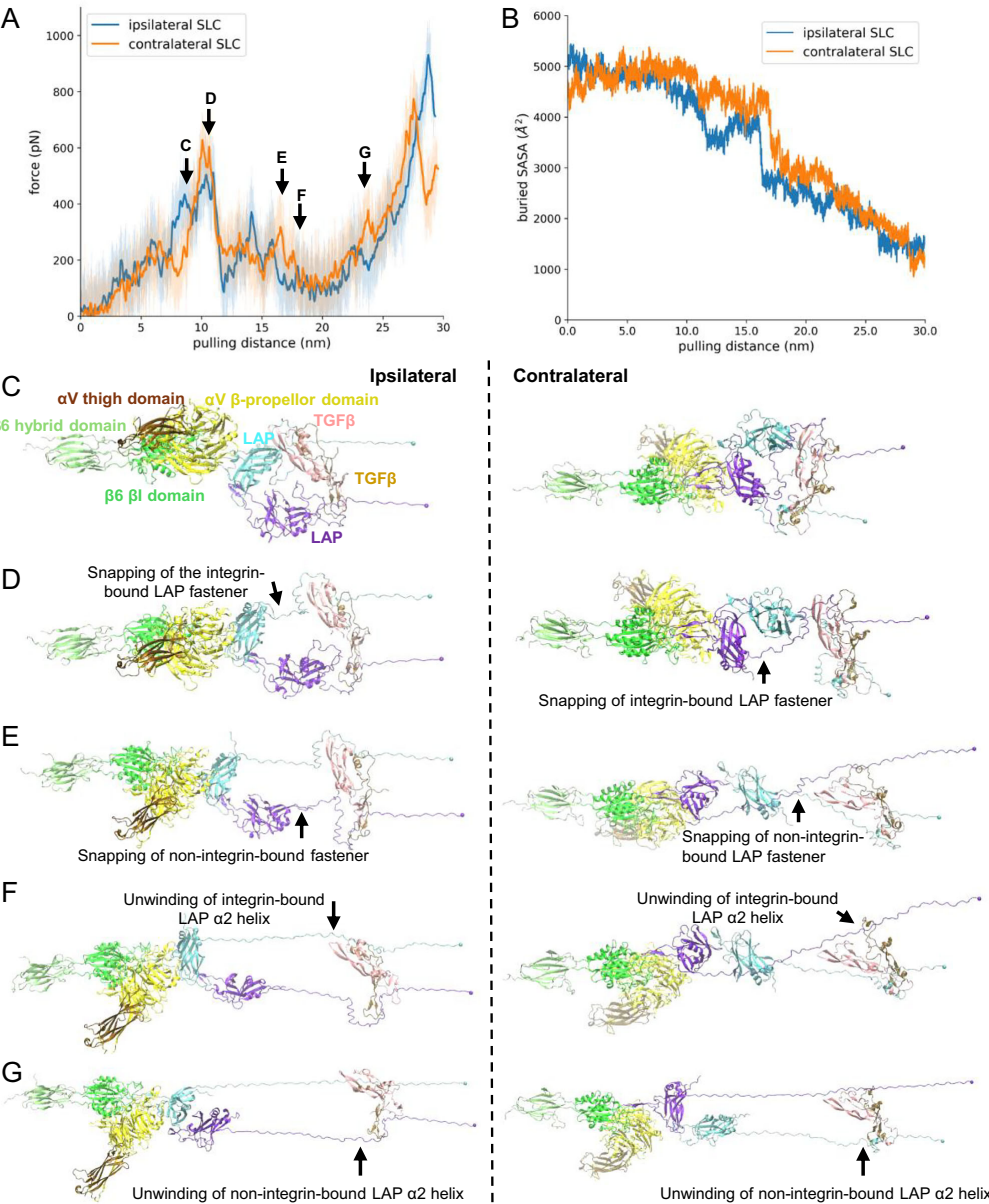
Simulation of integrin-mediated activation of the SLC with ipsilateral or contralateral arrangement. **A** Using force-probe molecular dynamics simulation, pulling at 0.05 nm/s on the center of mass (COM) of the integrin β6 hybrid domain, which was resisted by LAP Cys33 residues. Force displacement curve with pulling distance (nm) plotted against force (pN). The shading shows the raw data, and the solid lines represent a sliding-window average. Arrows mark events corresponding to panels (**C**–**G**). **B** buried solvent accessible surface area (SASA) (Å^2^) between LAP and growth factor against pulling distance (nm). In (**A**, **B**), ipsilateral: blue, contralateral: orange. **C–G** Snapshots from ipsilateral and contralateral simulations at pulling distances indicated by corresponding arrows in (**A**). Green: integrin β6 βI and hybrid domain, yellow: integrin α_V_ β-propellor domain, brown: integrin α_V_ thigh domain, cyan and lilac: LAP domains, beige and pink: TGFβ growth factor monomers.

Therefore, using force-probe molecular dynamics simulations, the effect of the contralateral domain architecture on integrin-mediated TGFβ activation was investigated by replicating molecular dynamics simulations performed by Dong et al.^25^. The published structure of integrin αVβ6 in complex with the SLC^25^ (PDB: 5FFO) was used, with unresolved loops built using MODELER^31^, and the loops connecting the LAP arm and straitjacket domains built either ipsilaterally or contralaterally. The integrin β6 hybrid domain is not present in the SLC-integrin structure, so this domain was merged in using the structure of αVβ6 with RGD peptide (PDB:4UM8)^32^ as a template. Molecular dynamics simulations were performed by pulling on the center of mass (COM) of the integrin β6 hybrid domain at 0.05 nm/ns, which was resisted by the Cα atoms of LAP Cys33 residues. The ipsilateral simulations recapitulate the work previously reported by Dong et al^25^, showing four key force barriers which are overcome leading to TGFβ release (Fig. 5, Supplementary Fig 6). These force barriers correspond to the sequential unsnapping of the two LAP arm domain fasteners, which fasten the arm domains to the straitjacket, followed by sequential unwinding of the two α2 helices of the straitjacket domains (Supplementary Figs 6, 7). The arm domains of LAP, in contrast to the prodomains of other TGFβ superfamily members such as activins, BMPs and GDFs, are linked by a disulfide bond in the bowtie motif^33^, which results in the pulling force on one arm domain being transmitted to the other domain, as seen in the molecular dynamics simulations (Fig. 5). We hypothesized that a contralateral architecture may affect how force is redistributed from the arm domains to the straitjacket upon integrin-mediated force activation. In the contralateral simulation, the unfolding events of LAP, including the order of these events and total energy required for TGFβ release is largely similar to the ipsilateral conformation of the SLC (Fig. 5, Supplementary Figs. 8, 9). However, our simulations do indicate that the contralateral arrangement of the SLC requires increased force to overcome the major force barrier in the mechanical activation of TGFβ, associated with the unsnapping of the integrin-bound LAP fastener.

### The contribution of LTBP1 in the resistance of force through the LLC from force-probe molecular dynamics simulations

**Fig. 6:**
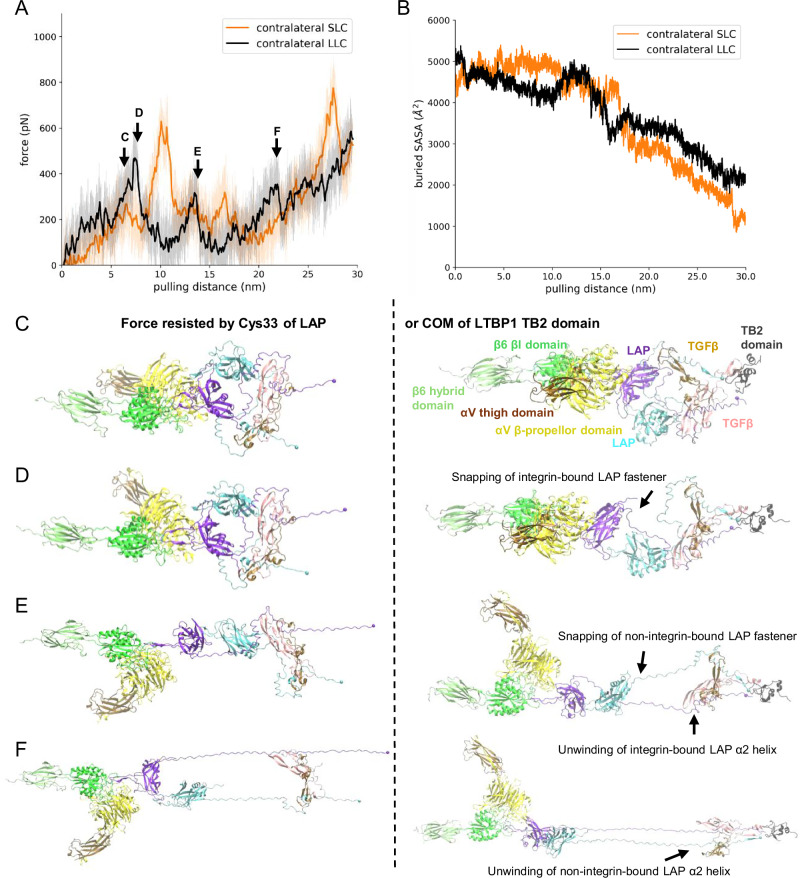
Simulation of integrin-mediated activation of the LLC. **A** Force-probe molecular dynamics simulation pulling at 0.05 nm/s on the center of mass (COM) of the integrin β6 hybrid domain, which was resisted from the COM of the LTBP1 TB2 domain, compared to the simulation of the SLC. Force displacement curve with pulling distance (nm) plotted against force (pN). The shading shows the raw data, and the solid lines represent a sliding-window average. Arrows mark events corresponding to panels (**C–F**). **B** buried solvent accessible surface area (SASA) (Å^2^) between LAP and growth factor against pulling distance (nm). In (**A**, **B**), SLC: orange, LLC: black. **C–F** Snapshots from the SLC and LLC simulations at pulling distances indicated by corresponding arrows in (**A**). Green: integrin β6 βI and hybrid domain, yellow: integrin α_V_ β-propellor domain, brown: integrin α_V_ thigh domain, cyan and lilac: LAP domains, beige and pink: TGFβ growth factor monomers, gray: TB2 domain.

To determine if covalent linkage of LAP to LTBP1 results in changes in the unfolding events of LAP and the force required for TGFβ release, further force-probe molecular dynamics simulations were performed. Molecular dynamics simulations of the LLC were performed similarly to the method described above, but resisting from the COM of the LTBP1 TB2 domain rather than from the cysteine residues in LAP (Supplementary Fig. 10). These simulations at 0.05 nm/ns pulling speed show that the LLC is more readily activated upon force application (Fig. 6, Supplementary Fig. 8B). This difference may arise from the additional confinement imposed by attachment of the LTBP1 TB2 domain, which leads to incomplete relaxation of the LAP structure. Consequently, the interaction between the fastener and the growth factor is relatively unstable, requiring a shorter pulling distance and lower pulling force to rupture compared with the more stable fastener binding observed in the SLC model (Supplementary Figs. 9A, 11A). The simulations also show there are fewer major force barriers in integrin-mediated unfolding of LAP in the LLC when force is applied across the TB2 domain (Fig. 6A). This may be because the LTBP1 TB2 domain is acting as a mediator that distributes force more evenly between the two LAP chains by imposing rigid confinement on the LAP monomers, thereby establishing a favorable pulling orientation and more efficiently coordinating the snapping and unbinding events between LAP and the growth factor. In the simulations with the force just across the SLC, α1 helices unwind first, followed by snapping of the integrin-bound LAP fastener, producing the initial major force event. This is followed by snapping of the non-integrin-bound LAP fastener, then unwinding of the integrin-bound LAP α2 helix and finally unwinding of the non-integrin-bound LAP α2 helix (Supplementary Figs 7, 9). In contrast, in the LLC simulations, the unwinding of the α1 helices occurs much later. Unwinding of the integrin-bound LAP α1 helix in the LLC simulation occurs alongside the events corresponding to the snapping of the non-integrin-bound LAP fastener and the unwinding of the integrin-bound LAP α2 helix. The unwinding of the non-integrin-bound α1 helix occurs alongside the unwinding of the α2 helix in the same LAP. Furthermore, in the LLC simulations, snapping of the non-integrin-bound LAP fastener occurs concurrently with the unwinding of the integrin-bound α2 helix (Supplementary Fig. 11). This cooperative unwinding pattern in the LLC may illustrate how covalent linkage of LAP to LTBP1 coordinates these force-dependent events and more efficiently transmits the applied force to upstream structural elements of LAP—namely the latency lasso (a loop that connects the α1 and α2 helices), the α2 helix, and fastener. Such coordinated force distribution may help explain why these force-resistant elements unfold to release TGFβ in the LLC.

## Discussion

Here, we report the cryo-EM structure of the LTBP1-LLC, which resolves the TB2 domain of LTBP1 covalently bound to the SLC. Our structure details several conformational changes throughout latent TGFβ resulting from LTBP1 binding and includes the identification of an interface between the growth factor and LTBP1. Indeed, in structures of the SLC alone, there is a flexible loop in TGFβ, where residues 334–338 are unresolved^22^, whereas, in the LTBP1-LLC structure, these residues form an amphipathic helical structure which is involved in a hydrophobic interface with LTBP1. This interaction is mediated by contiguous pi-pi stacking between aromatic residues of TGFβ (Y328 and W330) and LTBP1 (W1380 and F1387). This region in TGFβ also forms an interface with GARP, as shown previously^29^, and while W330 is involved in the interaction with both GARP and LTBP1, there are interesting differences between how LTBP1 and GARP achieve complementarity with the same conformer of TGFβ. In LTBP1, there is an extensive hydrophobic interaction where the helical structure in TGFβ is enclosed by a loop in LTBP1 (residues 1352–1358) along with the aforementioned pi-pi stacking interaction. Whereas in GARP, W330 interacts with the side chains of R206 and C227, and the helical structure with M186 and V184^23,29^. The more extensive hydrophobic interface in LTBP1 may reflect the need for increased binding efficiency as the interaction with TGFβ is mediated by a smaller interaction domain.

Previous studies proposed a hydrophobic interaction between LTBP1 and TGFβ via a TGFβ binding motif (residues ^1385^EIFP^1388^, which included F1387), located between the 6^th^ and 7^th^ cysteine residues of the LTBP1 TB2 domain. Specifically, this FP dipeptide insertion was shown to render C1359 and C1384 more available for protein-protein interactions^34^. This was thought to be due to an interaction between LTBP1 and LAP^35^, whereas our cryo-EM structure shows that LTBP1 residues 1386-1391, which includes the IFP residues in the proposed TGFβ-binding motif^34,35^, participate in a hydrophobic interaction but with residues 328–333 in the mature TGFβ growth factor. There are also structural differences between the TB2 domain of LTBP1 in the latent complex and the NMR structure of this domain alone. Primarily, these relate to the conformation and disulfide bonding of the extended loop containing C1359 and C1384, which form intermolecular disulfide bonds with LAP in the latent complex, but form an intramolecular disulfide bond in the absence of TGFβ (Supplementary Fig. 12).

To investigate the importance of hydrophobic and pi stacking interactions between LTBP1 and TGFβ for covalent complex formation, we performed mutagenesis studies to analyse their effect on the formation of the covalent LLC. Mutating residues involved in pi stacking between LTBP1 (W1380 and F1387) and TGFβ (Y328 and W330) either significantly reduced or abolished covalent LLC formation. Specifically, mutating residues that were further away from the interaction surface and are core to TGFβ (Y328) and LTBP1 (W1380) completely abolished the LLC formation. These residues are highly conserved and are present across all TGFβ and LTBP1 isoforms. As such, these residues may play a crucial role in stabilizing the secondary structure conformation surrounding the interaction interface, which may be crucial for forming non-covalent interactions required for covalent LLC formation. However, mutating residues W330 (TGFβ) and F1387 (LTBP1) still allowed LLC formation, but the amount of the LLC formed was markedly reduced. F1387 in LTBP1 is not conserved in the other members of the LTBP family, and TGFβ W330 is only present in TGFβ1 and TGFβ2. This suggests that although important, pi-stacking interactions are not the sole requirement for the LLC formation, and that perhaps the cooperative effect of hydrophobic interactions, strengthened by pi-stacking, allows for efficient formation of the covalent LLC. For the latent complex that is formed, the F1387A LTBP1 mutation does not alter TGFβ activity, whereas TGFβ with the W330 mutation increases activity. Interestingly, F1387 forms a hydrogen bond through its backbone atoms with W1380, so mutation of the F1387 side chain is unlikely to disrupt this hydrogen bond, and its interaction with W1380 may be retained. It is also possible that there could be a structural rearrangement in the TB2 domain, so that W1380 could interact with W330 in TGFβ to compensate for the mutation of F1387. Whereas for W330 in TGFβ, as well as interacting with LTBP1, this region of the growth factor is adjacent to the α1 helix of the adjacent chain of LAP, thus the mutation may also destabilize this interaction to affect the latency lasso of LAP.

The RGD-containing loop in the arm domain of LAP is solvent-exposed and flexible in the LTBP1-LLC structure, which differs from the conformation observed in the crystal structures of the SLC. Thus, binding analyses were performed to determine if there was a difference in the binding affinity for integrin αVβ6. However, our binding studies show that the on rates for the binding between the LLC and SLC to integrin are very similar, and there is no difference in the affinity between the LLC and SLC for integrin αVβ6, so it could be that this region is flexible and the specific conformation of the RGD-loop does not alter the binding kinetics. Or it may be that the RGD-exposed conformer seen in our cryo-EM structure is more commonly observed in solution, whereas the conformer observed in crystal structures of the SLC is less predominant in solution and promoted by the crystallization conditions or crystal packing.

Our structure was also used to validate observations from previous studies regarding potential interactions present in the LLC. Interestingly, Lack et al. identified five negatively charged residues (E1348, D1355, D1360, D1382, E1385) in the TB2 domain of LTBP1, which were suggested to form electrostatic interactions with LAP. It was proposed that these residues may be important in LTBP1 molecular recognition of LAP, by limiting non-specific bond formation of LTBP1 cysteines involved in LAP covalent linkage^34^. FRET analysis of LTBP1 mutant proteins with the SLC revealed that efficient complex formation was most reliant on D1382 and E1385. However, our LLC structure does not show interactions between these two residues and LAP. Instead, these residues may play a structural role, stabilizing the secondary structure conformation of the region surrounding C1384 and facilitating disulfide bond formation with LAP. Our structure does, however, show an electrostatic interaction within LTBP1 between E1385 and K1347 (2.85 Å).

Force activation of TGFβ is mediated by integrin binding to the RGD loop in LAP, which enables the application of tensile force to unmask the TGFβ growth factor receptor binding sites by unfolding of LAP. The steps between LAP unfolding and TGFβ receptor binding are not yet defined molecularly, but TGFβ is in a different conformation when in complex with LAP compared to when it is bound to its receptors, and LAP also blocks receptor binding sites to provide latency^21,36^. The type II receptor binding site is blocked by the latency lasso and the type I receptor binding site is blocked by the straitjacket α1 helix, the fastener and the arm domain^21^. Mutation of key aromatic residues in the fastener, to release the fastening of the straitjacket to the arm domain, resulted in spontaneous TGFβ activation and, conversely, engineering a disulfide bond to covalently fasten the straitjacket prevented integrin-mediated activation^21^. The main force peaks observed in our SMD simulations were associated with the unsnapping of each fastener and unwinding the α2 helices in the interfaces between the arm domain and the growth factor. In agreement with Dong et al., the simulations predict that these are the most force-resistant elements^25^ and releasing the fastening of the straitjacket would allow TGFβ activation by revealing receptor binding epitopes.

Previously published structures of the SLC have reported an ipsilateral conformation of TGFβ with regard to the straitjacket being ipsilateral to its RGD-containing arm^21,22^. However, the recent cryo-EM structure of the GARP-SLC complex supports a domain-swapped contralateral arrangement between the straitjacket and arm domain^24^. Our LTBP-LLC cryo-EM map also shows a contralateral architecture, which raised the question of how forces might be dissipated across the complex with a contralateral architecture during integrin-mediated activation of TGFβ. In order to determine whether previous molecular dynamics simulations, which used an ipsilateral conformation of the SLC to model the force-mediated unfolding of LAP and release of TGFβ^25^, would be substantially different in the contralateral arrangement, these simulations were performed. Simulations suggest that a contralateral architecture results in an increased force requirement for the major force barrier associated with the unsnapping of the fastener corresponding to the integrin-bound LAP arm domain. However, the unfolding events and the order of these events, as well as the total energy required for TGFβ release, are comparable to those of ipsilaterally arranged latent TGFβ.

Additionally, we performed further molecular dynamics simulations to investigate the effect of LTBP1 on force-mediated activation of TGFβ. Our results indicate that TGFβ is more readily activated when force is transmitted through LTBP1, as reflected by both a reduced number and magnitude of force barriers during mechanical activation. Consequently, force-resistant elements within LAP unfold more readily, facilitating the release of TGFβ. This behavior may arise because LTBP1 within the LLC serves as a more rigid, force-resistant anchor due to its high density of disulfide bonds. This rigidity further constrains the terminal regions of LAP, inducing a balanced pulling geometry that distributes mechanical load across both LAP monomers. As a result, the initial unwinding of the α1 helix is suppressed, thereby enhancing force concentration on upstream components of the straitjacket domain, including the latency lasso, α2 helix, and fastener. Together, these simulations highlight the critical role of LTBP1 as a mechanical anchor in enabling efficient integrin-mediated activation and release of TGFβ. It is important to note that these simulations rely on several assumptions and simplifications inherent to molecular dynamics approaches. For example, the choice of pulling velocities, positional restraints, and the treatment of unresolved regions in the protein complexes may influence the observed mechanical pathways. Consequently, while our results suggest that conformational changes in LAP and force transmission through LTBP1 facilitate TGFβ release, these causal relationships are inferred directly from computational observations. Some caution should therefore be exercised when extrapolating these findings to physiological conditions.

We further acknowledge several limitations in capturing the full complexity of the in vivo environment. First, the applied pulling direction in our simulations is defined and fixed, whereas in vivo force orientations are heterogeneous and dynamically regulated by the cellular context, including cytoskeletal attachments and extracellular matrix constraints. Experimental studies have shown that forces applied to cell surface receptors can propagate through receptor–cytoskeletal linkages and sometimes cause intracellular dissociation prior to rupture of the extracellular bond^37^. Similarly, in the context of TGFβ, the mechanical stress applied to the LAP–LTBP1 complex may propagate through multiple protein domains, triggering conformational changes that facilitate growth factor release. Therefore, the rupture pathways observed in our simulations likely represent a subset of physiologically accessible force transmission routes. Furthermore, the simulations focus on an isolated protein complex and do not account for cooperative effects arising from multivalent interactions, clustering, or neighboring molecules. In living cells, multiple molecular linkages may act cooperatively to modulate force propagation, dissociation kinetics, and mechanical stability, potentially altering the efficiency or timing of TGFβ activation. Cell-surface constraints, including membrane anchoring, spatial confinement, and steric hindrance from surrounding proteins or glycocalyx components, are not explicitly modeled. These factors could influence both the magnitude and directionality of forces, as well as the accessibility of conformational transitions required for TGFβ release. Taken together, while our simulations provide mechanistic insight into force-induced conformational changes in the LAP–LTBP1 complex, some caution should be exercised when extrapolating these results directly to physiological conditions. Nonetheless, analogies with receptor–cytoskeletal force propagation observed in other molecular systems^37^ suggest that nanoscale force transmission can have significant functional consequences, supporting the plausibility of mechanically mediated TGFβ activation.

In summary, we report using cryo-EM the structure of the LTBP1-LLC, resolving the TB2 domain of LTBP1 covalently associated with latent TGFβ. The structure reveals a hydrophobic interaction between mature TGFβ and LTBP1, which is mediated by pi stacking interactions and the importance of these interactions on the LLC formation and TGFβ activation is demonstrated. Furthermore, through molecular dynamics simulations, we highlight the importance of LTBP1 in regulating TGFβ activation. Our work contributes to the understanding of TGFβ latency and activation and supports progress towards more targeted, effective therapeutic strategies to address TGFβ dysfunction.

## Methods

### Protein expression and purification

The LLC was comprised of the SLC (residues 30–390) and a C-terminal fragment of LTBP1 (CT-LTBP1) (residues 1335–1720) in the dual expression vector pBudCE4.1 (*Thermofisher*) with a C-terminal TwinStrep tag on CT-LTBP1 and an N-terminal Flag tag on the SLC^38^. The LLC was expressed in stably transfected Human embryonic kidney (HEK) 293-EBNA cells (ATCC, RRID:CVCL_6974) grown in CELLMASTER 2125 cm^2^ cell culture roller bottles (*Greiner-Bio-One*) in DMEM/F12 (*Sigma*) supplemented with 2 mM L-Glutamine (*Sigma*), 10% (v/v) fetal calf serum (FBS) (*Gibco*), 100 units/ml penicillin and 50 µg/ml streptomycin (*Sigma*) and 1 mg/ml Gentamycin (G418) (*Thermofisher*) at 37 °C in 5% CO_2_. At confluency, expression medium (DMEM, supplemented with 2mM L-Glutamine, 100 units/ml penicillin and 50 µg/ml streptomycin) was added to cells and collected every 4 days for a maximum of 4 collections. The human integrin αv headpiece (1–594) and the β6 headpiece (1–474) were cloned into pBudCE4.1 utilizing Addgene plasmids Integrin-αv Headpiece M400GC (RRID:Addgene 159637) and Integrin-β6 Headpiece I270C (RRID:Addgene 159638), which were a gift from Timothy Springer^32^. The αv headpiece contains a C-terminal acid coiled coil and Twin-Strep tag, whereas the human β6 headpiece has a C-terminal base coiled coil and His tag. Integrin αVβ6 headpiece and the LLC mutants were expressed by transiently transfecting Expi293F HEK suspension cells (catalog number: A14527 *Thermofisher*) according to the user guide. Cells were cultured at 8% CO_2_ at 130 rpm in Expi293 expression medium (*Thermofisher*) supplemented with 50 units/ml penicillin and 50 µg/ml streptomycin. Collected expression medium was sterile filtered using a 0.45 μm membrane filter (*Merck*), and the LLC and αVβ6 were purified by Strep-Tactin affinity chromatography. Biotin blocking solution (BioLock, *IBA Lifesciences*) was added to expression media at 1 ml/L for EBNA media and 20 ml/L for Expi media for 30 min. Expression media was incubated with Strep-Tactin XT resin (*IBA Lifesciences*) for 16–18 h at 4 °C. Resin was washed with size exclusion chromatography (SEC) buffer (20 mM HEPES, 150 mM NaCl, pH 7.4) and eluted with biotin buffer (20 mM HEPES, 300 mM NaCl, 75 mM biotin, pH 7.4). Elutions were concentrated by centrifugation using a Vivaspin concentrator (*Sartorius*, cut-off 30 kDa) and purified using an S200i column (*Cytiva*) in SEC buffer. Recombinant human fibrillin-1 fragment PF3 (residues 1–722) in the pCEP-pu/AC7 vector was expressed and purified as previously described^39^.

### Mutagenesis and secretion assays

Mutations were introduced into TGFβ (single mutations Y328A; W330A, and the double mutation Y328A and W330A) and CT-LTBP1 (single mutations W1380A; F1387A, and the double mutation W1380A and F1387A) by mutagenesis. Proteins were transfected into HEK293-EBNA cells, and the media were collected. The levels of secretion of TGFβ and LTBP1 were assessed by western blotting the media using antibodies against the StrepII tag (*IBA Life Sciences*: 2-1507-001 (lot no. 1507-0066) 1:2000 dilution) on CT-LTBP1 and the FLAG-tag (*Cell Signaling Technology*: 14793S (Clone D6W5B; lot number 7) 1:10,000 dilution) on TGFβ. Equal total protein was loaded as determined by the BCA assay, and the band intensity analysed. The amount of mutant LLC was normalized to the wild-type protein. Three independent biological repeats were performed.

### TGFβ Bioassay

TMLCs^30^ from Professor Dan Rifkin (New York University, Department of Medicine, Langone Health, USA) were seeded 16,000 cells/well in 100 μl DMEM + 10% FCS and allowed to attach for 2 h. Medium was replaced with 100 μl/well of the same medium with or without recombinant protein conditions or controls, and cultured for 16–20 h. Cell layers were washed with 50 μl PBS and lysed using 22 μl, 1x reporter lysis buffer (*Promega*) from 5x, and lysates were stored at −80 °C for at least 1 h. Lysates were transferred into white 96-well plates (*ThermoFisher*) 20 μl/well. Reporter substrate (*Promega*) was added, 75 μl/well, and luminescence was measured using a Flexstation 3 plate reader. Lysates were assayed for luciferase activity as described^30^. Amounts of active TGFβ were calculated using an active TGFβ standard curve. Three independent biological repeats were performed. For each biological repeat, three technical replicates were performed and averaged to yield a single value.

### Bio-layer interferometry

Bio-layer interferometry (BLI) experiments were performed at 37 °C using an Octet RED96e system (Sartorius) with shaking at 1000 rpm and data collection at 10 Hz. Streptavidin biosensors were hydrated in assay buffer (10 mM HEPES, 150 NaCl, pH 7.4, 0.005% (v/v) Tween-20) for 30 min then loaded using 10 µg/mL of biotinylated LLC or SLC in assay buffer to a shift threshold of 0.5 nm. After establishing a baseline in assay buffer supplemented with 1 mM MgCl_2_ and 1 mM CaCl_2_ (kinetics buffer), sensors were dipped into a two-fold dilution series of analyte αVβ6 (0 to 50 nM) in kinetics buffer for 300 s to measure association, followed by incubation in kinetics buffer for 300 s to monitor dissociations. Data processing, including double reference subtraction and Savitzky-Golay filtering, was conducted using Octet Data Analysis HT software. Kinetic rate constants and the equilibrium dissociation constant were determined by globally fitting the sensorgrams to a 1:1 binding model. All experiments were performed in triplicate, and the quality of the fit to the binding data was assessed by χ^2^.

### Cryo-EM sample preparation and data collection

Purified LLC and the fibrillin-1 PF3 fragment were complexed by mixing at a x4 molar excess of PF3 for 3 h at RT. Excess PF3 was removed by Strep-Tactin affinity chromatography, utilizing the TwinStrep tag on CT-LTBP1. Strep-Tactin XT resin was added (*IBA Lifesciences*) for 16–18 h at 4 °C. Resin was washed with SEC buffer and eluted with biotin buffer (20 mM HEPES, 150 mM NaCl, 75 mM biotin, pH 7.4). The resulting protein complex was 1 mg/ml, and complex formation was confirmed by SDS-PAGE. Cryo-EM grid preparation was performed as follows. First, 3 μl of the protein complex was applied onto glow-discharged holey carbon Quantifoil R1.2/1.3 grids for 5 s and blotted for 3 s before vitrification in liquid ethane. A Vitrobot Mark IV (FEI) was used for plunge-freezing samples, where the chamber was set to 100% humidity and 4 °C. Grids were screened on a Glacios TEM (200 kV) equipped with a Falcon 3EC direct electron detector. Cryo-EM data were collected using a Titan Krios G2 cryoEM equipped with a Gatan Falcon 4i detector and TFS Selectris X energy filter operating at an accelerating voltage of 300 kV at the Electron Bio-Imaging Center (eBIC), UK. Movies were recorded at a magnification of 165,000x to give a pixel size of 0.72 Å/pixel and a defocus range of −2.5–−0.75 µm and a total dose of 50 e^−^/Å^2^ per movie, which were saved as EER format. The final data set comprised 30,000 movies.

### Single-particle data processing

Single-particle analysis was performed using cryoSPARC v2^40^. Movies were motion corrected using patch-based motion correction and up-sampled by a factor of 2, followed by patch-based CTF estimation. Initially, a subset of 500 images was used to pick 190,358 particles using blob picker. These particles were then extracted with 768 × 768 pixel box and were down-sampled to a 128 × 128 pixel box, which gave a pixel sampling of 2.16 Å/pixel before 2D classification to generate templates for template-based particle picking. Template-picker was used on the full data set and picked 19,164,772 particles. This particle set was then subjected to iterative 2D classification, removing bad particles such as ice contamination or multiple particles, until secondary structure was evident. These 13,022 particles were then used to train a Topaz picking model^41^, which picked 1,306,626 particles. Further 2D classification was used to produce a set of 404,514 particles, which were used for 3D ab-initio model generation. The ab-initio class with the highest resolution was used for iterative homogeneous and heterogeneous refinement, yielding a set of 22,159 particles, which were used to train a new Topaz model. Topaz picked 4,948,982 particles, which were again subjected to iterative rounds of 2D and 3D classification, generating a particle stack of 243,204 particles. There was only weak density for domains downstream of TB2 in LTBP1 (Supplementary Fig 3). No density was observed for fibrillin-1, presumably due to the flexibility of this modular multidomain protein. To remove heterogeneity due to the flexibility of the LTBP1, a local mask which encompassed the SLC and TB2 of LTBP1 was used for local refinement. When reconstructions reached Nyquist resolution, particles were re-extracted in a 768 × 768 pixel box and were downsampled to 256 × 256 pixels with a pixel sampling of 1.08 Å/pixel. This process was repeated iteratively until a cryo-EM map of near-atomic resolution was achieved (see workflow in Supplementary Fig. 2). The final cryo-EM map was produced through a custom-masked local refinement generated from a heterogeneous refinement class, which yielded the final cryo-EM map of 3.06 Å resolution (FSC threshold 0.143; Supplementary Fig. 2, Supplementary Table 1). The final reconstruction contained 124,397 particles, which had been extracted with a 768 × 768 pixel box and were downsampled to 256 × 256 pixels with a pixel sampling of 1.08 Å/pixel, resulting in a Nyquist resolution of 2.16 Å. The final map was sharpened in cryoSPARC as part of the local refinement procedure. A B-factor of-73.7 was applied to the map, which was calculated from the Guinier plot.

For atomic model generation, the coordinates of the SLC (5FFO)^25^ and TB2 domain of LTBP1 (1KSQ)^34^ were used as templates. First, the atomic coordinates were fitted to the cryo-EM map as rigid bodies using UCSF ChimeraX. Then, the coordinates were manually adjusted using Coot (0.9.8.7), followed by several rounds of global refinement using Real-space refinement in Phenix^42^. Rebuilding and real space refinement, including global minimization, and local grid search were performed against the sharpened map (B-factor of –73.7), but the final refinement of B-factors only was performed using the unsharpened map to ensure appropriate B-factor distributions. Validation of the model geometry was assessed using Molprobity 4.5.2^43^. and the wwPDB validation system^44^ and glycans were assessed using Privateer^45^. The model-map correlation indicates a resolution of 3.21 Å (FSC = 0.5) for the masked map (Supplementary Fig. 13).

### Force-probe molecular dynamics simulations

The SLC simulations used the crystal structure of integrin αVβ6 in complex with the SLC^25^ (PDB: 5FFO). Unresolved loops were built using MODELER^31^, with loops connecting the LAP arm and straitjacket domains built either ipsilaterally or contralaterally. The integrin β6 hybrid domain is not present in the SLC-integrin structure (PDB: 5FFO), so this domain was merged in using PDB:4UM8^32^ as a template. The common domains in the β6 chain of 4UM8 were aligned to 5FFO (chains E F) (RMS 1.59) using the Phenix structural alignment tool^46^, and the β6 hybrid domain was merged into the model using junctions at residues 131–132 and 350–351 (numbering from P18564 · ITB6_HUMAN). To facilitate comparison with previous literature, the representative pulling velocity reported by Dong et al. (*v* = 0.05 nm/ns) was adopted for SMD simulations^25^. The pulling force was applied to the β6 hybrid domain by distributing it evenly across all atoms within the domain, rather than applying the force to individual atoms as in Dong et al.^25^. This strategy is intended to minimize artifacts such as partial unfolding and undesired distortion of the tertiary structure (Supplementary Fig. 10).

The LLC simulations were performed in a similar manner, with the cryo-EM structure of the LLC replacing that of the SLC. In the LLC setup, the pulling force was counterbalanced by anchoring the COM of the LTBP1 TB2 domain. The choice to anchor the COM, rather than individual atoms (e.g., terminal Cα atoms), is motivated by the unresolved upstream and downstream regions of the TB2 domain, as well as uncertainty in the direction of the resistant force, as LTBP1 binds to extracellular matrix proteins, fibronectin^17^ and fibrillin^16,38^ via N- or C-terminal regions, respectively. The protein was solvated with TIP3P water molecules in a ~ 50 × 16 × 16 nm³ rectangular box containing ~1,260,000 atoms with counter ions neutralizing the system. Simulations were conducted with GROMACS 2024^47^ using the CHARMM36^48^ force field. Long-range electrostatic interactions were calculated using the Particle Mesh Ewald (PME) method^49^ with a grid spacing of 0.16 nm, while short-range electrostatic and van der Waals interactions were treated with a cutoff distance of 1.0 nm. Prior to steered molecular dynamics (SMD) simulations, the systems underwent energy minimization using the steepest descent algorithm, followed by a 40 ps NVT ensemble simulation with all heavy atoms restrained to relax the solvent surrounding the protein. Subsequently, a 1 ns NPT ensemble simulation was conducted to equilibrate the system temperature and pressure without any atomic restraints. The root mean square deviation (RMSD) of protein domains was analyzed individually to exclude large-scale domain motions from the RMSD calculation. During the 1 ns NPT simulation, all systems converged in temperature, pressure, and density, and the protein structures relaxed (Supplementary Figs. 14–19). For all simulations, the V-rescale thermostat (time constant 0.1 ps) and the C-rescale barostat (time constant 0.1 ps) were applied, with separate temperature coupling for protein and solvent groups to improve accuracy. After NVT and NPT equilibration, harmonic springs (500 kJ/mol/nm) were attached to the COM of the β6 hybrid domain and moved away from the anchor point, namely, Cα atoms in LAP Cys33 residues in the SLC simulations or the COM of the LTBP1 TB2 domain in the LLC simulations. The anchor point was positionally restrained with spring constants of 1000 kJ/mol/nm² for the SLC simulation and 40000 kJ/mol/nm² for the LLC simulation. The choice of spring constant depends on the number of atoms in the restrained group. In the SLC system, the anchor consists of a single atom, whereas in the LLC system, the entire TB2 domain is restrained, which requires a larger spring constant to achieve effective anchoring. However, excessively large spring constants may introduce instability due to unrealistically high external forces. Therefore, the value of k = 40000 kJ/mol/nm² was determined through trial and error, ensuring that the TB2 domain is effectively restrained while maintaining overall simulation stability. Force events were defined as local maxima in the force–pulling distance curve. Conformational changes in the protein complex were characterized based on the interactions between the growth factor region and the arm domain, which includes the fastener and α2 helix. The system consists of two chains for both the growth factor and arm domains (denoted as chain C and chain D; see Supplementary Figs. 7, 9 and 11). To quantify these interactions, the buried surface area was calculated between the growth factor domain and each of the fastener and α2 helix regions from both chains, resulting in eight interaction pairs in total. A conformational event was defined as the loss of interaction between a given pair, operationally identified when the buried surface area decreased to zero. These events were then correlated with force peaks to establish the relationship between mechanical rupture and structural changes. Simulations were performed using the computational shared facility at The University of Manchester. Average force was calculated from the beginning of pulling until 30 nm pulling distance. Force events are detected by force-pulling curve and buried solvent accessible surface area (SASA) between force-resisting elements on LAP and growth factor (Supplementary Figs. 7, 9, 11).

### Statistics and reproducibility

Statistical significance for the TGFβ Bioassays comparing WT, F1387A and W330A LLC proteins was determined by two-way ANOVA with Tukey’s multiple comparisons test. Three independent biological repeats were conducted, where the value for each biological repeat was determined from the average of three technical replicates. No data were excluded from the analyses. Sample size is based on previous experience in detecting significant differences. The Investigators were not blinded during experiments and outcome assessment, as all measurements are performed in a non-biased manner using an automated plate reader.

### Reporting summary

Further information on research design is available in the Nature Portfolio Reporting Summary linked to this article.

## Supplementary information

Supplementary Information

Reporting Summary

Transparent Peer Review file

## Source data

Source Data

## Data Availability

The structure and cryo-EM data generated in this study have been deposited in the EMDB and PDB databanks under accession codes PDB ID 9R3S 10.2210/pdb9r3s/pdb; EMD-53558. The input simulation files underlying Figs. 5, 6 and Supplementary Figs. 6–9, 11 and 14-19 are available on figshare 10.6084/m9.figshare.31231798. The source data underlying Figs. 3B–E, 4C, D, and Supplementary Figs. 1, 4 and 5 are provided as a Source Data file. All data supporting the results of this study can be found in the article, supplementary, on figshare and the source data file. Source data are provided with this paper.
