# Supplementary material for: Structural basis for the contribution of latent TGFβ binding protein to TGFβ latency and activation
Source: Nat Commun. 2026 Aug 5;17:7619. doi: 10.1038/s41467-026-75834-8 (PMC13443091; doi:10.1038/s41467-026-75834-8)
Supplement: Supplementary file 1 [file 41467_2026_75834_MOESM1_ESM.pdf]

Supplementary Figure 1

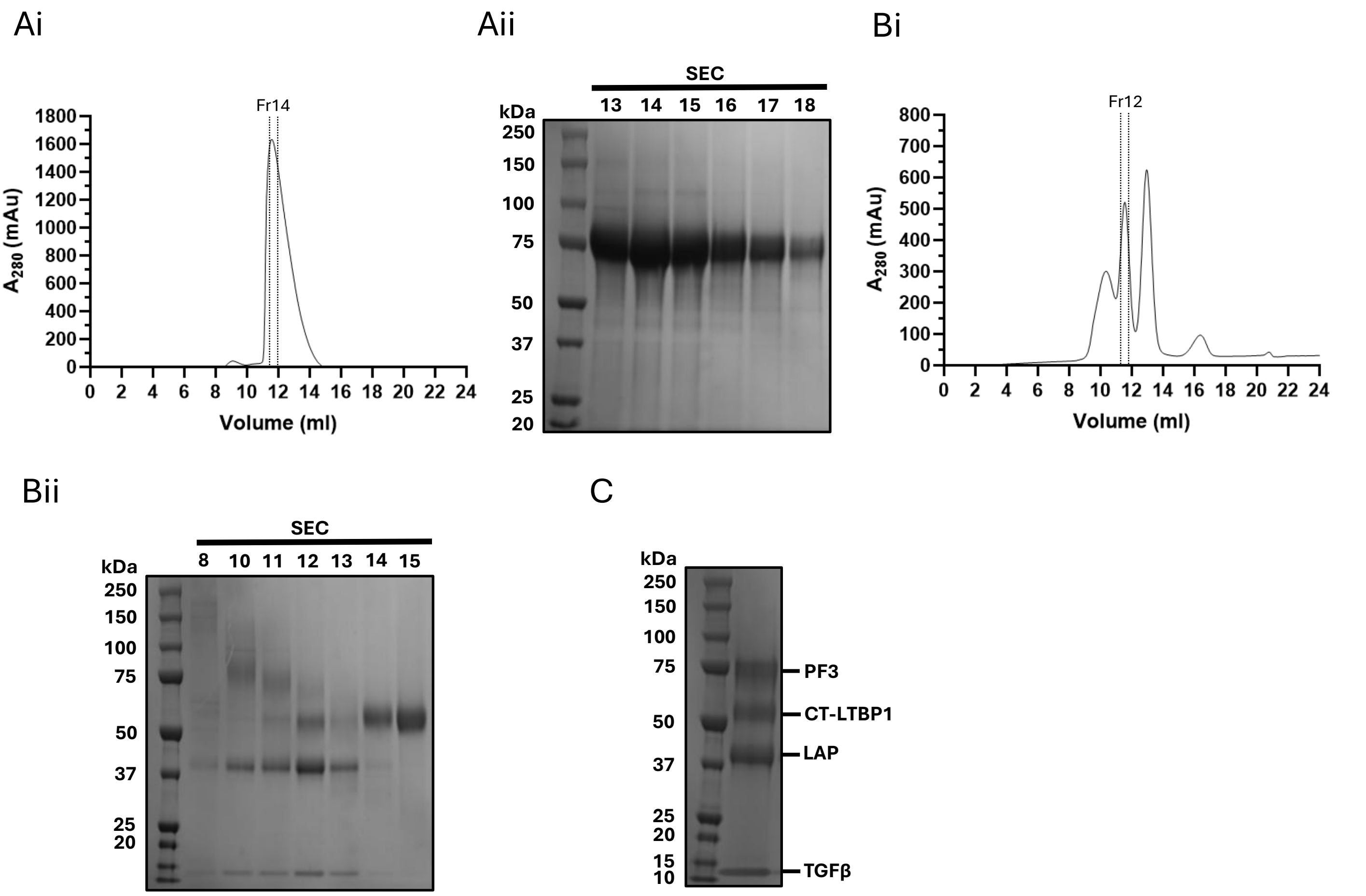

Supplementary Figure 1. Purification of LLC with fibrillin-1. (A) Protein purification of the N-terminal fibrillin-1 region (PF3 residues 1-722)[39]. (i) Size exclusion chromatography (SEC) using an S200i column. Chromatogram shows the elution volume (ml) plotted against absorbance measured at 280 nm (mAu) with PF3 eluting at 11-14ml. Fr: fraction. (ii) 4-12% SDS-PAGE analysis following SEC purification, fraction 14 was used for complexing with LLC. All samples are reduced. (B) Protein purification of the LLC. (i) SEC using an S200i column. Chromatogram shows the elution volume (ml) plotted against absorbance measured at 280 nm (mAu) with LLC eluting at 11.5-12ml. Fr: fraction. (ii) 4-12% SDS-PAGE analysis following SEC purification, fraction 12 was used for complexing with PF3. All samples are reduced. SEC: size-exclusion chromatography fractions. (C) 4-12% SDS-PAGE analysis of LLC-PF3 complex used for cryo-EM. PF3 was mixed with LLC at a 4.2x molar excess of PF3 for 3 hours at room temperature. Affinity pulldown utilised the strep-tag on the LLC to remove excess PF3, the resulting complex was 1mg/ml. Sample is reduced. Representative purifications are shown, purifications were repeated at least three times with similar results.

Supplementary Figure 2

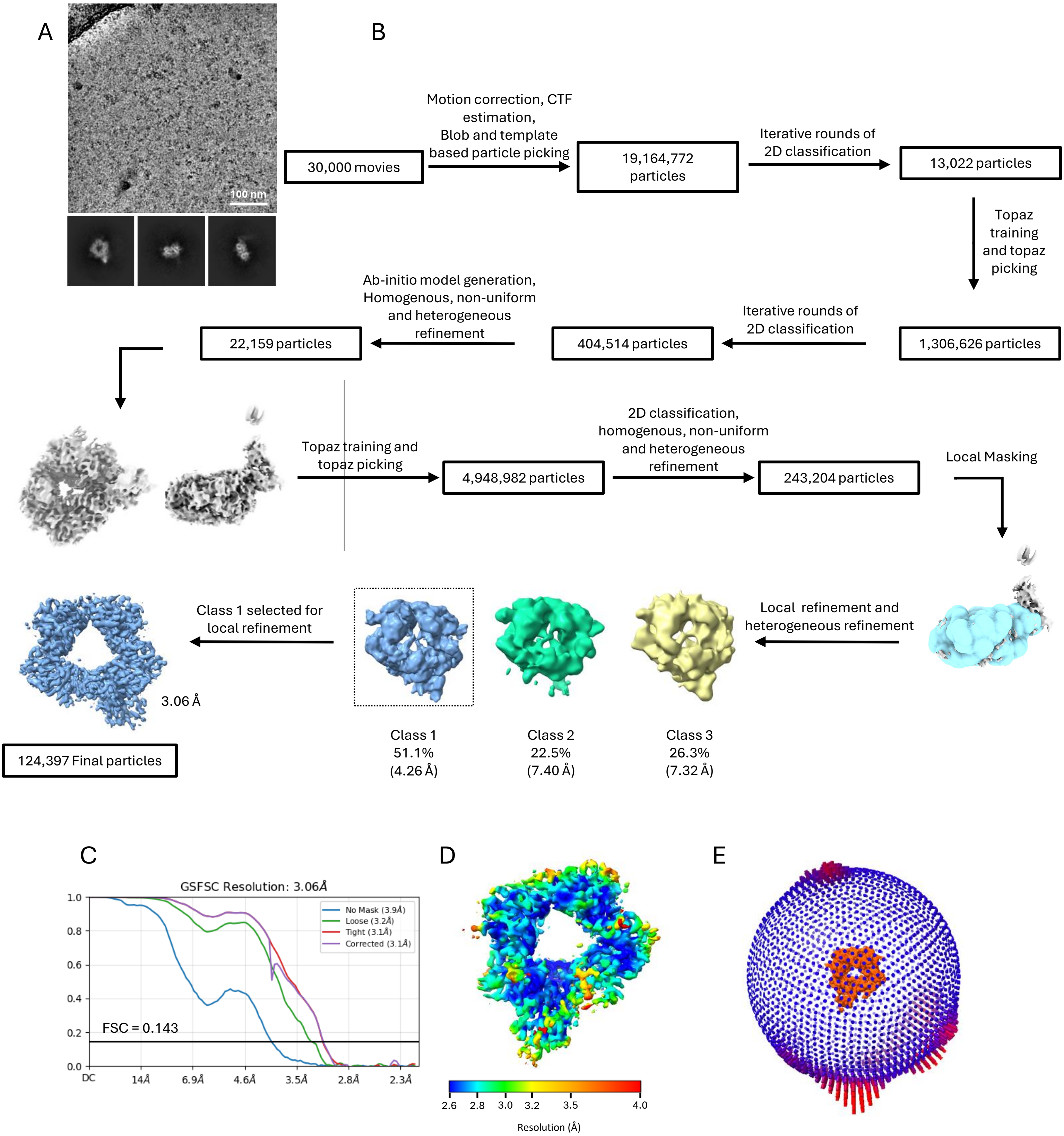

Supplementary Figure 2. Cryo-EM and data processing workflow of the LLC complex. (A) Representative micrograph from 30,000 movies of the LLC sample in vitreous ice (top) and 2D class averages (bottom). (B) Workflow depicting the cryo-EM data processing of the LLC complex. (C) Gold-standard Fourier shell correlation (GSFSC) curves for the 3D reconstruction of LLC. Fourier shell threshold = 0.143. (D) Local resolution estimation. Performed in cryoSPARC. (E) Euler angle distribution of the set of particles that contributed to the final density map of LLC from homogenous refinement in cryoSPARC.

Supplementary Figure 3

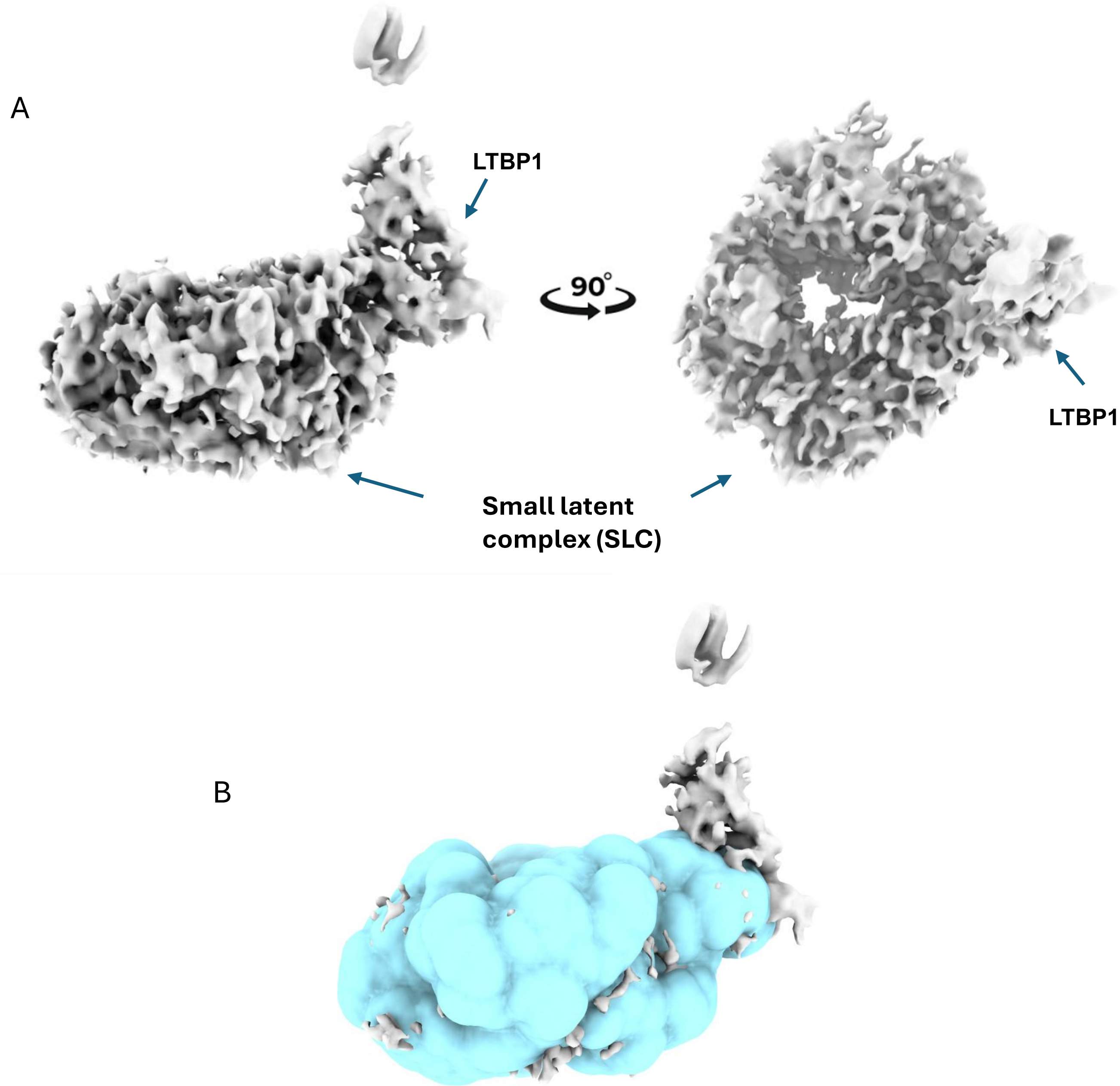

Supplementary Figure 3. Cryo-EM reconstruction of LLC complex showing extra LTBP1 density. (A) Orthogonal views of an auto masked non-uniform refined cryoEM map of the LLC complex, when shown at a lower threshold value of 0.07, diffuse density corresponding to LTBP1 can be seen. (B) A mask used in local refinement of the LLC complex, is shown in blue.

Supplementary Figure 4

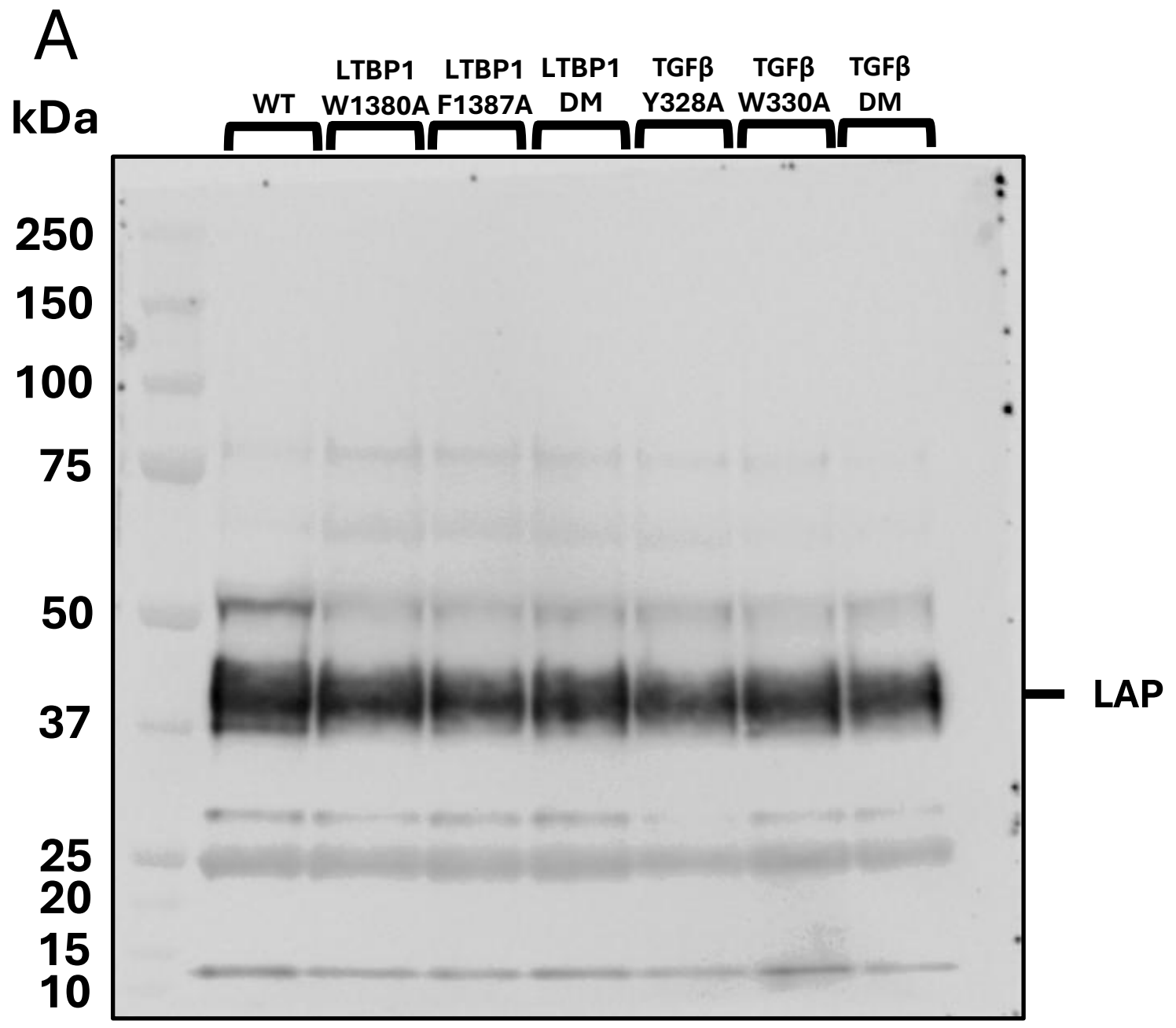

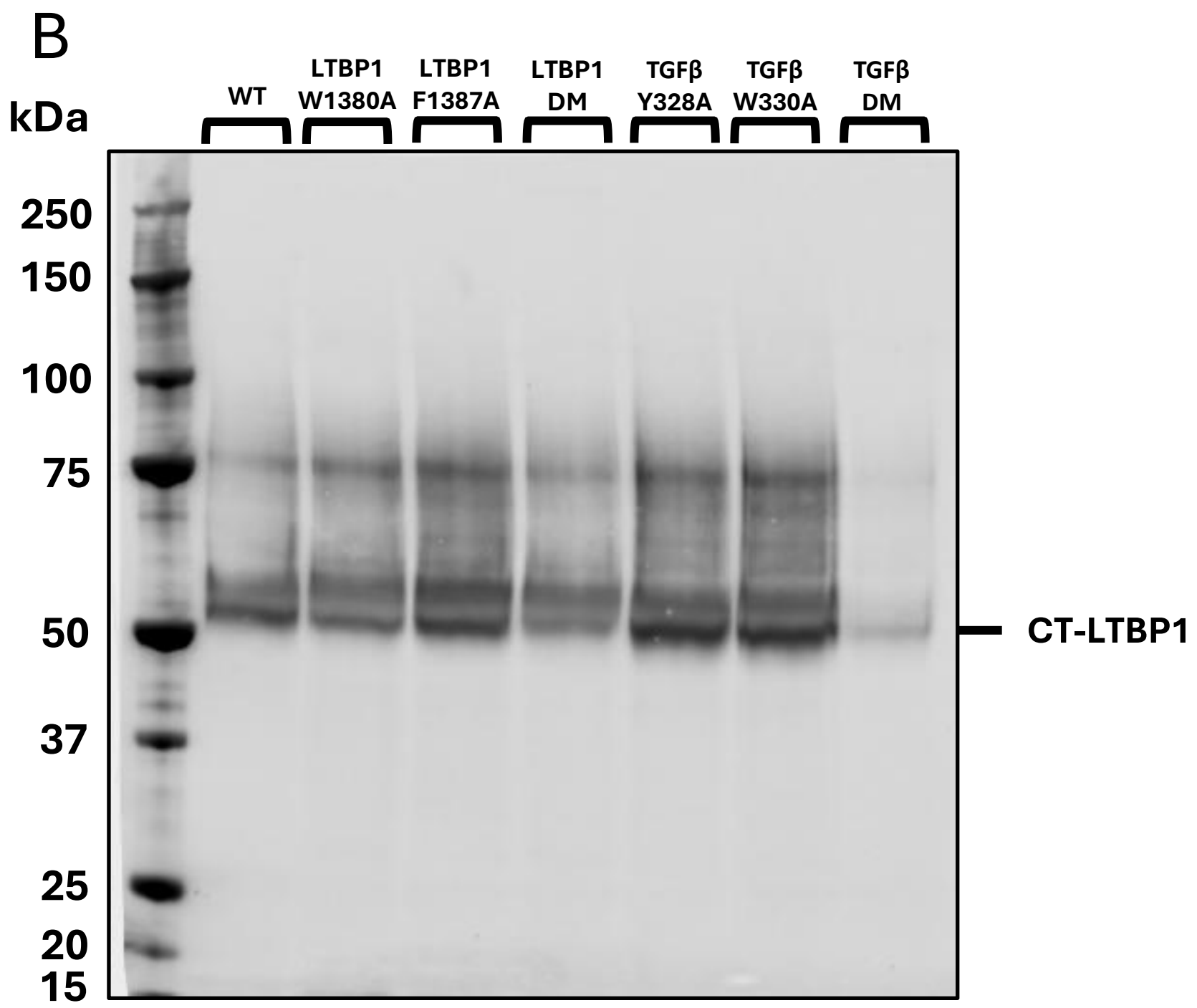

Supplementary Figure 4. Western blot analysis of expression media from WT and mutant LLC cell lines probing for LAP and LTBP1. 4-12% SDS-PAGE Western blot analysis of expression media from WT and mutant LLC cell lines. Equal total protein was loaded as determined by BCA assay. All samples are reduced. **(A)** FLAG-tagged LAP was detected using an anti-FLAG antibody. **(B)** Strep-tagged LTBP1 was detected with an anti-Strep antibody. Representative blots are shown (n = 3).

Supplementary Figure 5

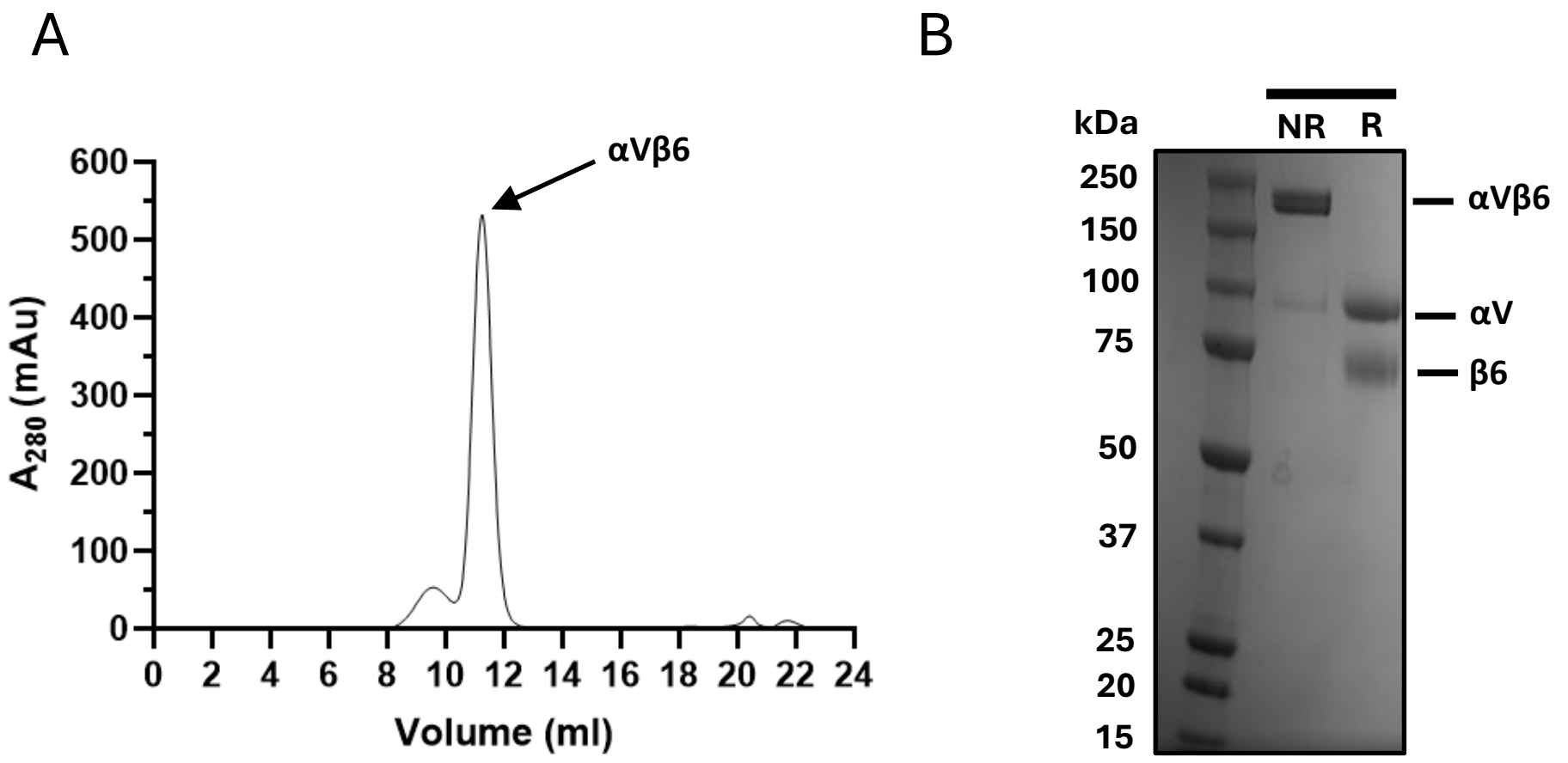

Supplementary Figure 5. Protein purification of αVβ6 headpiece. (A) SEC of αVβ6 using an S200i column. Chromatogram shows the elution volume (ml) plotted against absorbance measured at 280 nm (mAu) with αVβ6 eluting at 11-12ml. **(B)** 4-12% SDS-PAGE analysis of the purification of αVβ6 following Strep-Tactin XT resin purification. R: reduced, NR: non-reduced, SEC: size-exclusion chromatography fractions. Representative purification shown, purifications were repeated at least three times with similar results.

Supplementary Figure 6

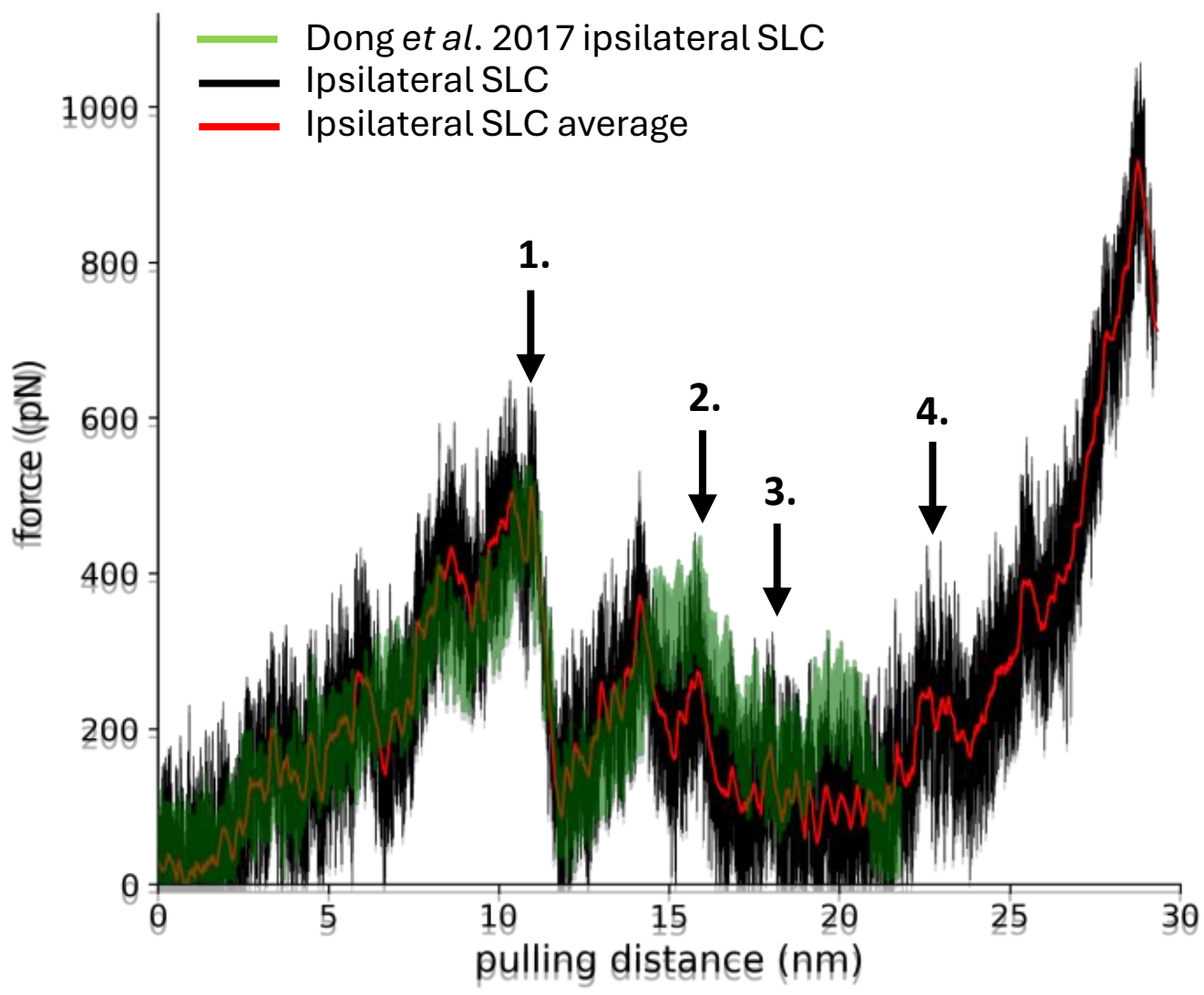

Key:

1. Integrin-bound LAP fastener unsnapping

2. Other LAP fastener unsnapping

3. Integrin-bound LAP α2 helix unwinding

4. Other LAP α2 helix unwinding

Supplementary Figure 6. Comparison of force-probe molecular dynamic simulations of ipsilateral SLC. Force displacement curves show pulling distance (nm) plotted against force (pN). Green: ipsilateral SLC analysis from Dong *et al*. 2017[25], black: replicated ipsilateral SLC analysis (shown in Figure 5A), red: ipsilateral SLC average.

Supplementary Figure 7

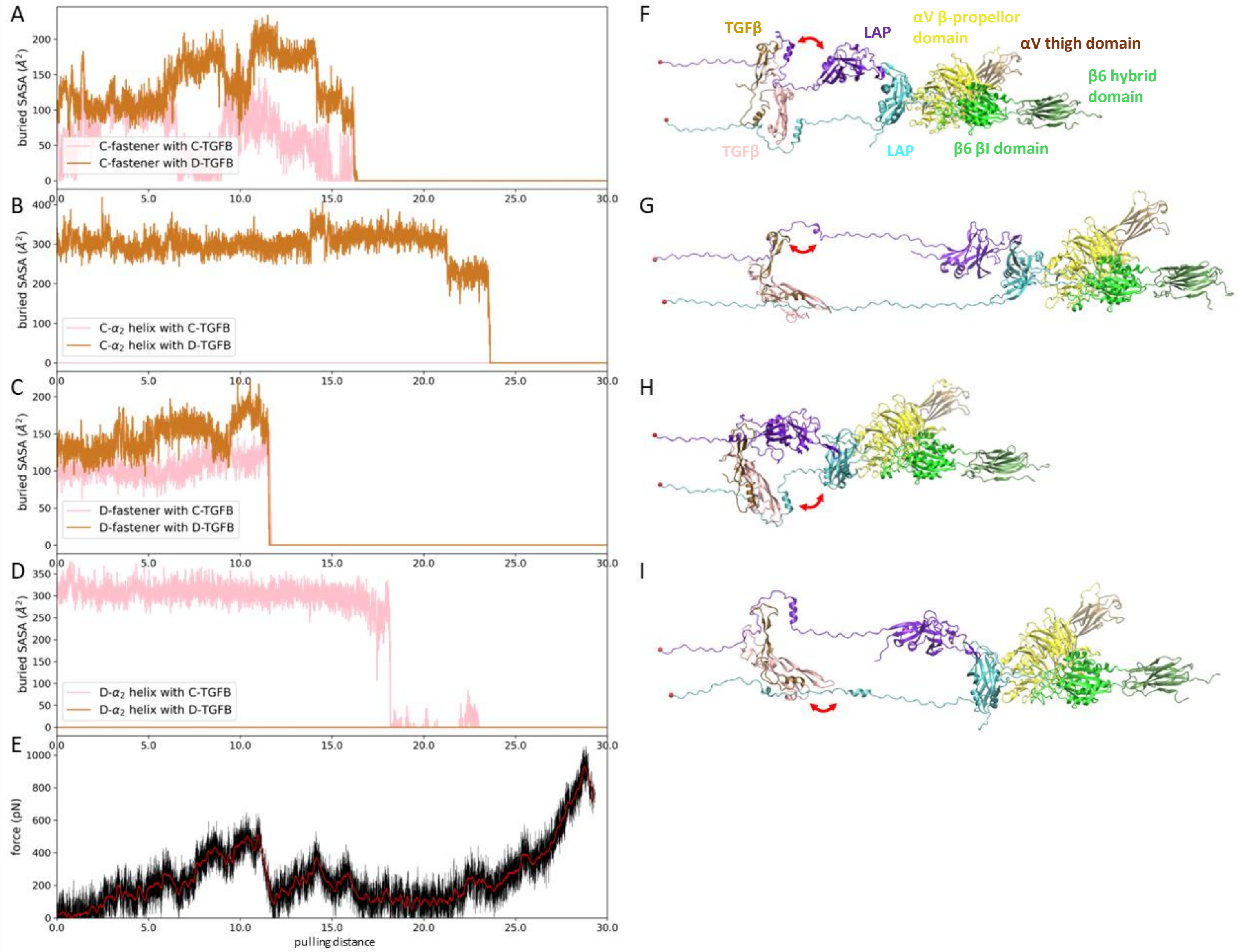

Supplementary Figure 7. Buried SASA between LAP fastener/α2 helices and the mature growth factor (GF) in the ipsilateral conformation. Domain names and corresponding colours are as follows: lilac and cyan LAP domains (chains C and D, respectively); pink and beige mature GF (chains C and D, respectively). The integrin is bound to the RGD region on chain D. Chain IDs reflect those in PDB ID:5PPO[25]. **(A)** Buried SASA between fastener (chain C) and the mature GF. **(B)** Buried SASA between α2 helices (chain C) and the mature GF. **(C)** Buried SASA between the fastener (chain D) and the mature GF **(D).** Buried SASA between α2 helices (chain D) and the mature GF. **(E)** The force-distance curve of pulling. **(F)** Snapping of the fastener (chain C) from the mature GF. **(G)** Unbinding of α2 helices (chain C) from the mature GF. **(H)** Snapping of fastener (chain D) from the mature GF. **(I)** Unbinding of α2 helices (chain D) from the mature GF.

Supplementary Figure 8

A B

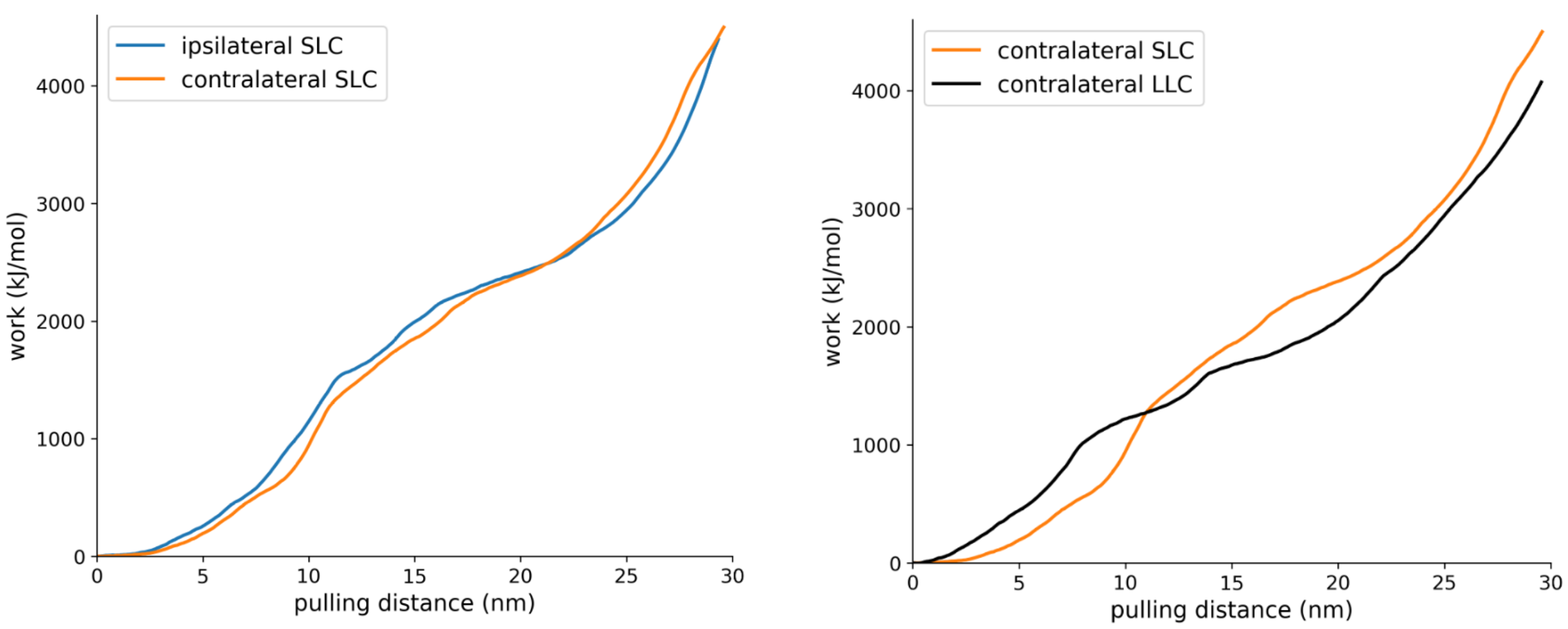

Supplementary Figure 8. Total energy required to release TGFβ in integrin-mediated molecular dynamic simulations. (A-B) Total energy curves with pulling distance (nm) plotted against work (KJ/mol) comparing total energy required for TGFβ release in **(A)** Ipsilateral vs contralateral SLC. Ipsilateral: blue, contralateral: orange. **(B)** SLC vs LLC. SLC: orange, LLC: black.

Supplementary Figure 9

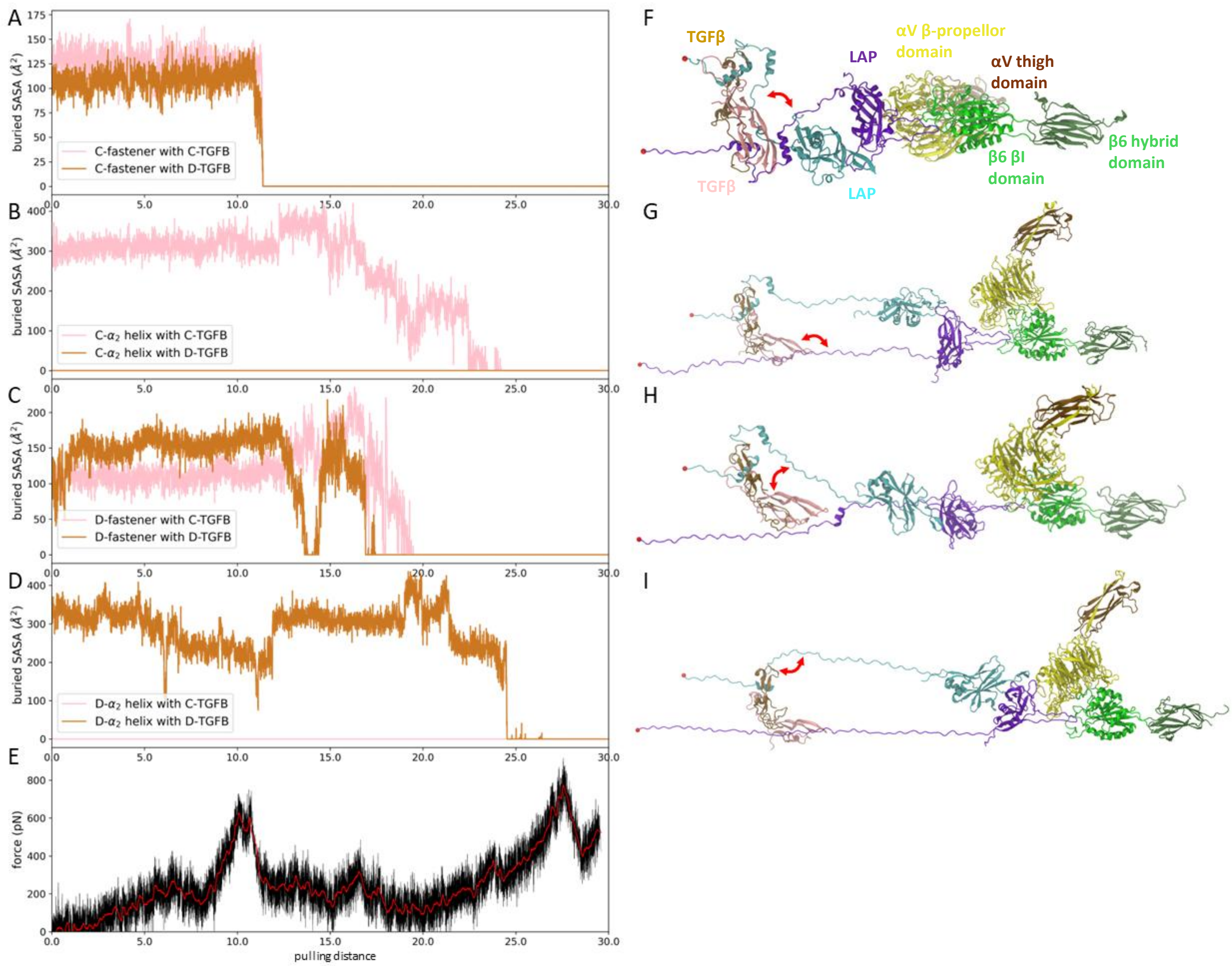

Supplementary Figure 9. Buried SASA between LAP fastener/α2 helices and the mature growth factor (GF) for the contralateral conformation. Domain names and corresponding colours are as follows: lilac and cyan LAP domains (chains C and D, respectively); pink and beige mature GF (chains C and D, respectively). The integrin is bound to the RGD region on chain C. Chain IDs reflect those in PDB ID:5PPO[25]. **(A)** Buried SASA between fastener (chain C) and the mature GF. **(B)** Buried SASA between α2 helices (chain C) and the mature GF. **(C)** Buried SASA between the fastener (chain D) and the mature GF **(D).** Buried SASA between α2 helices (chain D) and the mature GF. **(E)** The force-distance curve of pulling. **(F)** Snapping of the fastener (chain C) from the mature GF. **(G)** Unbinding of α2 helices (chain C) from the mature GF. **(H)** Snapping of fastener (chain D) from the mature GF. **(I)** Unbinding of α2 helices (chain D) from the mature GF.

Supplementary Figure 10

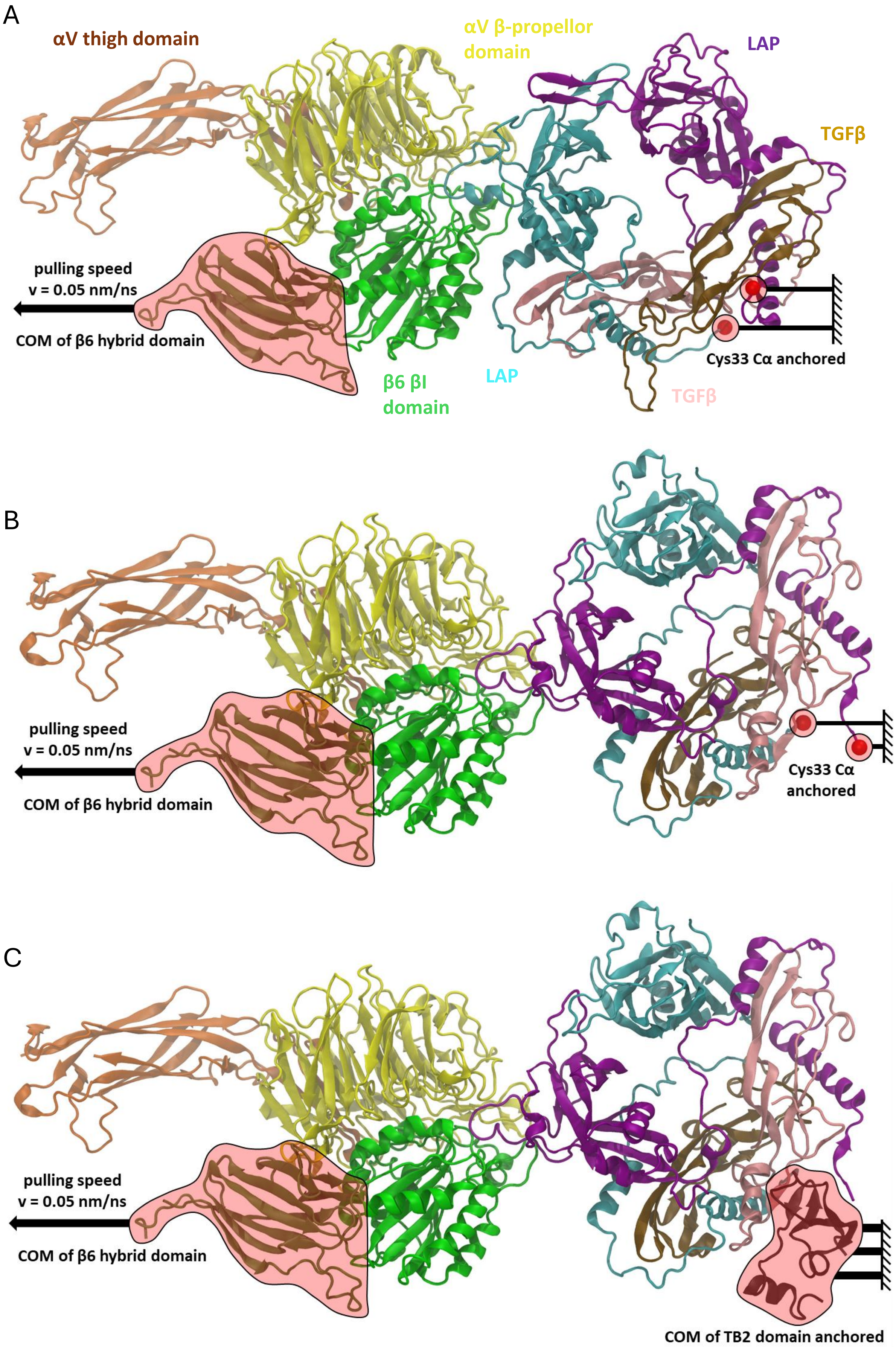

Supplementary Figure 10. Ipsilateral and contralateral models and corresponding pulling/anchored groups. The red-shaded region on the left represents the β6 hybrid domain, which is pulled at a speed of 0.05 nm/ns. In (A) and (B), the red-shaded regions on the right correspond the Cα atoms of the two Cys33 residues of LAP, which are kept fixed during the pulling process with LAP in either an ipsilateral (A) or contralateral (B) arrangement. In (C), the centre of mass (COM) of the TB2 domain of LTBP1 is kept fixed during the pulling process with LAP in the contralateral arrangement.

Supplementary Figure 11

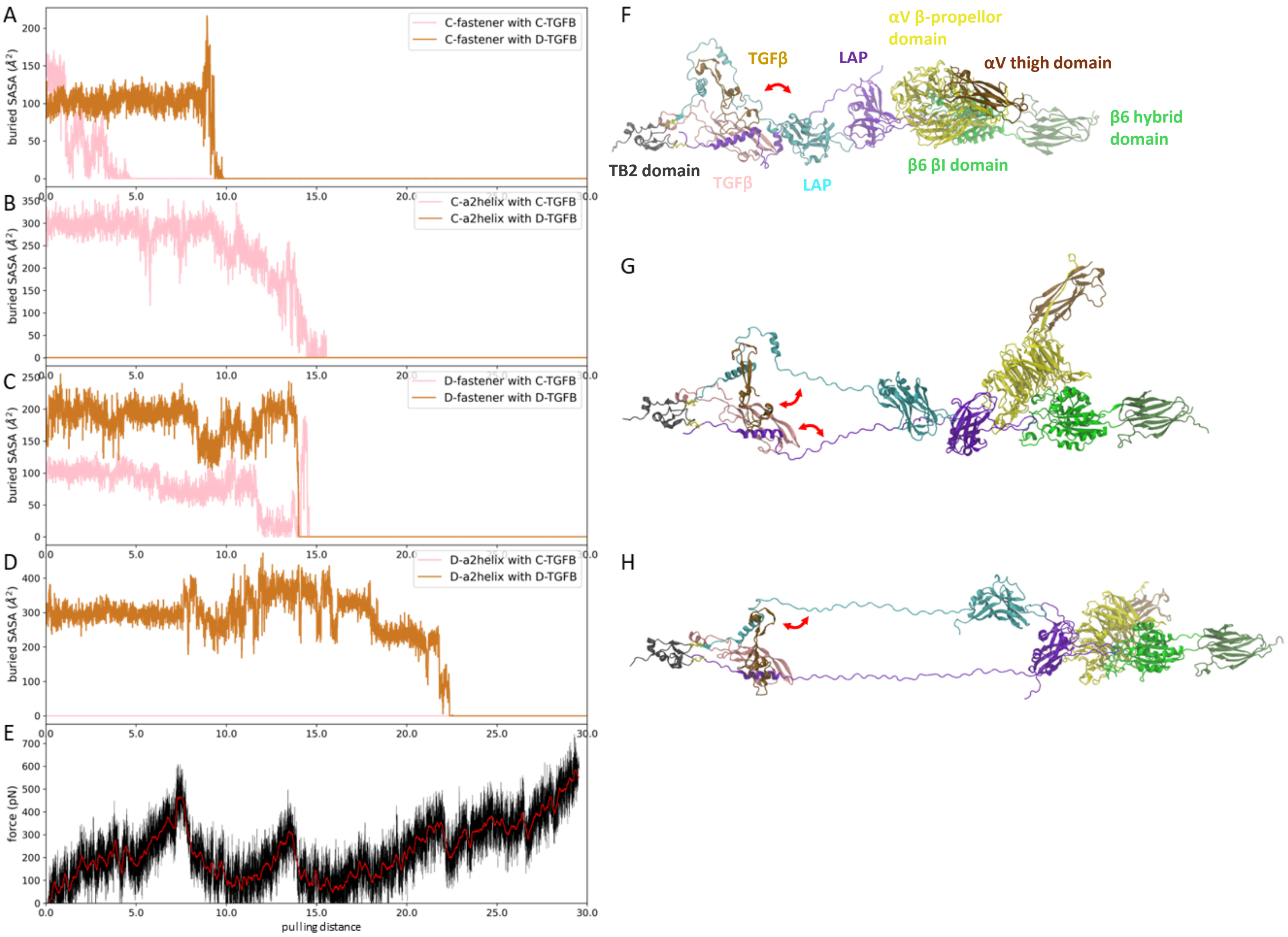

Supplementary Figure 11. Buried SASA between LAP fastener/α2 helices and the mature growth factor (GF) for the LTBP1-LLC contralateral conformation. Domain names and corresponding colours are as follows: lilac and cyan LAP domains (chains C and D, respectively); pink and beige mature GF (chains C and D, respectively); grey LTBP1 TB2 domain. The integrin is bound to the RGD region on chain C. **(A)** Buried SASA between fastener (chain C) and the mature GF. **(B)** Buried SASA between α2 helices (chain C) and the mature GF. **(C)** Buried SASA between the fastener (chain D) and the mature GF. **(D)** Buried SASA between α2 helices (chain D) and the mature GF. **(E)** The force-distance curve of pulling. **(F)** Snapping of the fastener (chain C) from the mature GF. **(G)** Snapping of fastener (chain D) and unbinding of α2 helices (chain C) from the mature GF. **(H)** Unbinding of α2 helices (chain D) from the mature GF.

Supplementary Figure 12

A

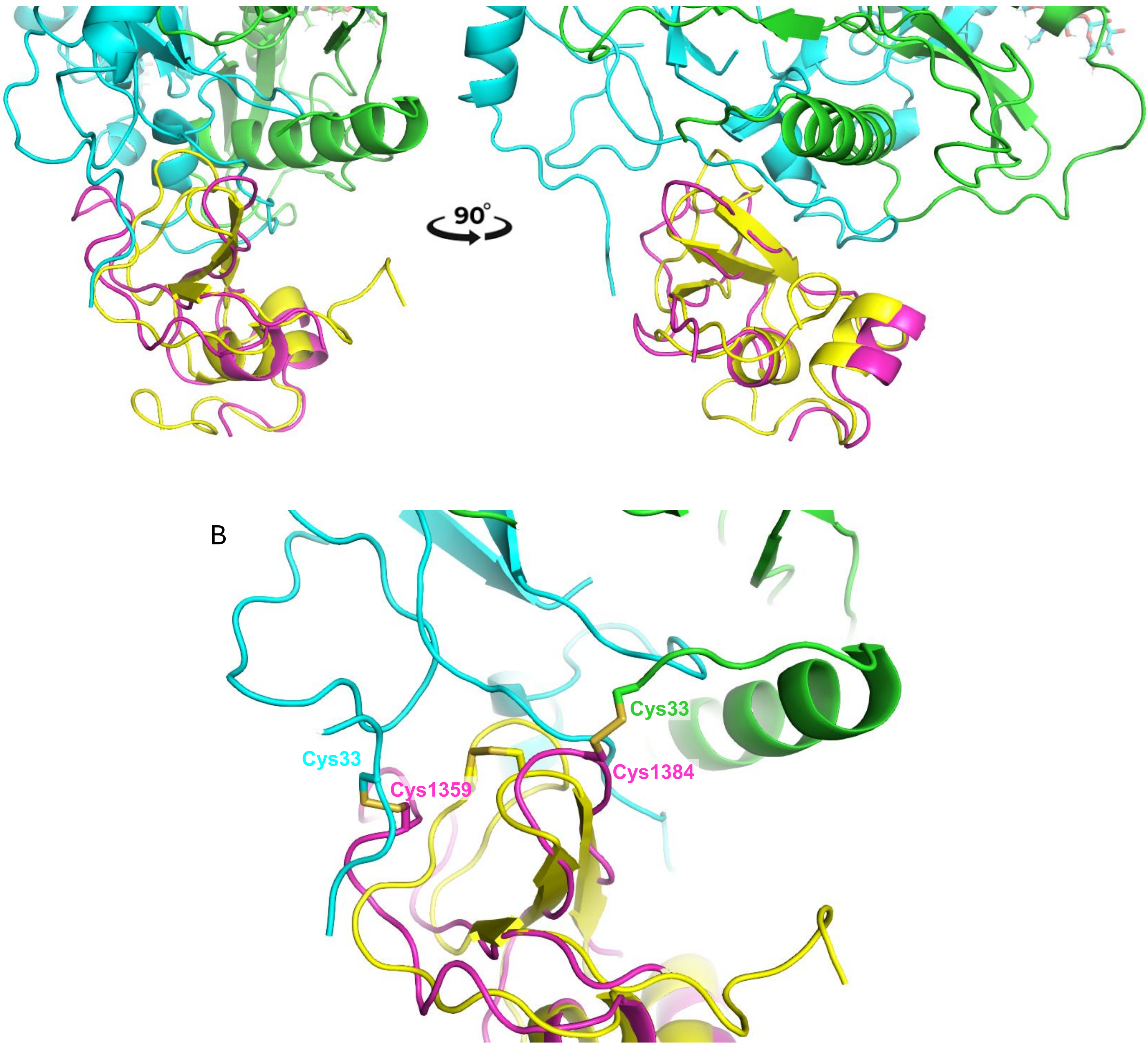

Supplementary Figure 12. Structural rearrangement of the LTBP1 TB2 domain in the latent complex compared to the structure of the isolated TB2 domain. (A) Ribbon representation of the atomic model of the LLC shown in two orthogonal orientations superimposed with the NMR structure of the isolated LTBP1 TB2 domain (PDB ID:1KSQ)[34]. The two molecules of the SLC dimer are coloured green (chain A) and cyan (chain B), and the TB2 domain of LTBP1 in the latent complex is coloured magenta and yellow for the isolated TB2 domain. **(B)** Residues Cys33 of LAP are disulphide bonded to Cys1359 and Cys1384 in the TB2 domain in the latent complex, whereas in the isolated TB2 domain they are involved in an intramolecular disulphide bond.

Supplementary Figure 13

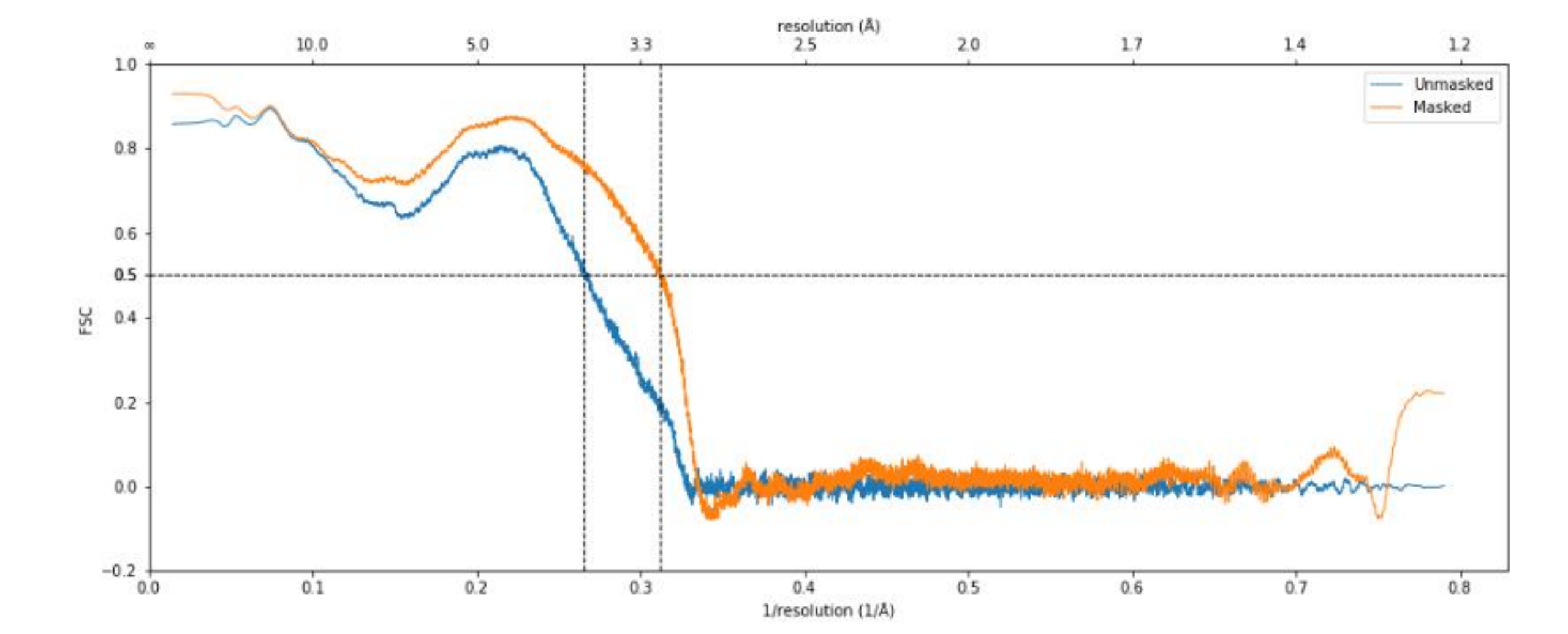

Supplementary Figure 13. Graph plotting the FSC for the model-map correlation with and without masking. The FSC for the model-map correlation with (orange) and without masking (blue). The intersections of the curves with FSC=0.5 are shown. The resolution of the masked map is 3.21Å at FSC=0.5.

Supplementary Figure 14

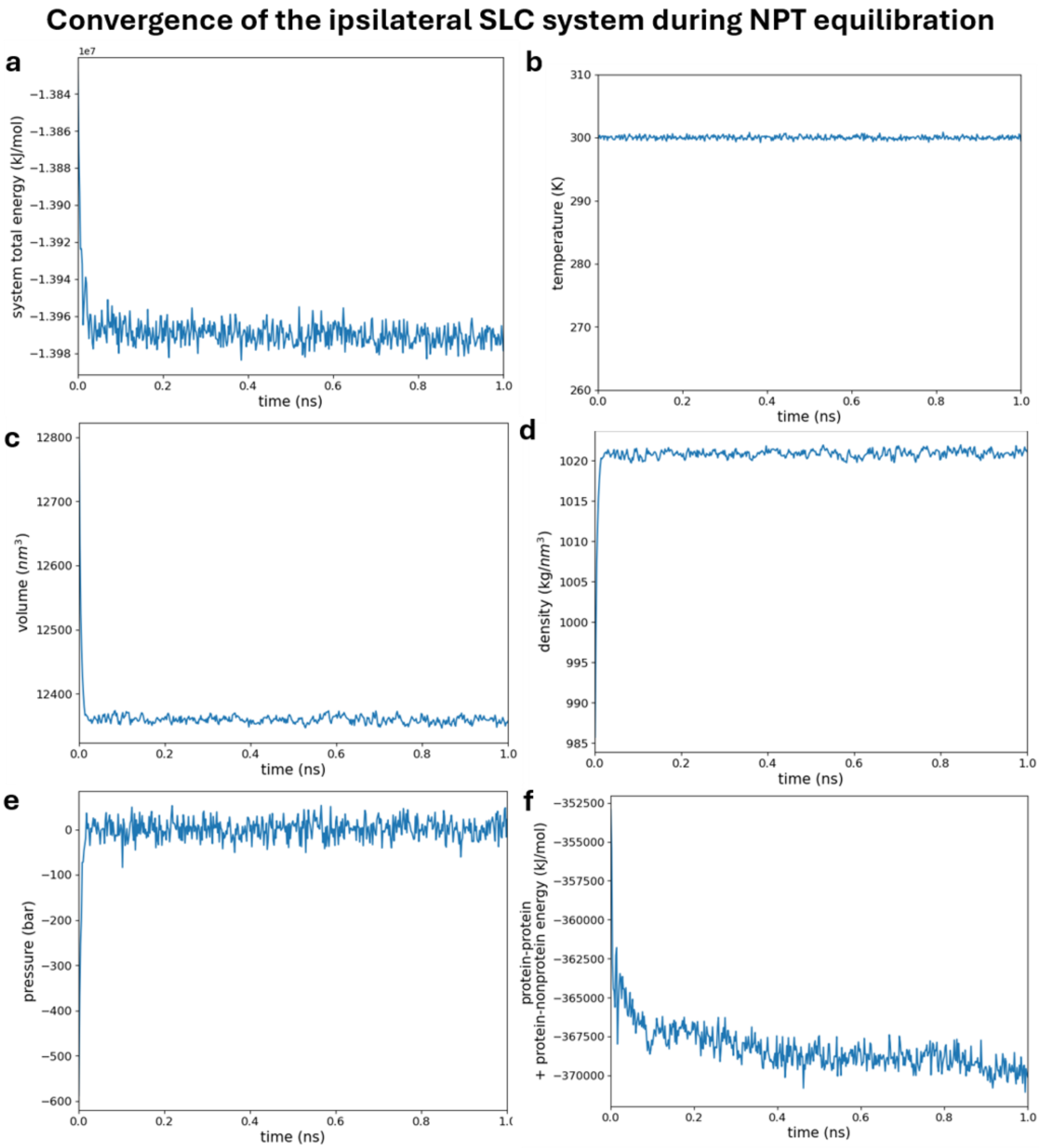

Supplementary Figure 14. Convergence of the ipsilateral SLC system during NPT equilibration. (a) Total energy, (b) temperature, (c) volume, (d) density, and (e) pressure of the ipsilateral SLC system during 1ns NPT process. (f) Total protein-related energy, defined as the sum of protein–protein and protein–nonprotein (water and ions) interaction energies.

Supplementary Figure 15

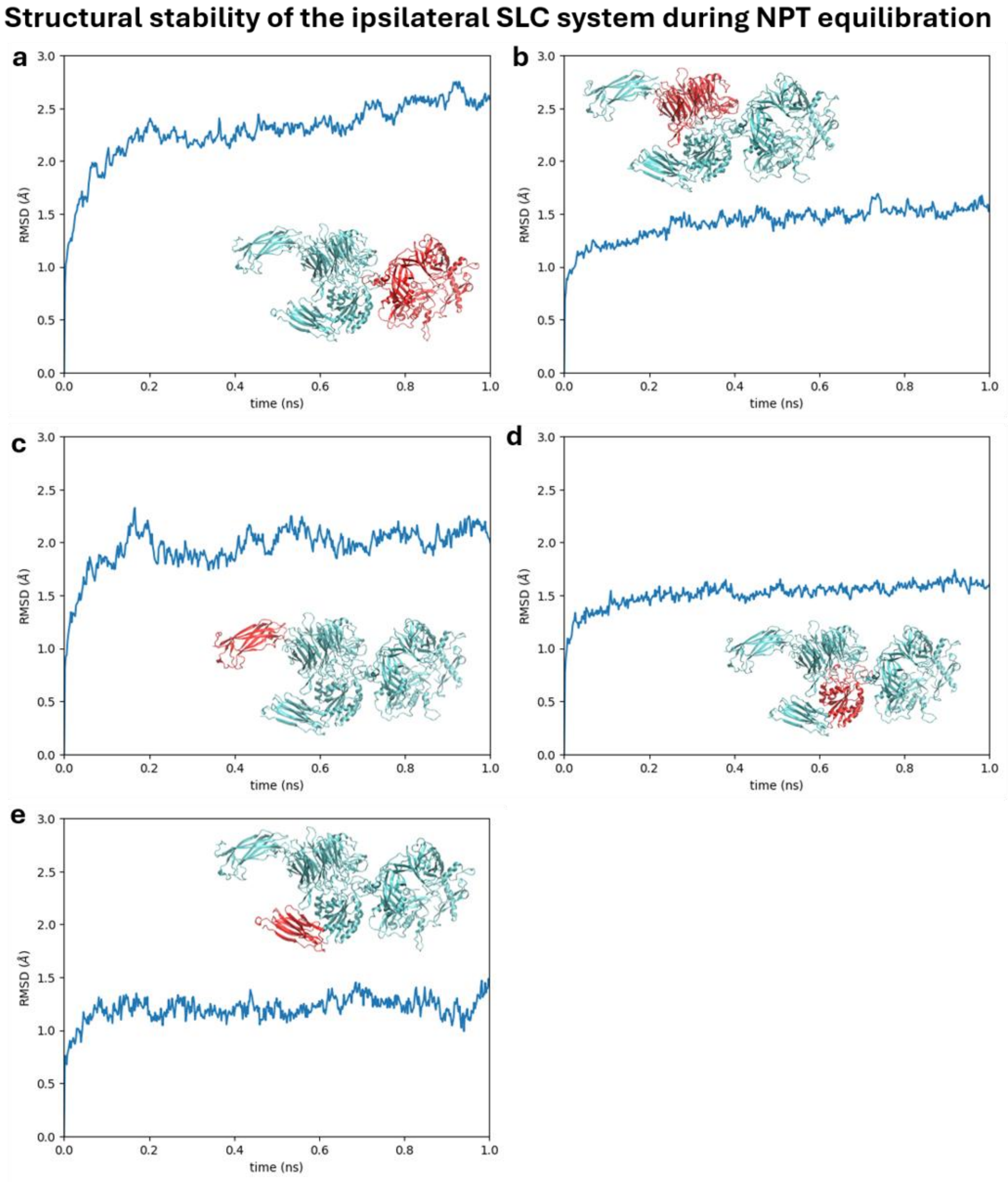

Supplementary Figure 15. Structural stability of the ipsilateral SLC system during NPT equilibration. Root mean square deviation (RMSD) of individual domains in the ipsilateral SLC system during 1ns NPT process. The red region in the structural snapshot indicates the analyzed domain. (a) LAP + GF + TB2, (b) β-propeller, (c) αv, (d) βI, and (e) β6.

Supplementary Figure 16

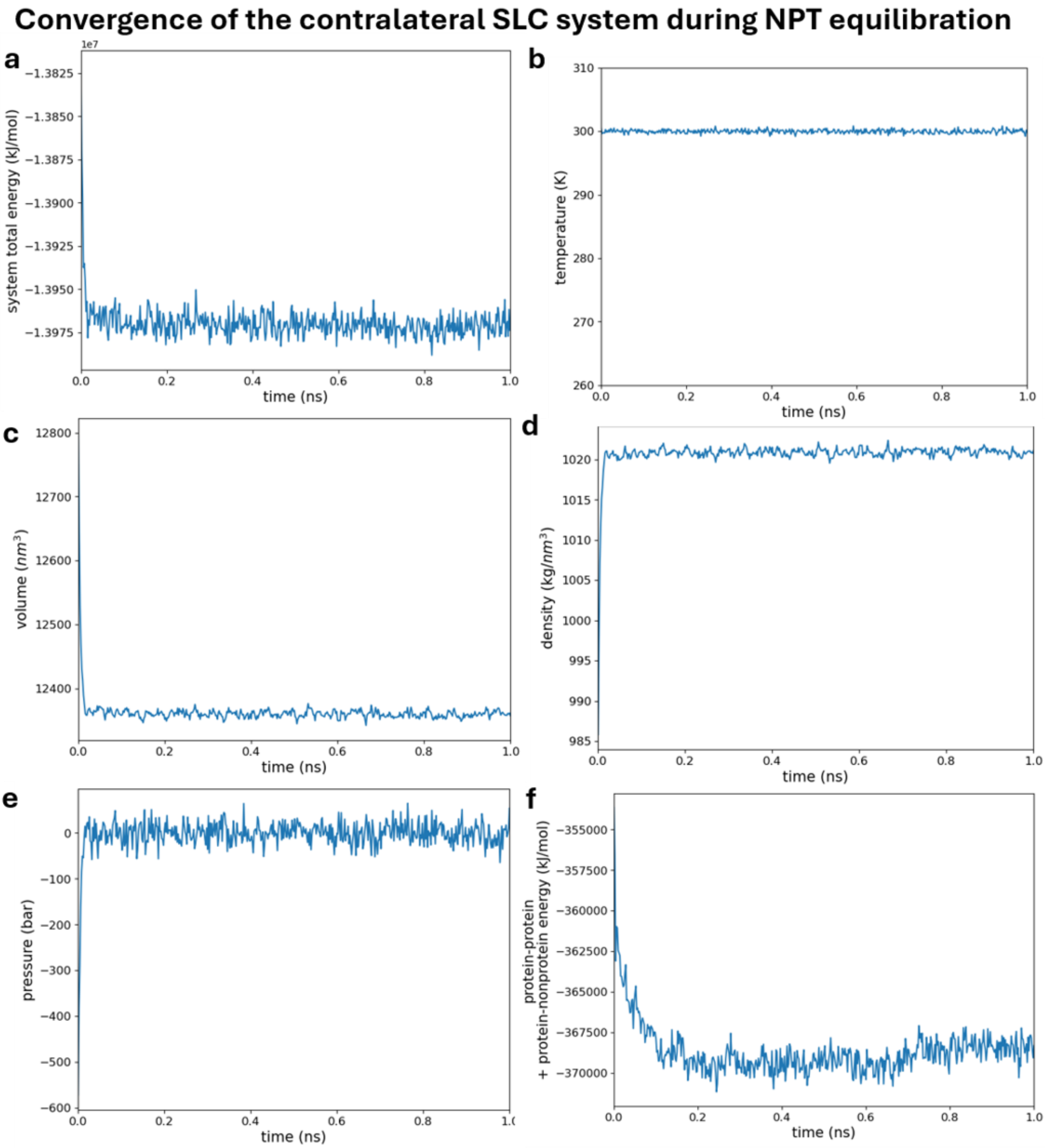

Supplementary Figure 16. Convergence of the contralateral SLC system during NPT equilibration. (a) Total energy, (b) temperature, (c) volume, (d) density, and (e) pressure of the contralateral SLC system during 1ns NPT process. (f) Total protein-related energy, defined as the sum of protein–protein and protein–nonprotein (water and ions) interaction energies.

Supplementary Figure 17

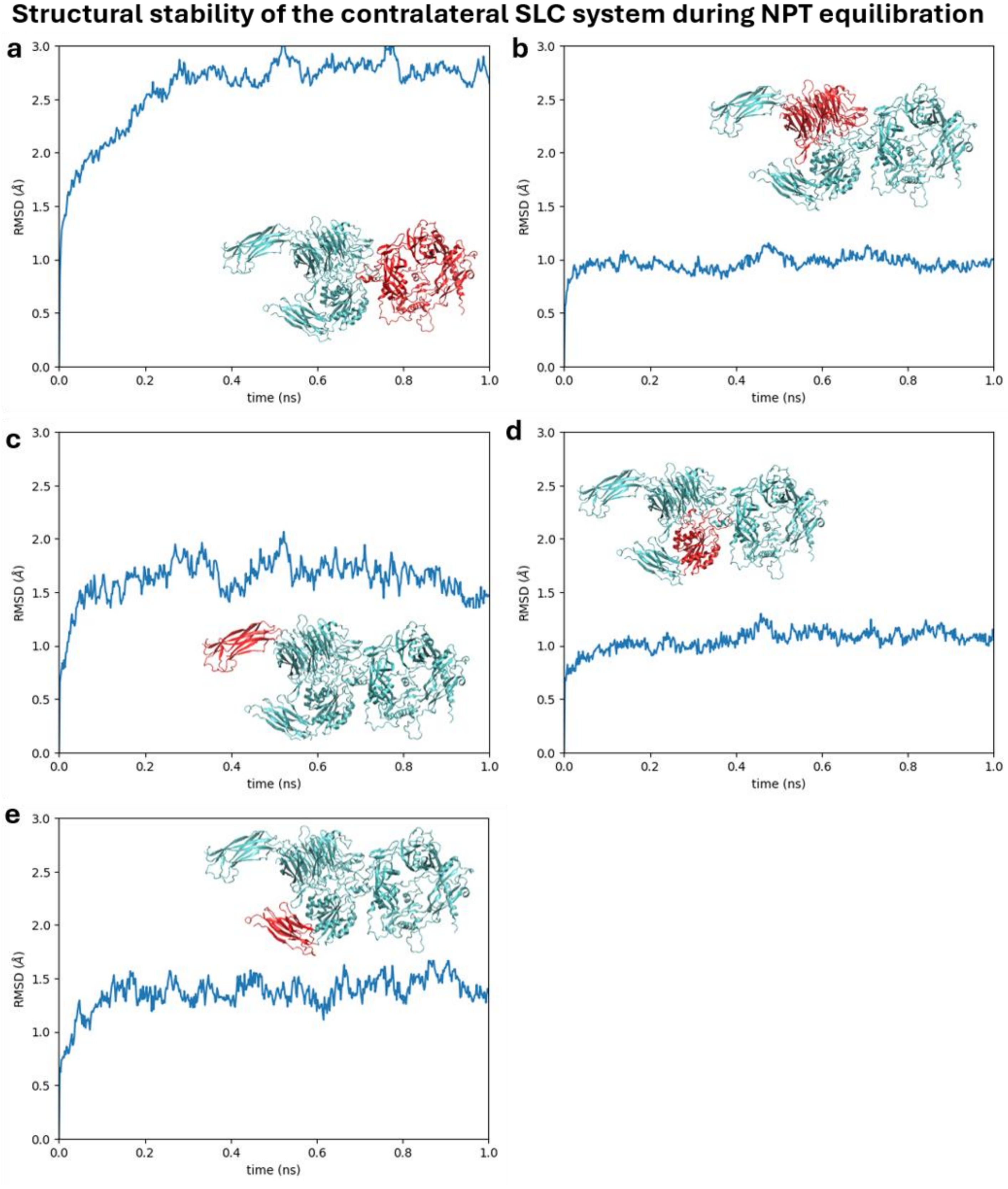

Supplementary Figure 17. Structural stability of the contralateral SLC system during NPT equilibration. Root mean square deviation (RMSD) of individual domains in the contralateral SLC system during 1ns NPT process. The red region in the structural snapshot indicates the analyzed domain. (a) LAP + GF + TB2, (b) β-propeller, (c) αv, (d) βI, and (e) β6.

Supplementary Figure 18

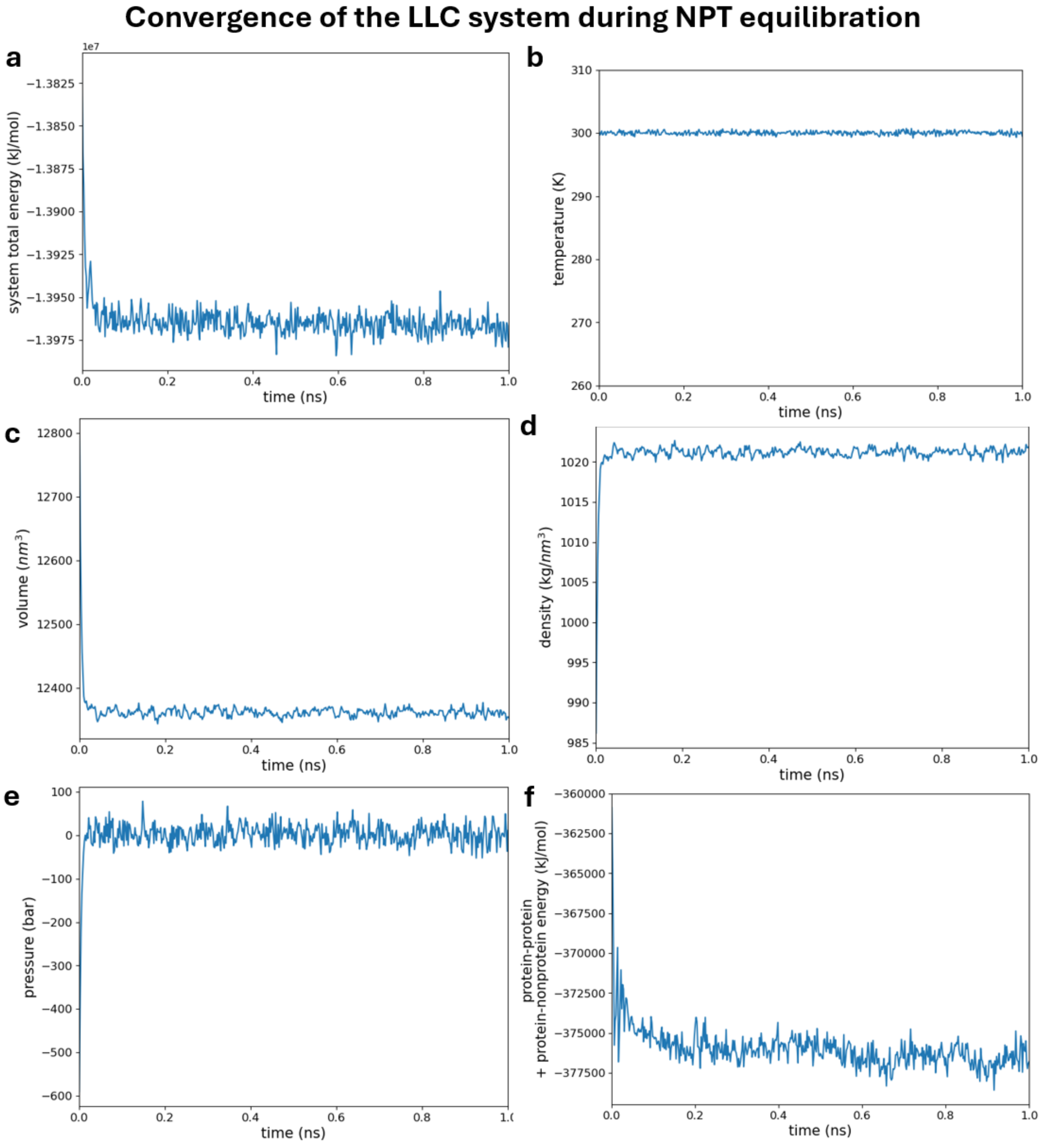

Supplementary Figure 18. Convergence of the LLC system during NPT equilibration. (a) Total energy, (b) temperature, (c) volume, (d) density, and (e) pressure of the system during 1ns NPT process. (f) Total protein-related energy, defined as the sum of protein–protein and protein–nonprotein (water and ions) interaction energies.

Supplementary Figure 19

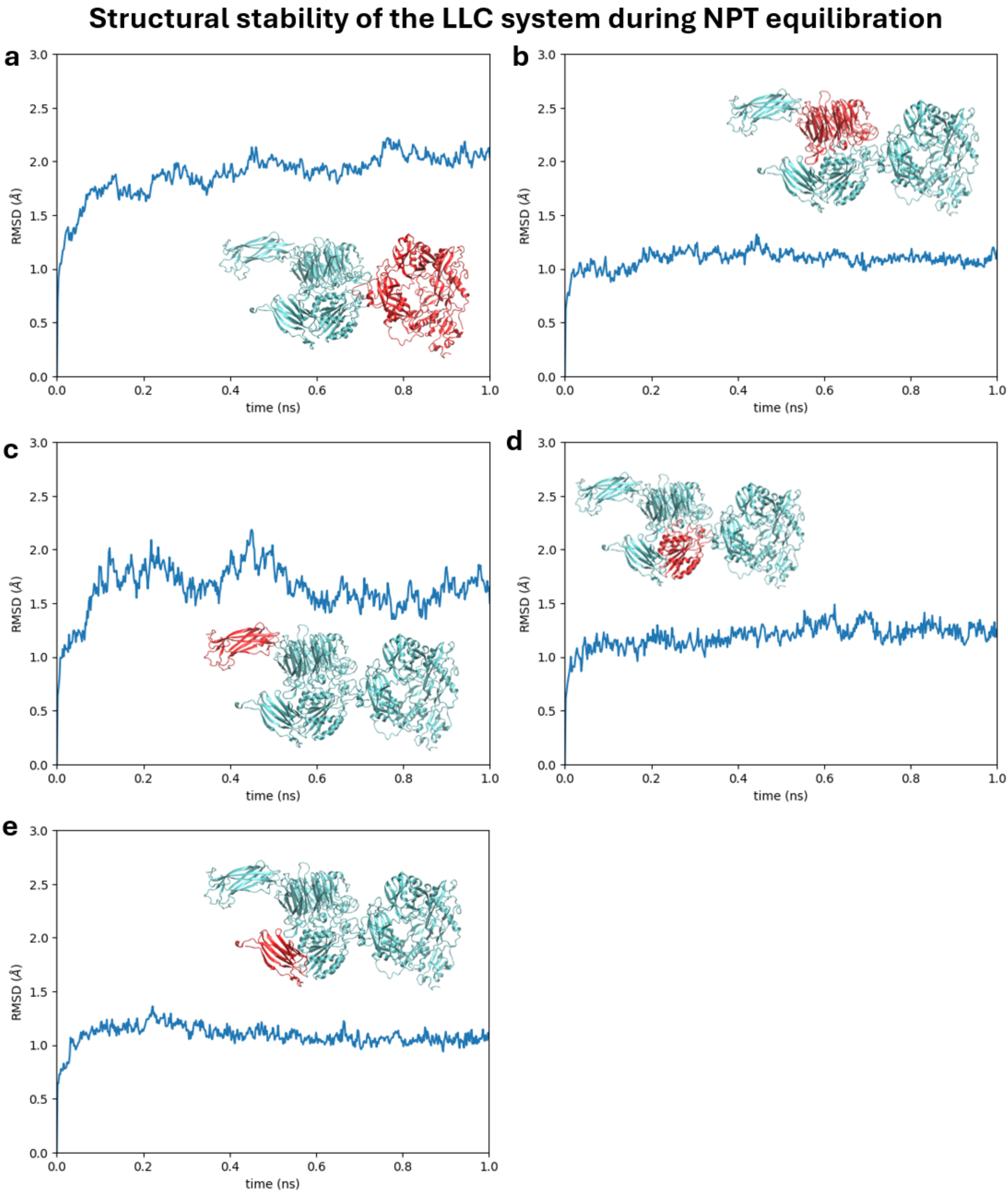

Supplementary Figure 19. Structural stability of the LLC system during NPT equilibration. Root mean square deviation (RMSD) of individual domains in the LLC during 1ns NPT process. The red region in the structural snapshot indicates the analyzed domain. (a) LAP + GF + TB2, (b) β-propeller, (c) αv, (d) βI, and (e) β6.

	pro-TGF-beta1 in complex with the third TB Domain from Latent Transforming Growth Factor-beta Binding Protein-1 (EMDB-53558) (PDB 9R3S)
**Data collection and processing**	
Magnification	165,000
Voltage (kV)	300
Electron exposure (e–/Å^2)	50.0
Defocus range (µm)	-2.0 to -0.75
Pixel size (Å)	0.72
Symmetry imposed	C1
Initial particle images (no.)	19,164,772
Final particle images (no.)	124,397
Map resolution (Å)	3.06
FSC threshold	0.143
Map resolution range (Å) (min, median, max)	2.356, 2.988, 7.656
**Refinement**	
Initial model used (PDB code)	5VQP
d Model resolution (Å)	3.4
Map sharpening *B* factor (Å^2)	0.00
Model composition	
Non-hydrogen atoms	11803
Protein residues	723
Ligands	BMA: 4, NAG: 9, FUC: 2
B factors (Å^2)	
Protein	105.26
Ligand	145.74
R.m.s. deviations	
Bond lengths (Å) (# > 4σ)	0.005 (0)
Bond angles (°) (# > 4σ)	0.990 (0)
Validation	
MolProbity score	2.02
Clash score	8.30
Poor rotamers (%)	0.94
Ramachandran plot	
Favored (%)	89.22
Allowed (%)	10.78
Disallowed (%)	0.00

Supplementary Table 1. Cryo-EM data collection, refinement and validation statistics
