# Supplementary material for: Structural basis for the contribution of latent TGFβ binding protein to TGFβ latency and activation
Source: Nat Commun. 2026 Aug 5;17:7619. doi: 10.1038/s41467-026-75834-8 (PMC13443091; doi:10.1038/s41467-026-75834-8)
Supplement: Supplementary file 2 [file 41467_2026_75834_MOESM2_ESM.pdf]

nature portfolio

Corresponding author(s): Clair Baldock

Last updated by author(s): Jun 30, 2026

Reporting Summary

Nature Portfolio wishes to improve the reproducibility of the work that we publish. This form provides structure for consistency and transparency in reporting. For further information on Nature Portfolio policies, see our Editorial Policies and the Editorial Policy Checklist.

Statistics

For all statistical analyses, confirm that the following items are present in the figure legend, table legend, main text, or Methods section.

n/a	Confirmed	
☐	☒	The exact sample size (n) for each experimental group/condition, given as a discrete number and unit of measurement
☐	☒	A statement on whether measurements were taken from distinct samples or whether the same sample was measured repeatedly
☐	☒	The statistical test(s) used AND whether they are one- or two-sided<br>*Only common tests should be described solely by name; describe more complex techniques in the Methods section.*
☐	☒	A description of all covariates tested
☐	☒	A description of any assumptions or corrections, such as tests of normality and adjustment for multiple comparisons
☐	☒	A full description of the statistical parameters including central tendency (e.g. means) or other basic estimates (e.g. regression coefficient) AND variation (e.g. standard deviation) or associated estimates of uncertainty (e.g. confidence intervals)
☐	☒	For null hypothesis testing, the test statistic (e.g. F, t, r) with confidence intervals, effect sizes, degrees of freedom and P value noted<br>*Give P values as exact values whenever suitable.*
☒	☐	For Bayesian analysis, information on the choice of priors and Markov chain Monte Carlo settings
☒	☐	For hierarchical and complex designs, identification of the appropriate level for tests and full reporting of outcomes
☒	☐	Estimates of effect sizes (e.g. Cohen's d, Pearson's r), indicating how they were calculated

Our web collection on statistics for biologists contains articles on many of the points above.

Software and code

Policy information about availability of computer code

Data collection	CryoEM data - EPU (Thermofisher)
Data analysis	CryoEM data analysis - cryoSPARC v2, MotionCor2 v1.2.0, Topaz.<br>Structure refinement - COOT (0.9.8.7) and Phenix real-space-refine.<br>Structure visualisation - UCSF ChimeraX 1.7, Pymol.<br>Structure validation - Molprobity 4.5.2. and the wwPDB validation system, glycans were assessed using Privateer.<br>Visualisation of graphs - Prism version 10.3.0 (Graphpad)<br>Steered molecular dynamics simulation - GROMACS 2024 - CHARMM 36<br>Octet-BLI - Octet Data Analysis HT software

For manuscripts utilizing custom algorithms or software that are central to the research but not yet described in published literature, software must be made available to editors and reviewers. We strongly encourage code deposition in a community repository (e.g. GitHub). See the Nature Portfolio guidelines for submitting code & software for further information.

Data

Policy information about availability of data

All manuscripts must include a data availability statement. This statement should provide the following information, where applicable:

- Accession codes, unique identifiers, or web links for publicly available datasets
- A description of any restrictions on data availability
- For clinical datasets or third party data, please ensure that the statement adheres to our policy

The structure and cryo-EM data generated in this study have been deposited in the EMDB and PDB databanks under accession codes PDB ID 9R3S; EMD-53558. The input simulation files underlying Figure 5 and 6, and Supplementary Figures 6-9, 11 and 14-19 are available on figshare. The source data underlying Figures 3B-E, 4C and D, and Supplementary Figures 1, 4 and 5 are provided as a Source Data file. All data supporting the results of this study can be found in the article, supplementary, on figshare and source data file.

Research involving human participants, their data, or biological material

Policy information about studies with human participants or human data. See also policy information about sex, gender (identity/presentation), and sexual orientation and race, ethnicity and racism.

Reporting on sex and gender	N/A
Reporting on race, ethnicity, or other socially relevant groupings	N/A
Population characteristics	N/A
Recruitment	N/A
Ethics oversight	N/A

Note that full information on the approval of the study protocol must also be provided in the manuscript.

Field-specific reporting

Please select the one below that is the best fit for your research. If you are not sure, read the appropriate sections before making your selection.

[x] Life sciences [ ] Behavioural & social sciences [ ] Ecological, evolutionary & environmental sciences

For a reference copy of the document with all sections, see nature.com/documents/nr-reporting-summary-flat.pdf

Life sciences study design

All studies must disclose on these points even when the disclosure is negative.

Sample size	For cryoEM imaging 30,000 movies were collected and 4,948,982 particles picked after Topaz training.<br>For the luciferase reporter assays, sample size is based on previous experience in detecting significant differences.<br>No sample size calculations were performed for other experiments.
Data exclusions	The cryoEM pipeline excludes “bad” particles in an automated software pipeline that is widely utilised in the field and is well-described.<br>For all other data types, no data were excluded from analysis.
Replication	All attempts at replication were successful.<br>Biochemical experiments were repeated at least three times.
Randomization	Randomization is not relevant for this study, as there were no groups allocated in any of the experiments.
Blinding	Blinding was not relevant for the cryoEM data collection as data was collected on only one sample.<br>For MD simulations, blinding was not possible as the input structure would identify the sample being analysed.<br>No blinding was performed for the luciferase reporter assays as all measurements are performed in non-biased manner using an automated plate reader.

Reporting for specific materials, systems and methods

We require information from authors about some types of materials, experimental systems and methods used in many studies. Here, indicate whether each material, system or method listed is relevant to your study. If you are not sure if a list item applies to your research, read the appropriate section before selecting a response.

Materials & experimental systems

n/a	Involved in the study	
☐	☒	Antibodies
☐	☒	Eukaryotic cell lines
☒	☐	Palaeontology and archaeology
☒	☐	Animals and other organisms
☒	☐	Clinical data
☒	☐	Dual use research of concern
☒	☐	Plants

Methods

n/a	Involved in the study	
☒	☐	ChIP-seq
☒	☐	Flow cytometry
☒	☐	MRI-based neuroimaging

Antibodies

Antibodies used	Monoclonal antibody to StrepII tag. IBA Life Sciences: Code : 2-1507-001 (lot number 1507-0066). Antibody stock is prepared at 0.2mg/ml and used at a 1:2000 dilution.<br><br>Monoclonal antibody to FLAG-tag. Cell Signalling Technology, Code : 14793S (Clone D6W5B; lot number 7). Antibody used at 1:10,000 dilution.
Validation	https://www.iba-lifesciences.com/strepmab-classic/2-1507-001<br>https://www.cellsignal.com/products/primary-antibodies/dykddddk-tag-d6w5b-rabbit-mab-binds-to-same-epitope-as-sigma-aldrich-anti-flag-m2-antibody/14793

Eukaryotic cell lines

Policy information about cell lines and Sex and Gender in Research

Cell line source(s)	Expi293F cells - Source: ThermoFisher Scientific - Cat# A14527.<br>HEK293-EBNA cells - Source: ATCC Cat# CRL-10852, RRID:CVCL_6974.<br>Transformed Mink lung cells are from Professor Dan Rifkin, NYU Langone health https://doi.org/10.1006/abio.1994.1042
Authentication	Expi293F is a commercial cell line from ThermoFisher and comes with a certificate of analysis.<br>HEK293-EBNA (RRID:CVCL_6974) are from ATCC https://www.atcc.org/<br>Transformed mink lung cells (TMLC) are from Professor Dan Rifkin, New York University, Langone Health, USA. https://doi.org/10.1006/abio.1994.1042. For every assay, an active TGFbeta standard curve is prepared to check and validate the cells.
Mycoplasma contamination	Cells are mycoplasma free and routinely tested for mycoplasma contamination.
Commonly misidentified lines<br>(See ICLAC register)	N/A

Plants

Seed stocks	*Report on the source of all seed stocks or other plant material used. If applicable, state the seed stock centre and catalogue number. If plant specimens were collected from the field, describe the collection location, date and sampling procedures.*
Novel plant genotypes	*Describe the methods by which all novel plant genotypes were produced. This includes those generated by transgenic approaches, gene editing, chemical/radiation-based mutagenesis and hybridization. For transgenic lines, describe the transformation method, the number of independent lines analyzed and the generation upon which experiments were performed. For gene-edited lines, describe the editor used, the endogenous sequence targeted for editing, the targeting guide RNA sequence (if applicable) and how the editor was applied.*
Authentication	*Describe any authentication procedures for each seed stock used or novel genotype generated. Describe any experiments used to assess the effect of a mutation and, where applicable, how potential secondary effects (e.g. second site T-DNA insertions, mosiacism, off-target gene editing) were examined.*
