# Supplementary material for: Structural basis for the contribution of latent TGFβ binding protein to TGFβ latency and activation
Source: Nat Commun. 2026 Aug 5;17:7619. doi: 10.1038/s41467-026-75834-8 (PMC13443091; doi:10.1038/s41467-026-75834-8)
Supplement: Supplementary file 3 [file 41467_2026_75834_MOESM3_ESM.pdf]

Peer Review File

Structural basis for the contribution of latent TGFβ binding protein to TGFβ latency and activation

Corresponding Author: Professor Clair Baldock

This file contains all reviewer reports in order by version, followed by all author rebuttals in order by version.

Version 0:

Reviewer comments:

Reviewer #1

(Remarks to the Author)
This manuscript presents a highly significant study that advances our understanding of TGFβ regulation by integrating high-resolution structural biology, biochemical assays, mutagenesis, and molecular dynamics simulations. The 3.06 Å cryo-EM structure of the large latent complex (LLC) is a particular strength, providing critical structural insights. In addition, the study offers mechanistic understanding of TGFβ latency and activation. While the work is both novel and impactful, clarifications regarding experimental replication, simulation protocols, and mechanistic interpretation are needed to ensure reproducibility and to strengthen the connection between structural observations and biological function. Addressing these points would substantially enhance the rigor and overall impact of the manuscript.

Major points:
-Biological replicates: The number of independent replicates in TGFβ bioassays should be explicitly stated.
-Pulling protocol rationale: Justify the choice of pulling rate (0.05 nm/ns) and the selection of COM versus anchor points in MD simulations.
-Spring constants: Explain the different spring constants for SLC (1000 kJ/mol/nm²) versus LLC (40000 kJ/mol/nm²) and their effect on cross-system comparability.
-Equilibration details: Report durations of NVT/NPT steps, whether restraints were applied during NPT, and how convergence of temperature, pressure, and structural relaxation was assessed.
-Analysis of force and conformational events: Clarify how rupture events, bond lifetimes, and conformational changes were detected, measured, and quantified in simulations.
-Linking structure to function: Expand discussion on how MD-observed destabilization correlates with TGFβ activity in cells.
-Mutagenesis interpretation: Discuss why TGFβ W330A increases activity while LTBP1 F1387A does not, despite partial disruption of the covalent complex.
-MD limitations: Provide context on assumptions and limitations of simulations, including inferred rather than experimentally validated causal links to TGFβ release.

Minor points:
-Consider periodic boundary effects for long pulling distances relative to the simulation box.
-Clarify whether backbone and sidechains were restrained differently during equilibration.
-Describe loop minimization, equilibration, and validation for the β6 hybrid domain merged from PDB 4UM9.
-Discuss limitations of simulations in reflecting in vivo force orientations, cooperative effects, or cell-surface constraints.

Reviewer #2

(Remarks to the Author)
The manuscript by Biggin et al describes the structure of latent-TGF-β in disulfide-linked complex with LTBP1, the scaffolding protein that is essential for its secretion and storage in ECM. The paper interrogates also the requirements for associate between the two proteins and the mechanism of activation by integrins.
The main result of this story is the determination of the cryo-EM structure of the large-latent complex (LLC) of pro form of TGF-β with a fragment of LTBP1, from which is only a single domain is well resolved in the EM map. The structure shows

perhaps surprisingly intimate association between the two proteins with a significant hydrophobic interaction mediated by a cluster of aromatic residues. Mutation of some of those residues results in reduced formation of the complex, while not weakening it once formed. Nice display of how correct disulfide bond formation is dependent on correct association of the proteins non-covalently.
This work complements the earlier work by Springer group and others who have determined structures of latent-TGF-β in complex with other binding partner GARP, which forms a similar disulfide-bonded complex, but with completely different architecture due to unrelated structures. Some common features emerge still. Such as the poorly visible loop in the straightjacket that becomes strictured on both LTBP and GARP complexes.
This structure also confirms the contralateral architecture for latent-TGF-β, with the so-called straightjacket part of the protein and the globular arm domain are contacting different halves of the dimeric mature domain. Molecular dynamics simulations of activation mechanism support this as well, suggesting more stable complex needing higher force to release the mature growth factor.
Overall, an important contribution to understanding the regulation of TGF-β ligands and their activation from the latent complexes. The work is high quality and results consistent with previous work/knowledge and functional interrogation of the results and MD simulations supporting the structural work nicely.
Nothing major to revise in my view. The only small detail that seemed to be missing was mention and acknowledgement of the fact that arm domains of TGF-β prodomains, in contrast to activins, BMPs and GDFs, are linked by disulfide in the so-called bowtie motif (next to the integrin binding site). That means the pulling force on one arm domain is transmitted to the other one, as seen in the molecular dynamics simulation figure 5. As this is not a feature of other TGF-β ligands, perhaps worth highlighting.
A few small suggestions and/or corrections:
In Figure 1G cartoon the α1-helices are depicted as arrows. Little misleading as arrows in structures are β-strands. Maybe change to more intuitive representation?
The curve in Figure 3E a little odd as it is essentially EC50 curve but in reverse. Could it not be fitted to derive EC50s? Seem like almost full curves. Alsa, what is going on with the label of the Y-axis? X-axis is protein concentration and Y-axis is “TGF-β concentration”? Surely it should be luciferase signal?
In the fitting of the BLI data in figure 4D is looks like off-rate is faster than the data suggests, which could be due to not all the ligand dissociating thanks for avidity coming from multi-dentate binding of the dimeric ligand to the sensor (common issue)? Fitting to a partial dissociation model might work better while not making massive difference to numbers overall.

Reviewer #3

(Remarks to the Author)

The contribution by Biggin et al. builds on resurging interest on the biogenesis, activation of TGFβ1 and its pleiotropic role in cellular physiology and disease as a function of the context of its presentation.
The authors will need to address several issues as itemized below per relevant section in the manuscript.

Introduction
==========
The authors state: ‘This is in contrast to activation by αVβ8, which can activate TGFβ from the SLC by either protease-dependent processes18,19 or independently of the release and diffusion of TGFβ20,21.’
Reference 21 does not claim that activation via integrin αVβ8 happens independently of the release of TGFβ!

The authors stated: Current therapeutic strategies targeting TGFβ focus on global inhibition of signalling, which often has severe toxicities26.’
This is true but this reviewer has identitied recent reports that have also provided a context for evoking activating mechanisms, e.g. Lambert et al., Cell Reports, 2025 https://doi.org/10.1038/s41594-023-01190-6.

Results
======
The authors stated: ‘There was only weak density for the LTBP1 domains downstream of TB2 (Supplementary Fig 3), and fibrillin-1 was not resolved, presumably owing to the flexibility of these domains.
AF3 models of LTBP1 indeed suggest flexibility between the domains, maybe worth to show in a Supplementary Figure? Also, was any analysis that addresses conformational variability tried (e.g. 3D Variability analysis and/or 3D Flexible Refinement in cryoSPARC, or DynaMight in Relion) to increase density for the LTBP1 domains downstream of TB2?

Figure 1: could benefit from a few zoomed regions showing the model in its corresponding cryo-EM density.
The authors stated: “This is similar in the GARP-SLC structure24, where this region is more ordered upon GARP binding, …”: It would be appropriate to also cite reference 21 here. Same comment applies to Figure 4 and related text.
The authors stated in the section “Conformational rearrangement of the integrin-binding RGD loop in LAP does not change affinity for αvβ6” that “However, kinetic analysis showed no significant difference in the amount binding or the affinity of the LLC or SLC for integrin αVβ6 (Figure 4E). This suggests that the RGD-loop is mobile in solution and freely available to bind αVβ6 in both complexes.”
This reviewer does not see how binding kinetics via BLI can point to a suggestion/conclusion about the degree of availability for binding. All the data shows is that the affinity and binding kinetics are the same!

The authors will need to correct their main text, figures and figure legends to adopt the standard nomenclature for denoting kinetic binding parameters, such that all letters with a K or k should be in italic and all subscripts D/a/d/ in non-italicized characters.

Discussion
==========
The authors write: “A similar feature was recently reported in the cryo-EM structure of GARP-SLC (ref 24), however a hydrophobic interaction was not present between TGFβ and GARP.”:
Upon examining the relevant literature this reviewer wishes to caution that this feature was already observed and described in Liénart et al., Science, 2018 (Figure 2A)! This is 7 years earlier than the cited work paper by Jin et al. 2025.
In general the reader gets the impression that the authors consider the paper by Jin et al. 2025 as the reference for GARP-TGFb complexes.
Also, how does recent work by Jin et al. 2024 Cell (https://doi.org/10.1016/j.cell.2019.12.030) relate to the presented work in this manuscript. Surprisingly the authors did not discuss this work.
Therefore, this reviewer strongly recommends a more complete and balanced discussion of prior work in the field.

Methods section
==============
Map-Model FSC curves are missing, Map-Model FSC needs to be reported (using the 0.5 threshold).
How was postprocessing/sharpening of the final map performed? Which negative B factor was used for sharpening? The Refinement section of Supplementary Table 1 lists a B factor of 0, was this unsharpened map only used for refinement or also for building in Coot and display in figures?

Reviewer #4

(Remarks to the Author)
I co-reviewed this manuscript with one of the reviewers who provided the listed reports. This is part of the Nature Communications initiative to facilitate training in peer review and to provide appropriate recognition for Early Career Researchers who co-review manuscripts.

Version 1:

Reviewer comments:

Reviewer #1

(Remarks to the Author)
The authors have responded thoroughly to the reviewers' comments and have satisfactorily addressed my concerns.

Reviewer #2

(Remarks to the Author)
I am simply reviewing the revisions, nothing fundamentally changed with respect the manuscript as a whole.
I am happy with the responses both to my own points and to what other referees have asked. Several points rectified and clarified, making the whole manuscript more readable.
The authors do push back on the BLI fitting and their point is clear and fine for me, especially as the relative affinities stay similar.
Happy to recommend publication.

Reviewer #3

(Remarks to the Author)
Overall, the revised manuscript addresses well most of the input provided to the authors.

However, the introduced nomenclature for the binding kinetics in Figure 4E is still problematic.

The two velocity rate constants for the association and dissociation of binding need to be in lower case 'k' not a capital K, and in addition need to be lower letter 'k' subscript italics 'a' and lower letter 'k' subscript italics 'd', in fact what the instrument software also reports. The derived affinity in terms of a KD is designated correctly.
Finally, the unit for seconds in each of the kinetic velocity constants is not a capital letter S but obviously a lower case s.

Such sloppiness and/or ignorance is alarming at this level of scientific publishing.

Reviewer #4

(Remarks to the Author)

I co-reviewed this manuscript with one of the reviewers who provided the listed reports. This is part of the Nature Communications initiative to facilitate training in peer review and to provide appropriate recognition for Early Career Researchers who co-review manuscripts.

Open Access This Peer Review File is licensed under a Creative Commons Attribution 4.0 International License, which permits use, sharing, adaptation, distribution and reproduction in any medium or format, as long as you give appropriate credit to the original author(s) and the source, provide a link to the Creative Commons license, and indicate if changes were made.
In cases where reviewers are anonymous, credit should be given to 'Anonymous Referee' and the source.
The images or other third party material in this Peer Review File are included in the article's Creative Commons license, unless indicated otherwise in a credit line to the material. If material is not included in the article's Creative Commons license and your intended use is not permitted by statutory regulation or exceeds the permitted use, you will need to obtain permission directly from the copyright holder.
To view a copy of this license, visit https://creativecommons.org/licenses/by/4.0/

Response to reviewers' comments

The authors thank the reviewers for their comprehensive comments and helpful suggestions. We have addressed each point as follows.

Reviewer 1:

Biological replicates: The number of independent replicates in TGFβ bioassays should be explicitly stated.

A: Data points represent the mean from three independent biological replicates. For each biological replicate, three technical replicates were also performed, and the value determined for each biological repeat was the mean of the three technical replicates. Error bars represent the standard deviation of the three biological replicates. The methods and figure legend for figure 3 have been amended to clarify these details.

Justify the choice of pulling rate (0.05 nm/ns) and the selection of COM versus anchor points in MD simulations.

We thank the reviewers for raising these questions regarding the setup of the SMD simulations. The pulling rate of 0.05 nm/ns was chosen based on the previous work by Dong et al. 2017, in which a series of SMD simulations were conducted using pulling rates of 1, 0.5, 0.1, and 0.05 nm/ns. We selected the slowest pulling rate among them for two reasons: (i) to enable a direct with their results, and (ii) because a slower pulling rate allows for better resolution of intermediate events and provides a less biased force–displacement profile.

Regarding the selection of the loading point during pulling, we chose to apply the force to the center of mass (COM) of the β6 hybrid domain rather than to an individual atom within the domain. For large domain complexes, applying force at a single point can lead to undesired partial unfolding or distortion of the tertiary structure within the steered domain, due to strong interactions with other parts of the complex (Cuendet & Michielin (2008)). Applying the pulling force to the COM, thereby distributing it uniformly across all atoms of the domain, represents a more reasonable and less biased approach, and is consistent with strategies commonly adopted in domain-level pulling studies (Rodriguez et al., 2015; Baker et al., 2013.).

Regarding the selection of anchoring points, in the SLC system we anchored two individual Cα atoms corresponding to the two Cys33 residues, as these cysteines in LAP connect LAP to LTBP1.

For the LLC system, we anchored the center of mass (COM) of the TB2 domain for several reasons. First, the resolved structure includes the TB2 domain, while the upstream and downstream cbEGF domains are not resolved at high resolution. As a result, there is some uncertainty in the exact positions of the domain termini. Moreover, these termini will not be free but connected to adjacent cbEGF domains. Furthermore, LTBP1 can attach to the extracellular matrix either via fibronectin at the N-terminus (Fontana et al., 2005) or fibrillin at the C-terminus (Isogai et al 2003; Lockhart-Cairns et al., 2022). Since force transmission could occur through either or both termini, selecting a single terminal residue as the anchoring point would introduce additional uncertainty. Under these circumstances, anchoring the COM of the TB2 domain provides a more balanced and unbiased representation of force transmission.

We have revised the following content in the Methods section of the manuscript:

Original:

"Molecular dynamics simulations were performed by pulling on the centre of mass (COM) of the integrin β6 hybrid domain at 0.05 nm/ns which was resisted by the Cα atoms in LAP Cys33 residues (Supplementary Fig 10). The LLC simulations were performed similarly but with our cryo-EM structure of the LLC replacing the SLC. Pulling in the LLC simulations was resisted from the COM of the LTBP1 TB2 domain."

Revised:

To facilitate comparison with previous literature, the representative pulling velocity reported by Dong et al. ($v = 0.05$ nm/ns) was adopted for SMD simulations[25]. The pulling force was applied to the β6 hybrid domain by distributing it evenly across all atoms within the domain, rather than applying the force to individual atoms as in Dong et al[25]. This strategy is intended to minimize artifacts such as partial unfolding and undesired distortion of the tertiary structure (Supplementary Fig. 10).

The LLC simulations were performed in a similar manner, with the cryo-EM structure of the LLC replacing that of the SLC. In the LLC setup, the pulling force was counterbalanced by anchoring the COM of the LTBP1 TB2 domain. The choice to anchor the COM, rather than individual atoms (e.g., terminal Cα atoms), is motivated by the unresolved upstream and downstream regions of the TB2 domain, as well as uncertainty in the direction of the resistant force, as LTBP1 binds to extracellular matrix proteins, fibronectin[17] and fibrillin[16,39] via N- or C-terminal regions, respectively.

Explain the different spring constants for SLC (1000 kJ/mol/nm^2) versus LLC (40000 kJ/mol/nm^2) and their effect on cross-system comparability.

We thank the reviewer for pointing out the missing explanation regarding the difference in anchoring spring constants between SLC and LLC. For SLC, atoms are restrained individually, meaning that each atom is associated with a single spring. In this case, a spring constant of 1000 kJ/mol/nm^2 is sufficient to mimic rigid fixation.

In contrast, in LLC, the anchor consists of a group of atoms within the TB2 domain that are restrained collectively. If the same spring constant used for a single atom (k = 1000 kJ/mol/nm^2) were applied, the restraint would be too weak, allowing the entire TB2 domain to move. Therefore, a larger spring constant (k = 40000 kJ/mol/nm^2) was selected. This value was determined through trial and error to ensure that the TB2 domain is effectively (rigidly) restrained, while the applied external force is not excessively strong, which could otherwise lead to system instability.

Overall, the criteria for determining the spring constant are twofold: achieving rigid fixation and maintaining system stability. Based on these criteria, we do not expect any issues regarding comparability between different systems.

We have added the following sentences into method section:

"The choice of spring constant depends on the number of atoms in the restrained group. In the SLC system, the anchor consists of a single atom, whereas in the LLC system, the entire TB2 domain is restrained, which requires a larger spring constant to achieve effective anchoring. However, excessively large spring constants may introduce instability due to unrealistically high external forces. Therefore, the value of k = 40000 kJ/mol/nm^2 was determined through trial and error, ensuring that the TB2 domain is effectively restrained while maintaining overall simulation stability"

Equilibration details: Report durations of NVT/NPT steps, whether restraints were applied during NPT, and how convergence of temperature, pressure, and structural relaxation was assessed.

NVT equilibration was performed for 40 ps with positional restraints applied to all heavy atoms of the protein to relax surrounding water molecules insufficiently minimized during energy minimization. This was followed by a 1 ns NPT simulation without restraints to equilibrate temperature and pressure. System properties, including temperature, pressure, and density, showed stable convergence.

Protein structural relaxation was assessed by monitoring protein–protein and protein–nonprotein interaction energies, both of which reached steady values. Owing to the large flexibility of the domain complex, RMSD was evaluated on a per-domain basis by partitioning the structure at hinge regions to exclude global domain motions. The RMSD of individual domains converged, confirming structural relaxation of the protein (new Supplementary figures 13-18)

We have revised the following content in the Methods section of the manuscript:

Original:

"Prior to steered molecular dynamics (SMD) simulations, the systems underwent energy minimization using the steepest descent algorithm, followed by an NVT ensemble simulation with all heavy atoms restrained to relax the solvent. Subsequently, an NPT ensemble simulation was conducted to equilibrate temperature and pressure of the system."

Revised:

"Prior to steered molecular dynamics (SMD) simulations, the systems underwent energy minimization using the steepest descent algorithm, followed by a 40 ps NVT ensemble simulation with all heavy atoms restrained to relax

the solvent surrounding the protein. Subsequently, a 1 ns NPT ensemble simulation was conducted to equilibrate the system temperature and pressure without any atomic restraints. The root mean square deviation (RMSD) of protein domains was analyzed individually to exclude large-scale domain motions from the RMSD calculation. During the 1 ns NPT simulation, all systems converged in temperature, pressure, and density, and the protein structures relaxed (Supplementary figures 14-19)."

Analysis of force and conformational events: Clarify how rupture events, bond lifetimes, and conformational changes were detected, measured, and quantified in simulations.

We thank the reviewer for this important comment and have clarified the procedure used to identify and quantify force and conformational events in the revised manuscript.

Specifically, force events were defined as local maxima in the force–pulling distance curve. Conformational changes in the protein complex were characterized based on the interactions between the growth factor region and the arm domain, which comprises the fastener and α2 helix. The system contains two chains for both the growth factor and arm domains (denoted as chain C and chain D; Supplementary Fig. S7, S9 and S11).

To quantitatively assess these interactions, the buried surface area was computed between the growth factor domain and each of the fastener and α2 helix regions from both chains, resulting in eight interaction pairs in total. A conformational event was operationally defined as the loss of interaction between a given pair, identified when the corresponding buried surface area decreased to zero.

These conformational events were then temporally correlated with force peaks to establish the relationship between mechanical rupture events and the associated structural changes.

As an illustrative example is shown below, one prominent force peak corresponds to the dissociation ("snapping") between the C- and D-chain fastener and the C-chain growth factor domain (Figure 5D, left panel). In this case, the buried surface area between these two components decreases from a finite value to zero at a pulling distance of ~12 nm (Supplementary Figure 7C), indicating complete loss of interaction. This transition occurs concurrently with a local maximum in the force–extension curve at ~12 nm (as seen in Figure 5A), demonstrating a direct correspondence between the mechanical rupture event and the underlying conformational change as defined by our analysis protocol.

Figure 5D Left panel

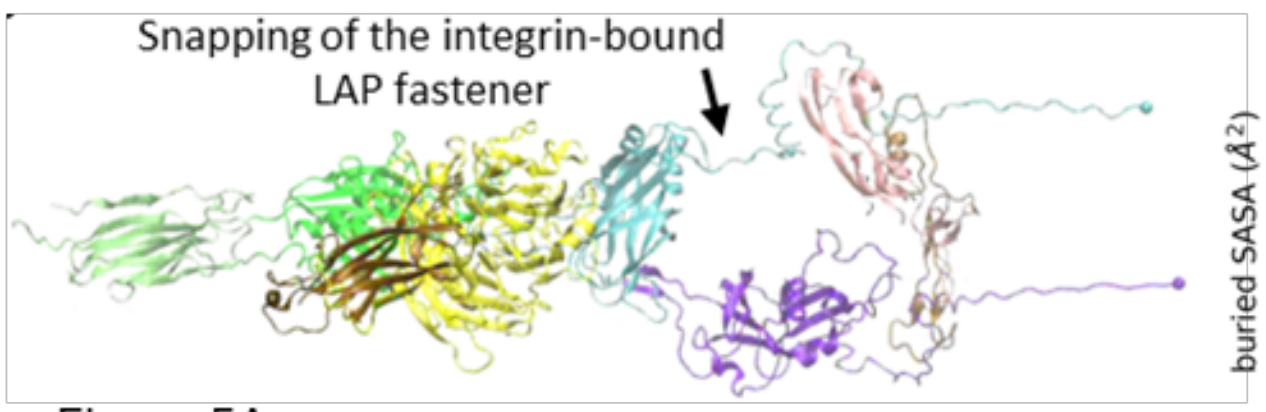

Supplementary Figure 7C

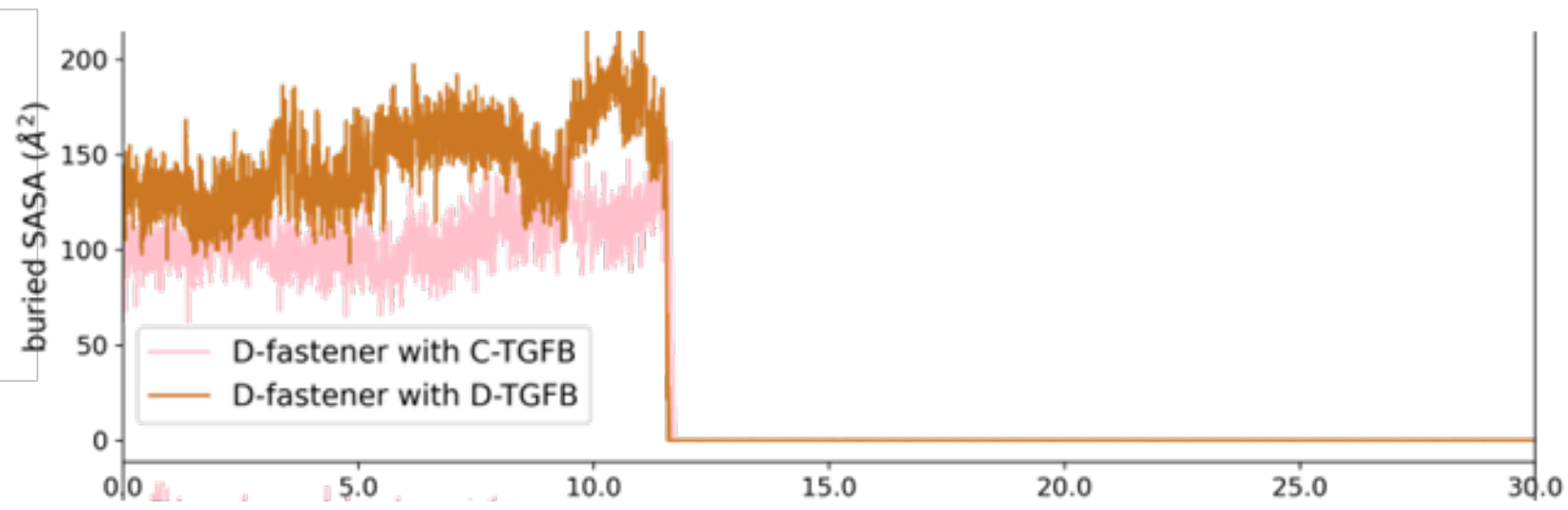

Figure 5A

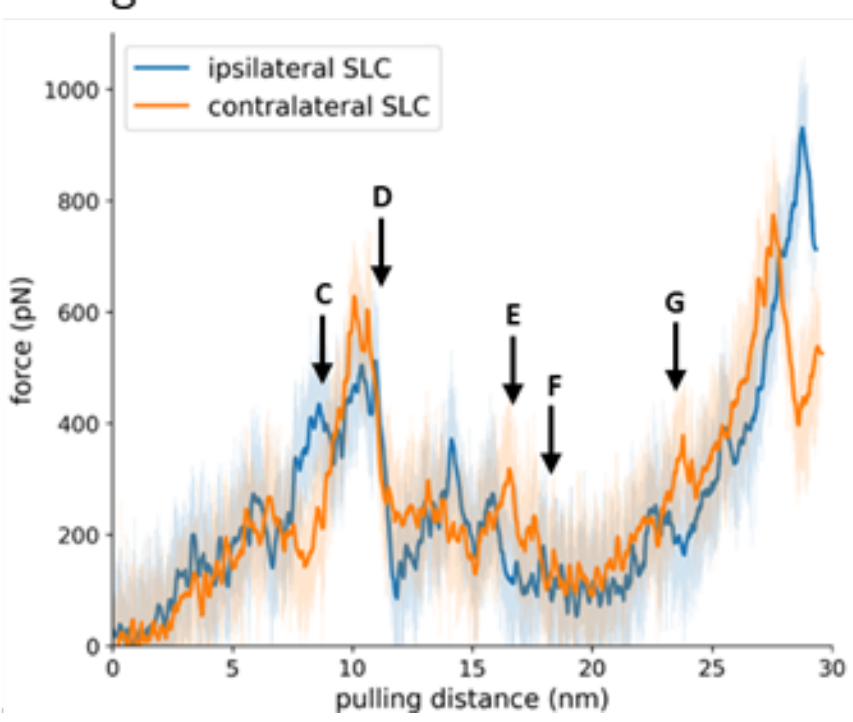

To clarify these details, we have added the following sentences into the method section:

"Force events were defined as local maxima in the force–pulling distance curve. Conformational changes in the protein complex were characterized based on the interactions between the growth factor region and the arm domain, which includes the fastener and α2 helix. The system consists of two chains for both the growth factor

and arm domains (denoted as chain C and chain D; see Supplementary Figs. 7, 9 and 11). To quantify these interactions, the buried surface area was calculated between the growth factor domain and each of the fastener and α2 helix regions from both chains, resulting in eight interaction pairs in total. A conformational event was defined as the loss of interaction between a given pair, operationally identified when the buried surface area decreased to zero. These events were then correlated with force peaks to establish the relationship between mechanical rupture and structural changes."

Expand discussion on how MD-observed destabilization correlates with TGFβ activity in cells.
We have added the following paragraph to the discussion: Force activation of TGFβ is mediated by integrin binding to the RGD loop in LAP, which enables the application of tensile force to unmask the TGFβ growth factor receptor binding sites by unfolding of LAP. The steps between LAP unfolding and TGFβ receptor binding are not yet defined molecularly, but TGFβ is in a different conformation when in complex with LAP compared to when it is bound to its receptors, and LAP also blocks receptor binding sites to provide latency (Radaev et al 2010; Shi et al 2011). The type II receptor binding site is blocked by the latency lasso, and the type I receptor binding site is blocked by the straitjacket α1 helix, the fastener and the arm domain (Shi et al., 2011). Mutation of key aromatic residues in the fastener, to release the fastening of the straitjacket to the arm domain, resulted in spontaneous TGFβ activation and conversely, engineering a disulphide bond to covalently fasten the straitjacket prevented integrin-mediated activation (Shi et al., 2011). The main force peaks observed in our SMD simulations were associated with unsnapping of each fastener and unwinding the α2 helices in the interfaces between the arm domain and the growth factor. In agreement with the study by Dong et al., the simulations predict that these are the most force-resistant elements and releasing the fastening of the straitjacket would allow TGFβ activation by revealing receptor binding epitopes.

Discuss why TGFβ W330A increases activity while LTBP1 F1387A does not, despite partial disruption of the covalent complex.
This is an interesting point, mutation of these residues in both TGFβ and LAP reduce the amount of covalent complex that forms, presumably by weakening the interaction between LTBP1 and TGFβ. However, as the reviewer indicates, for the latent complex that is formed the LTBP1 mutation does not alter TGFβ activity. Interestingly, F1387 forms a hydrogen bond through its backbone atoms with W1380 so mutation of the F1387 side-chain is unlikely to disrupt the backbone hydrogen bond and its interaction with W1380 may be retained. It is also possible there could be a structural rearrangement in the TB2 domain, so that W1380 could interact with W330 in TGFβ to compensate for the mutation of F1387. In fact, W1380 is conserved across the other LTBP family members that bind TGFβ, whereas F1387 is not. Whereas for the W330A mutation in TGFβ, as well as interacting with LTBP1, this region of the growth factor is adjacent to the α1 helix of the adjacent chain of LAP, thus the mutation may also destabilise this interaction to affect the latency lasso of LAP.

We have added the following point to the discussion: "For the latent complex that is formed the F1387A LTBP1 mutation does not alter TGFβ activity, whereas TGFβ with the W330 mutation increases activity. Interestingly, F1387 forms a hydrogen bond through its backbone atoms with W1380 so mutation of the F1387 side chain is unlikely to disrupt this hydrogen bond. It is also possible there could be a structural rearrangement in the TB2 domain, so that W1380 could interact with W330 in TGFβ to compensate for the mutation of F1387. Whereas for W330 in TGFβ, as well as interacting with LTBP1, this region of the growth factor is adjacent to the α1 helix of the adjacent chain of LAP, thus the mutation may also destabilise this interaction to affect the latency lasso of LAP. "

Provide context on assumptions and limitations of simulations, including inferred rather than experimentally validated causal links to TGFβ release.
We thank the reviewer for raising this important point, we have added the following paragraph to the Discussion section:
"Additionally, it is important to note that these simulations rely on several assumptions and simplifications inherent to molecular dynamics approaches. For example, the choice of pulling velocities, positional restraints,

and the treatment of unresolved regions in the protein complexes may influence the observed mechanical pathways. Consequently, while our results suggest that conformational changes in LAP and force transmission through LTBP1 facilitate TGFβ release, these causal relationships are inferred directly from computational observations. Some caution should therefore be exercised when extrapolating these findings to physiological conditions."

Minor points:

-Consider periodic boundary effects for long pulling distances relative to the simulation box. The shortest distance between periodic images during the simulation occurs in the x direction (pulling direction) after completion of pulling, with a value of approximately 4 nm. In the y and z directions, the shortest distances between periodic images are 8.7 nm and 9.9 nm, respectively, measured prior to the onset of pulling. All of these distances exceed three times the nonbonded interaction cutoff used in the force field, thereby satisfying standard criteria to avoid spurious self-interactions across periodic boundaries. Therefore, periodic boundary effects are expected to be negligible and should not influence the observed simulation results (see Response figure below).

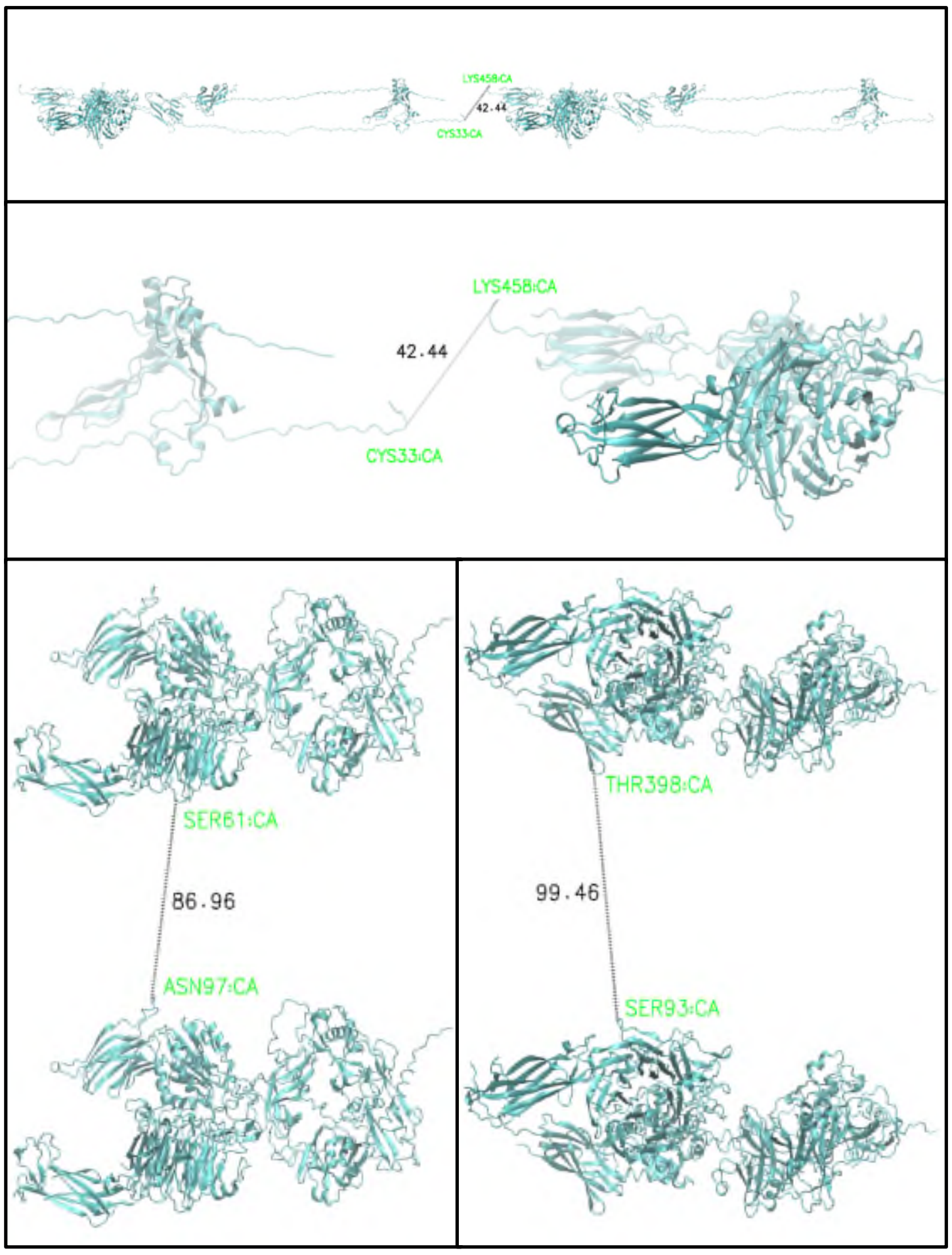

***Response figure**: Shortest distances between periodic images. (a) Distance in the x direction (pulling direction) after pulling, (b) zoomed-in view of (a), (c) distance in the y direction before pulling, and (d) distance in the z direction before pulling. For panels (a) and (b), measurements were taken from the post-pulling configuration, where the shortest distance between periodic images in the x direction is observed. For panels (c) and (d), measurements were taken from the pre-pulling configuration, where the shortest distances between periodic images in the y and z directions are observed.*

-Clarify whether backbone and sidechains were restrained differently during equilibration. During the NVT equilibration, positional restraints were applied to all heavy atoms, including both backbone and sidechain atoms (i.e., all non-hydrogen atoms). During the subsequent NPT equilibration, no restraints were applied and all atoms were free to move. This point has been clarified in the revised manuscript based on the reviewer's comment.

-Describe loop minimization, equilibration, and validation for the β6 hybrid domain merged from PDB 4UM9. The integrin β6 hybrid domain is not present in the SLC-integrin structure (PDB:5FFO)(Dong et al., 2017) but was used in the same study as the anchor point for pulling experiments. So, to replicate their experiment, the β6 hybrid domain was incorporated into the SLC-integrin model using the structure of αVβ6 with RGD peptide (PDB:4UM8)

as a template. The phenix "align with sequence" tool was used to align 4UM8 to 5FFO (chains E F) (RMS 1.59) and the β6 hybrid domain was merged into the model using junctions at residues 131-132 and 350-351 (numbering from P18564 · ITB6_HUMAN). Prior to steered molecular dynamics (SMD) simulations, the model was energy-minimized to remove steric clashes, followed by NVT equilibration with positional restraints applied to all heavy atoms to allow hydrogen atoms to relax to energetically favorable positions.

We have added further details to the methods section to expand on how the hybrid domain was incorporated into the model.

-Discuss limitations of simulations in reflecting in vivo force orientations, cooperative effects, or cell-surface constraints.

We have added the following paragraph to the end of the discussion addressing these limitations, integrating it with the previous comment above on the simulation limitations:

"We further acknowledge several limitations in capturing the full complexity of the in vivo environment. First, the applied pulling direction in our simulations is defined and fixed, whereas in vivo force orientations are heterogeneous and dynamically regulated by the cellular context, including cytoskeletal attachments and extracellular matrix constraints. Experimental studies have shown that forces applied to cell surface receptors can propagate through receptor–cytoskeletal linkages and sometimes cause intracellular dissociation prior to rupture of the extracellular bond (Evans & Kinoshita (2007)). Similarly, in the context of TGFβ, the mechanical stress applied to the LAP–LTBP1 complex may propagate through multiple protein domains, triggering conformational changes that facilitate growth factor release. Therefore, the rupture pathways observed in our simulations likely represent a subset of physiologically accessible force transmission routes. Furthermore, the simulations focus on an isolated protein complex and do not account for cooperative effects arising from multivalent interactions, clustering, or neighboring molecules. In living cells, multiple molecular linkages may act cooperatively to modulate force propagation, dissociation kinetics, and mechanical stability, potentially altering the efficiency or timing of TGFβ activation. Cell-surface constraints, including membrane anchoring, spatial confinement, and steric hindrance from surrounding proteins or glycocalyx components, are not explicitly modeled. These factors could influence both the magnitude and directionality of forces, as well as the accessibility of conformational transitions required for TGFβ release. Taken together, while our simulations provide mechanistic insight into force-induced conformational changes in the LAP–LTBP1 complex, some caution should be exercised when extrapolating these results directly to physiological conditions. Nonetheless, analogies with receptor–cytoskeletal force propagation observed in other molecular systems suggest that nanoscale force transmission can have significant functional consequences, supporting the plausibility of mechanically mediated TGFβ activation."

Reviewer #2

Mention and acknowledgement that arm domains of TGF-β prodomains, in contrast to activins, BMPs and GDFs, are linked by disulfide in the so-called bowtie motif (next to the integrin binding site). That means the pulling force on one arm domain is transmitted to the other one, as seen in the molecular dynamics simulation figure 5.

We have added a sentence to the results mentioning this point "The arm domains of LAP, in contrast to the prodomains of other TGFβ superfamily members such as activins, BMPs and GDFs, are linked by a disulfide bond in the bowtie motif (Hinck et al, 2016), which results in the pulling force on one arm domain being transmitted to the other domain, as seen in the molecular dynamics simulations (Figure 5)".

A few small suggestions and/or corrections:

In Figure 1G cartoon the α1-helices are depicted as arrows. Little misleading as arrows in structures are β-strands. Maybe change to more intuitive representation?

In figure 1G, we had used arrows to indicate the directionality of the chain, rather than secondary structure, but could see how this could be misinterpreted. We have changed the cartoon to now represent the α1 and 2 helices as cylinders with an arrow superimposed to show the directionality of the chains.

Old figure 1G (left panel), revised figure 1G (right panel).

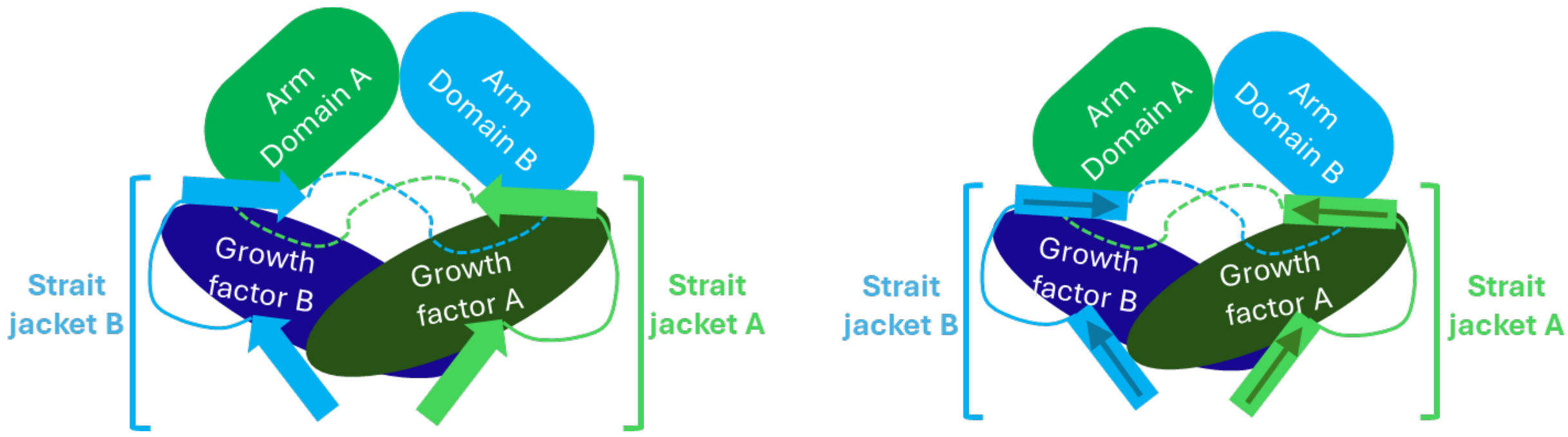

The curve in Figure 3E a little odd ..... what is going on with the label of the Y-axis? X-axis is protein concentration and Y-axis is "TGF-β concentration"? Surely it should be luciferase signal?

For the transformed mink lung cell assay, conventionally the luciferase activity is recorded as relative light units (RLU) then the RLU values are converted to TFGβ activity (pg/mL) using a TGFβ standard curve (Nunes et al., JCB 1997; Jurukovski et al., Methods in Molecular Medicine 2005). Therefore, the y-axis shows the amount of active TGFβ (pg/mL) calculated from the RLUs. For clarity, we have now changed the axis label to "equivalent amount of active TGFβ" and made the wording clearer in the figure legend and methods as follows – "The relative light units were converted to TGFβ activity using a standard curve determined for active TGFβ response".

In the fitting of the BLI data in figure 4D is looks like off-rate is faster than the data suggests, which could be due to not all the ligand dissociating thanks for avidity coming from multi-dentate binding of the dimeric ligand to the sensor (common issue)? Fitting to a partial dissociation model might work better while not making massive difference to numbers overall.

As suggested by the reviewer, the Octet-BLI data was fitted to a partial dissociation model which resulted in a small improvement in the fit of the off-rates, as shown below. The K_{off} and K_D measurements increase slightly for both SLC and LLC, with this model, but the binding of the SLC and LLC to αVβ6 are still very similar to each other. Our preference would be to keep the 1:1 model in figure 4, as although the off-rate analysis gives a better fit for dissociation, its measurement of K_D is an estimate based on the off-rate alone, whereas the 1:1 model is also taking into account the measured on-rate and so is a more complete analysis.

A

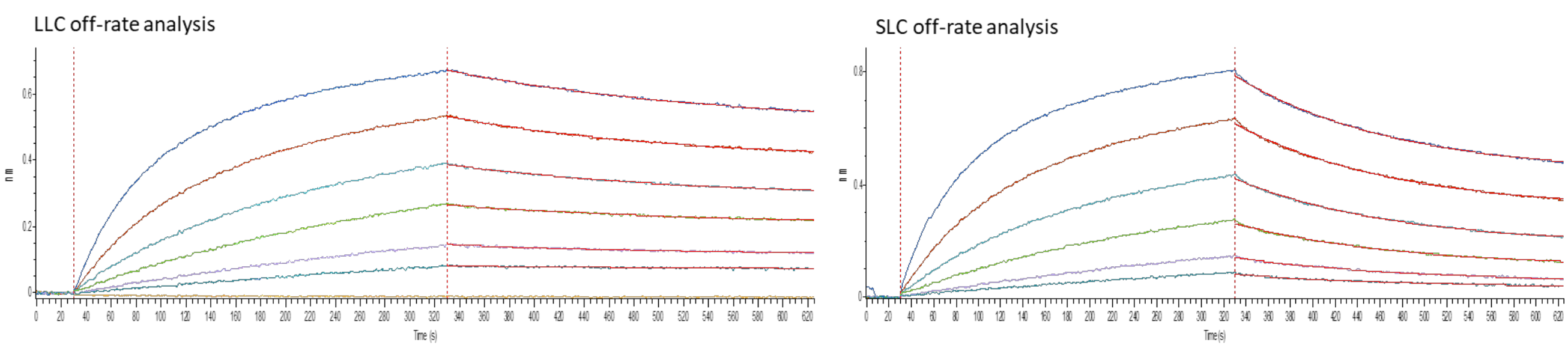

B

Analysis model	Sample	1:1 (global) vs off-rate (local)	
		K_D (nM)	K_{off} (1/s)
1:1 (global)	SLC	3.94	1.17 x 10-3
	LLC	2.95	0.85 x 10-3
Off-rate (local)	SLC	15.9	6.60 x 10-3
	LLC	13.7	5.56 x 10-3

***Response figure 2**: A. Sensograms of the binding of the LLC or SLC to the headpiece of integrin αVβ6 monitored using BLI on OctetRED96 (ForteBio). The measured responses, using a concentration range of 1.56-50 nM, were fitted to a partial dissociation model (red lines). B. Table comparing binding parameters K_D and K_{off} for data fitted with a 1:1 model and a partial dissociation model.*

Reviewer #3

The authors state: 'This is in contrast to activation by αVβ8, which can activate TGFβ from the SLC by either protease-dependent processes18,19 or independently of the release and diffusion of TGFβ20,21.'
Reference 21 does not claim that activation via integrin αVβ8 happens independently of the release of TGFβ!
Thank you for spotting this incorrect citation, we have removed reference to this citation for this point.

The authors stated: Current therapeutic strategies targeting TGFβ focus on global inhibition of signalling, which often has severe toxicities26.' This is true but this reviewer has identified recent reports that have also provided a context for evoking activating mechanisms, e.g. Lambert et al., Cell Reports 2025.
Thank you for highlighting TGFβ activating mAbs as therapeutics, we have amended this sentence as follows: "Current therapeutic strategies targeting TGFβ that focus on global inhibition of signalling often have severe toxicities[26], but recent findings indicate that mAbs that enhance TGFβ activation from membrane bound GARP-complexes may offer a more targeted therapeutic strategy (Lambert et a., 2025)."

The authors stated: 'There was only weak density for the LTBP1 ..... presumably owing to the flexibility of these domains." AF3 models of LTBP1 indeed suggest flexibility between the domains, maybe worth to show in a Supplementary Figure?
Thank you for this suggestion, we actually had previously shown experimentally the flexibility of different regions of LTBP1 using small angle X-ray scattering, including the C-terminal region used in the current study (Troilo et al., Sci Rep (2016) 6:34347 doi: 10.1038/srep34347). We have now added this citation to support this statement.

Also, was any analysis that addresses conformational variability tried (e.g. 3D Variability analysis and/or 3D Flexible Refinement in cryoSPARC, or DynaMight in Relion) to increase density for the LTBP1 domains?
Yes, a couple of different approaches were used in an attempt to address the conformational variability of LTBP1. Both 3D flex and 3D variability analysis were used in cryoSPARC but did not result in significantly increased density corresponding to the LTBP1 domains, and any density that was seen was not of an interpretable resolution.

Figure 1: could benefit from a few zoomed regions showing the model in its corresponding cryo-EM density.
Thank you for the suggestion, we have added two new panels to figures 1(F) and 2(E) showing the model in the corresponding cryoEM density.

The authors stated: "This is similar in the GARP-SLC structure24, where this region is more ordered upon GARP binding, ...": It would be appropriate to also cite reference 21 here. Same comment applies to Figure 4 and related text.
Reference 21 has now been cited where discussing figure 2H and 4.

The authors stated in the section "Conformational rearrangement of the integrin-binding RGD loop in LAP does not change affinity for αvβ6" that "However, kinetic analysis showed no significant difference in the amount binding or the affinity of the LLC or SLC for integrin αVβ6 (Figure 4E). This suggests that the RGD-loop is mobile in solution and freely available to bind αVβ6 in both complexes." This reviewer does not see how binding kinetics via BLI can point to a suggestion/conclusion about the degree of availability for binding. All the data shows is that the affinity and binding kinetics are the same!
We have reworded this sentence in the results to make this point clearer and removing reference to availability of binding. "However, kinetic analysis showed no significant difference in the amount binding or the affinity of the LLC or SLC for integrin αVβ6 (Figure 4E) indicating that binding of the SLC to LTBP1 does not change the interaction with αVβ6."

The authors will need to correct their main text, figures and figure legends to adopt the standard nomenclature for denoting kinetic binding parameters, such that all letters with a K or k should be in italic and all subscripts D/a/d/ in non-italicized characters.

This has been corrected in the text and figures.

The authors write: "A similar feature was recently reported in the cryo-EM structure of GARP-SLC (ref 24), however a hydrophobic interaction was not present between TGFβ and GARP.":
Upon examining the relevant literature this reviewer wishes to caution that this feature was already observed and described in Liénart et al., Science, 2018 (Figure 2A)!
Also, how does recent work by Jin et al. 2024 Cell relate to the presented work in this manuscript. In general the reader gets the impression that the authors consider the paper by Jin et al. 2025 as the reference for GARP-TGFb complexes. Surprisingly the authors did not discuss this work. Therefore, this reviewer strongly recommends a more complete and balanced discussion of prior work in the field.

Firstly, we wish to apologise and explain the omission of reference to the Jin et al. 2024 Cell paper. In early drafts of the manuscript, both Jin et al. 2024 Cell and Jin et al., 2024 biorxiv were cited in the manuscript. However, when the latter preprint was published (Jin et al 2025 Nat Comms), all Jin et al 2024 citations were updated in error to Jin et al 2025. We have now corrected this error in citation throughout the manuscript.

Indeed, the same region in TGFβ that interacts with LTBP1 forms an interface with GARP, shown in the structures from Liénart et al., Science, 2018 and Jin et al. 2024 Cell, but the nature of the interaction between TGFβ and LTBP1 is different to that with GARP. Whereas the Trp330 residue in TGFβ is involved in the interaction with both GARP and LTBP1, in LTBP1 it forms a contiguous pi-pi stacking interaction involving two aromatic residues in LTBP1. Whereas in GARP, Trp330 is involved in an interaction with the sidechains of Arg206 and Cys227. However, we agree that our statement that it is not a hydrophobic interaction with GARP was an over-simplification, so we have expanded this point in the discussion to explain in more detail the similarities and differences between the GARP/LTBP1-TGFβ interactions and included the appropriate citations referring to both LTGFβ-GARP structures as follows:

"This region in TGFβ also forms an interface with GARP as shown previously (Liénart et al., Science, 2018), and while W330 is involved in the interaction with both GARP and LTBP1, there are interesting differences between how LTBP1 and GARP achieve complementarity with the same conformer of TGFβ. In LTBP1, there is an extensive hydrophobic interaction where the helical structure in TGFβ is enclosed by a loop in LTBP1 (residues 1352-1358) along with the aforementioned pi-pi stacking interaction. Whereas in GARP, W330 interacts with the sidechains of R206 and C227, and the helical structure with M186 and V184 (Liénart et al., Science, 2018; Jin et al. 2024 Cell). The more extensive hydrophobic interface in LTBP1 may reflect the need for increased binding efficiency as the interaction with TGFβ is mediated by a smaller interaction domain."

Map-Model FSC curves are missing, Map-Model FSC needs to be reported (using the 0.5 threshold).
Thank you for highlighting this, the MAP/Model FSC curves have now been included in the supplementary information (Supplementary figure 13) with the masked FSC=0.5 resolution being 3.21Å.

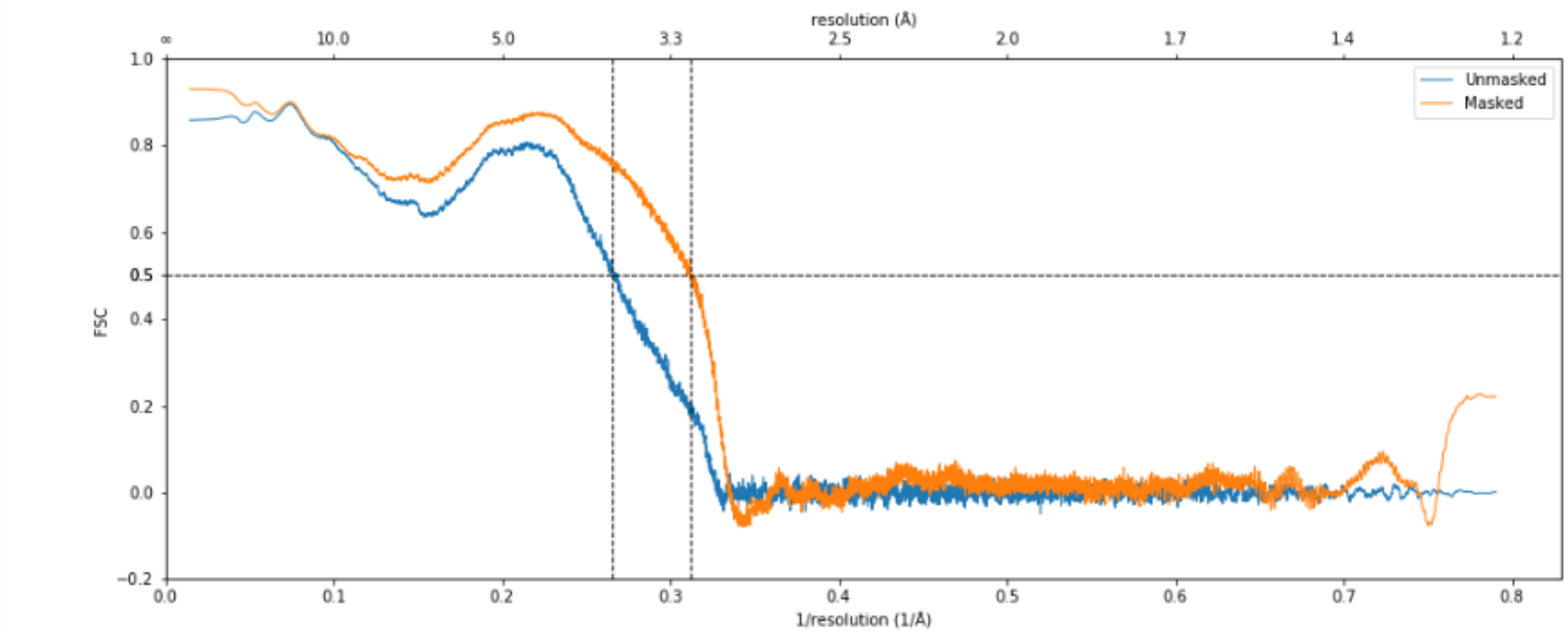

How was postprocessing/sharpening of the final map performed?

Postprocessing of the final map was done in cryoSPARC as part of the final local refinement procedure. A sentence describing this and what B-factor was applied has been added to the single-particle data processing section of the methods as follows: “The final map was sharpened in cryoSPARC as part of the local refinement procedure. A B-factor of-73.7 was applied to the map which was calculated from the Guiner plot.”

Which negative B factor was used for sharpening?

A B-factor of –73.7 was applied to the sharpened map, this was auto-calculated from the Guiner plot from cryoSPARC. This value has been added to the methods as outlined in the response above.

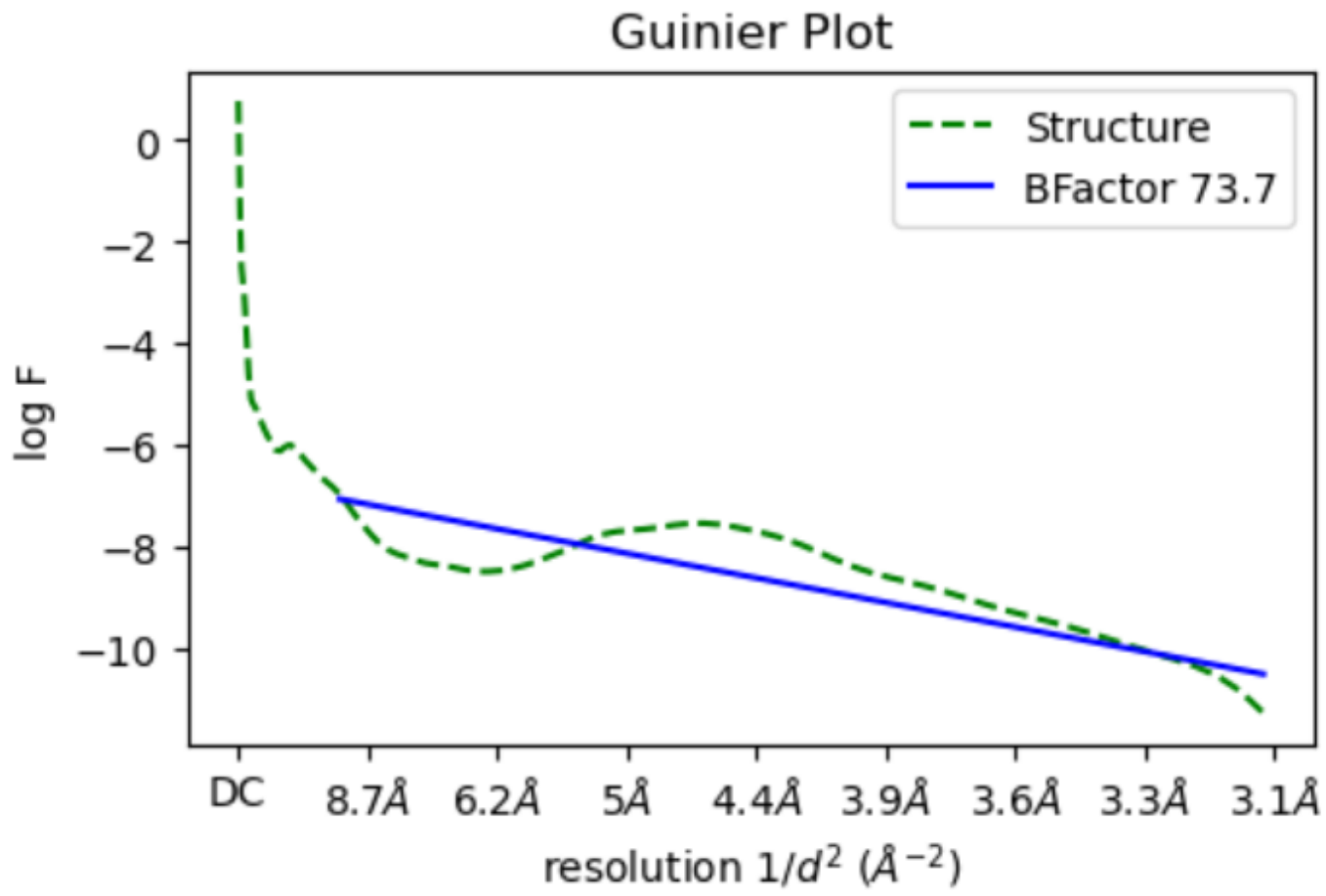

The Refinement section of Supplementary Table 1 lists a B factor of 0, was this unsharpened map only used for refinement or also for building in Coot and display in figures?

The structure contains some variability in the flexibility of different regions, so no single threshold or B factor was universally suitable for visualising all regions. Thus, a number of different sharpening B factors as well as the locally filtered map were used to inform model building and real-space refinement in coot. Features such as the glycans (on TGFβ and LTBP1) and the LAP disulphides are noisy in the sharpened map, whereas finer detail in core regions can be obtained with sharpening. To enable readers to assess the quality of evidence for all features we have deposited both sharpened and unsharpened maps into the EMDB (both maps are now also available to reviewers in figshare).

The final round of real-space optimisation was performed against the standard sharpened map (B-factor -73.7), followed by the refinement of B factors only in the unsharpened map. To clarify these details, the following sentence has been added to the methods: “Rebuilding and real space refinement including global minimisation, and local grid search were performed against the sharpened map (B-factor of –73.7), but the final refinement of B-factors only was performed using the unsharpened map to ensure appropriate B factor distributions.”

The map displayed in figures 1 and 2 is the sharpened map and this detail has been added to the figure legends for figures 1 and 2.

References

Baker, J. L., Biais, N., & Tama, F. (2013). Steered molecular dynamics simulations of a type IV pilus probe initial stages of a force-induced conformational transition. *PLoS Computational Biology*, 9(4), e1003032.

Cuendet, M. A., & Michielin, O. (2008). Protein-protein interaction investigated by steered molecular dynamics: the TCR-pMHC complex. *Biophysical journal*, *95*(8), 3575-3590.

Dong et al., (2017) Force interacts with macromolecular structure in activation of TGF-β. Nature, 542:55-9.

Evans & Kinoshita (2007). Using force to probe single-molecule receptor-cytoskeletal anchoring beneath the surface of a living cell. Methods Cell Biol. 83:373-96.

Fontana, L. et al. Fibronectin is required for integrin alphavbeta6-mediated activation of latent TGF-beta complexes containing LTBP-1. FASEB J 19, 1798-808 (2005).

Hinck et al, (2016) Structural Biology and Evolution of the TGF-β Family. Cold Spring Harbor Perspectives in Biology. 8(12).

Isogai, Z. et al. Latent transforming growth factor beta-binding protein 1 interacts with fibrillin and is a microfibril-associated protein. J Biol Chem 278, 2750-7 (2003).

Jurukovski et al., Methods in Molecular Medicine (2005) Methods for Measuring TGF-β Using Antibodies, Cells, and Mice. 117:161-75

Lockhart-Cairns, M.P. et al. Latent TGFβ complexes are transglutaminase cross-linked to fibrillin to facilitate TGFβ activation. Matrix Biol 107, 24-39 (2022).

Nunes et al., JCB (1997) Latent Transforming Growth Factor-β Binding Protein Domains Involved in Activation and Transglutaminase-dependent Cross-Linking of Latent Transforming Growth Factor-β.

Radaev, S.et al. (2010) Ternary complex of transforming growthfactor-b1 reveals isoform specific ligand recognition and receptor recruitment in the superfamily. J. Biol. Chem. 285, 14806–14814.

Rodriguez, R. A., Yu, L., & Chen, L. Y. (2015). Computing protein–protein association affinity with hybrid steered molecular dynamics. *Journal of Chemical Theory and Computation*, 11(9), 4427–4438.

Shi et al., (2011) Latent TGF-b structure and activation. Nature, 474:343-9.

Troilo et al (2016) Independent multimerization of Latent TGFβ Binding Protein-1 stabilized by cross-linking and enhanced by heparan sulfate. Sci Rep 6:34347.
